# Initial evidence on the relationship between the coronavirus pandemic and crime in the United States

**DOI:** 10.1186/s40163-020-00117-6

**Published:** 2020-05-18

**Authors:** Matthew P. J. Ashby

**Affiliations:** Jill Dando Institute of Security and Crime Science, University College London, 35 Tavistock Square, London, WC1H 9EZ UK

**Keywords:** COVID-19, Coronavirus, Crime, Crime trends

## Abstract

The COVID-19 pandemic led to substantial changes in the daily activities of millions of Americans, with many businesses and schools closed, public events cancelled and states introducing stay-at-home orders. This article used police-recorded open crime data to understand how the frequency of common types of crime changed in 16 large cities across the United States in the early months of 2020. Seasonal auto-regressive integrated moving average (SARIMA) models of crime in previous years were used to forecast the expected frequency of crime in 2020 in the absence of the pandemic. The forecasts from these models were then compared to the actual frequency of crime during the early months of the pandemic. There were no significant changes in the frequency of serious assaults in public or (contrary to the concerns of policy makers) any change to the frequency of serious assaults in residences. In some cities, there were reductions in residential burglary but little change in non-residential burglary. Thefts of motor vehicles decreased in some cities while there were diverging patterns of thefts from motor vehicles. These results are used to make suggestions for future research into the relationships between the coronavirus pandemic and different crimes.

## Introduction

The current coronavirus pandemic is drastically altering many aspects of life around the world. This article outlines initial evidence on how crime is changing, using data from a group of large cities in the United States. This evidence is necessarily limited and it will be possible to understand more detail as the pandemic progresses and more data become available. Nevertheless it is likely to be useful to consider the available evidence now, both to help the development of research questions and data collection for further research, and to provide the best-available evidence on a topic of substantial public interest.

**Fig. 1:**
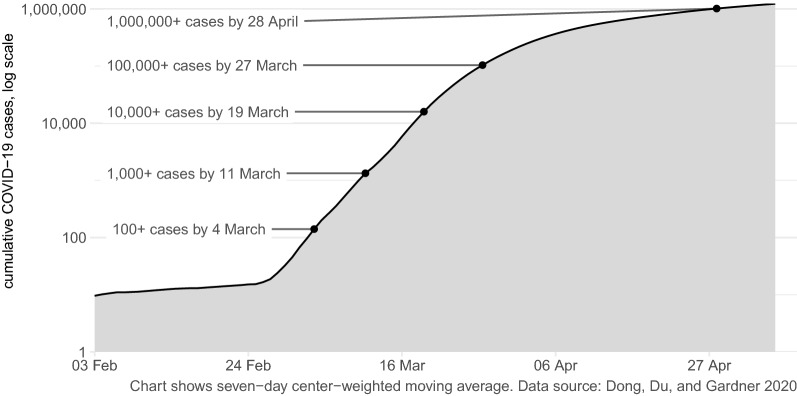
Number of confirmed COVID-19 cases in the United States over time. The number of confirmed cases may be understated due to limited testing capacity

The first cases of the COVID-19 disease caused by the novel coronavirus SARS-CoV-2 were reported in the city of Wuhan, China in December 2019. The first case in the United States was reported on 20 January 2020 in the suburbs of Seattle, Washington (Holshue et al. 2020). Within a week, cases had been reported in Illinois, Arizona and California, and then in every state except Wyoming by 18 March (Dong et al. 2020). By 11 May 2020, there had been about 1,345,000 reported cases of coronavirus in the United States (see Fig. 1) and 80,554 people had died.

Local, state and federal agencies responded to the pandemic with measures designed to slow the spread of disease and minimize the intensity of peak demand for potentially scarce healthcare resources (US Department of Health and Human Services 2020). One of the main mechanisms for achieving these goals was ‘social distancing’, i.e. reducing person-to-person transfer of the virus by minimizing the circumstances in which people were in close contact with one another (Qualls et al. 2017). This was done by closing schools, cancelling public events (including almost all sports and public entertainment) and advising or ordering people to stay home except for essential trips. California became the first to issue a state-wide stay-at-home order on 19 March, with Illinois, New Jersey and New York following over the next 3 days. Thirty-four more states issued similar orders within the following 2 weeks (Mervosh et al. 2020), during which time all 50 states had ordered or recommended school closures (Education Week 2020).

Patterns of activity during the pandemic were likely to be different from normal for at least three reasons. First, some people’s activities were directly affected by the virus, e.g. because they were sick or caring for those who were. Second, activities may have been affected by people’s fear of infection, especially those who have underlying medical conditions that put them at greater risk. Third, a great many people’s activities will have been influenced by government actions to slow the spread of the virus.

Data from the location tracking service Foursquare (2020) show that by late March visits to shopping malls, clothing stores, gyms, bars, restaurants, airports and hotels had all fallen by more than 50% compared to mid February, while visits to offices had fallen 37%. Conversely, people were likely to be spending more time at home, since children were not at school, many service-sector employees were furloughed and employers encouraged staff to work from home. Some public places also stand out as becoming busier during the early weeks of the pandemic: Foursquare (2020) data show increases in visits to grocery, hardware, liquor and drug stores beginning in early March. The same source also shows increases in visits to parks and hiking trails, although this may a seasonal effect as winter gave way to spring.

The routine activities approach (Cohen and Felson 1979) can be used to understand relationships between crime and people’s daily activities. For a crime to occur, one or more motivated offenders must come into contact with a suitable target in the absence of controllers (guardians of targets, managers of places and handlers of offenders) who make committing the crime more difficult (for a recent description of this framework, see Eck and Madensen 2015). For example, a purse-snatching in a public park requires not only that a purse and a purse-snatcher be in the same place at the same time, but that other park-goers (potential target guardians) do not intervene, that the park authorities (place managers) have not taken steps to create an environment that makes purse-snatching more difficult, and that the offender’s girlfriend (a potential offender handler) has not dissuaded them from offending by threatening to end the relationship if they do.

The coronavirus pandemic may have changed activities of each of these actors in different ways. Fewer people on the street may have meant fewer targets for street robbery, but also fewer guardians. More people in grocery stores may mean more potential shoplifters, but queuing systems and more staff refilling depleted shelves may have led to more-active place management. Social distancing advice to keep 6-feet apart from others may have made pick-pocketing almost impossible, while only having minor effects on other types of crime. The rapid development and complexity of changes to large parts of daily life mean predicting the direction or magnitude of any changes in crime is difficult.

### Existing evidence

To the author’s knowledge, there are no published empirical studies on the influence of crime on previous epidemics such as the 1918 influenza pandemic or the outbreaks of severe acute respiratory syndrome (SARS) in 2002–2004 or Middle East respiratory syndrome (MERS) since 2012. There are, however, other potential sources of evidence on how crime changes during sudden and widespread changes to routine activities.

Several scholars have studied changes in crime during and after natural disasters, particularly Atlantic hurricanes (Elmes et al. 2014). Disasters cause widespread changes to routine activities through deaths, evacuations and disruption of working and leisure patterns, as well as through damage to the physical environment. Lebeau (2002) and Frailing and Harper (2017) found increases in burglaries in the immediate aftermath of hurricanes in the United States, possibly due to more properties being unattended if residents have evacuated or been hospitalized (Leitner and Helbich 2011). Studies of New Orleans after Hurricane Katrina found an increase in homicide, possibly due to the disruption and subsequent informal reorganization of drug markets (Frailing and Harper 2017). However, Roman et al. (2007) found that violence more generally in New Orleans actually fell after Katrina, before rising to a higher level than before the hurricane. Leitner et al. (2011) found that violence was broadly stable in other Louisiana parishes hit by the storm. Varano et al. (2010) studied violent and non-violent crime rates in cities that hosted large numbers of Katrina evacuees and found no widespread or pervasive changes. Prelog (2016), studying associations between crime and the frequency and severity of natural disasters at the county level, found disasters were associated with higher property crime but no change in violence. For further discussion of crime in the context of natural disasters, see Frailing and Harper (2017).

Another potential source of evidence comes from changes in crime associated with the large-scale changes in routine activities caused by major sporting events. These affect routine activities by bringing large numbers of people into the event area, disrupting traffic and public transport, and gathering people to watch the event on television. As with crime during natural disasters, studies of crime during major events have produced mixed results. Campaniello (2013) found that the football World Cup was associated with increases across several types of property crime (including burglary and pick-pocketing), while Kurland et al. (2014) concluded large football matches at Wembley Stadium in London were associated with increases in both violent crime and theft in the immediate area. However, Breetzke and Cohn (2013) found no city-wide increase in violence associated with football matches and no increase in burglary even in the neighborhood of the stadium. Baumann et al. (2012) reported that a city hosting the Olympic Games was associated with a 10% increase in property crime, but hosting the Super Bowl was associated with a 2.5% decrease in violence. For a recent discussion of crime associated with major sporting events, see Piquero et al. (2019).

The coronavirus pandemic is different in nature to the natural disasters and sporting events analyzed by previous researchers. In particular, a pandemic is a ‘slow-onset’ emergency that emerges over time and then varies in its impact (United Nations Office for the Coordination of Humanitarian Affairs 2011). It is also different in that, unlike a hurricane or tornado, coronavirus has left the physical environment largely untouched. It has not, for example, destroyed buildings or cut off electricity. Instead, the changes caused by the pandemic have largely related to human activity.

The goal of this study was to make an initial estimate of how the frequency of different types of crime changed during the coronavirus pandemic. Given the evolving nature of the situation, the complexity of potential interactions between actors in the routine activities framework and the lack of any previous research on crime during pandemics, this study did not attempt to test specific hypotheses but to explore how crime varied throughout the early months of the pandemic.

## Data and methods

This report uses data from 16 large cities or urban counties in the United States: Austin, TX, Baltimore, MD, Boston, MA, Chicago, IL, Dallas, TX, Los Angeles, CA, Louisville, KY, Memphis, TN, Minneapolis, MN, Montgomery County, MD, Nashville, TN, Philadelphia, PA, Phoenix, AZ, San Francisco, CA, Tucson, AZ and Washington, DC.^1^ These cities were chosen because they all provide public access to an incident-level extract of police-recorded crime data that is updated at least weekly. This allows monitoring of emerging crime trends that would be obscured in annual crime statistics bulletins produced by many agencies. Open crime data have some limitations (Ashby 2019) but have been used successfully in previous research on crime trends (Tompson et al. 2014).

The complex interactions of targets, offenders and controllers mean it is likely that the COVID-19 pandemic may have different relationships with different types of crime. To increase the robustness of the results, this study focused on crime types which are known to be both relatively likely to be reported to police and relatively unaffected by variations in police recording practices. These types were:

1. *Serious assaults in public places* including homicides and aggravated assaults in commercial, leisure, retail and transportation settings as well as in public open spaces and streets.
2. *Serious assaults in residences* including homicides and aggravated assaults in houses, apartments etc but excluding institutional residential settings such as hospitals, hotels and prisons.
3. *Residential burglaries* (excluding auto burglaries) occurring in residences, excluding institutional residences.
4. *Non*-*residential burglaries* (excluding auto burglaries) occurring anywhere other than a residence.
5. *Theft of vehicles*
6. *Theft from vehicles* including auto burglaries and thefts of auto parts.

Understanding relationships between COVID-19 and crime requires some estimate of how much crime would be expected to occur in the absence of the pandemic. This is difficult because so many factors influence how much crime occurs. A number of media outlets have attempted to understand changes in crime during the pandemic by making week-to-week or year-on-year comparisons of weekly or monthly crime counts. However, these comparisons risk drawing false conclusions because they ignore long-term trends (which affect year-on-year comparisons) and seasonal variations (which complicate week to week comparisons). Perhaps most importantly, these comparisons take no account of random variation, even though crime counts over short periods typically show a relatively large amount of statistical noise (Gorr et al. 2003). For example, on 27 March *The Washington Post* reported that both burglaries and assaults in New York City had dropped by 18% compared to the previous week (Jacobs and Devlin 2020). But across the 16 cities for which data were available for the current study, the absolute week-to-week change in burglary was greater than 18% in an average of 16 weeks each year between 2016 and 2019 and the absolute change in serious assaults greater than 18% in an average of 20 weeks each year. That apparently newsworthy changes in crime are actually commonplace might help explain why 6 days later on 2 April, the *New York Post* was able to report that felonies in New York City had increased 12% year-on-year “despite coronavirus” (McCarthy, 2020).

To better estimate the expected frequency of crime in the absence of the pandemic, this report uses seasonal auto-regressive integrated moving average (SARIMA) models of the frequency of different crime types in each city between 1 January 2016^2^ and the first confirmed case of COVID-19 in the United States on 20 January 2020 (Holshue et al. 2020). The models incorporate both a dummy trend variable and 51 dummy variables for weekly seasonal terms, along with a variable denoting whether the week included a US federal public holiday. Separate models were estimated for each type of crime in each city, with the dependent variable in each model being the number of crimes recorded each week.

SARIMA models require selection of the number of periods to use in calculating the seasonal and non-seasonal auto-regressive (AR) and moving-average (MA) terms in each model. The current study selected these terms automatically using the algorithm outlined by Hyndman and Khandakar (2008) as implemented in the fable package (O’Hara-Wild et al. 2020) in R version 3.6.1 (R Core Team. 2019). This estimates multiple models with different values for the SARIMA terms and then chooses the model which minimizes the Akaike information criterion (AIC), an estimator of prediction error. The selected model for each crime type in each city is shown, together with descriptive statistics, in Table 1.

**Table 1:** Terms used in SARIMA models for estimating crime frequency without coronavirus. All models had zero seasonal MA periods

Crime type	City	Min	Mean	SD	Max	AR periods	MA periods	Seasonal AR periods	Degrees of freedom	MASE
Serious assaults in public	Austin, TX	36	65	11	89	4	0	1	152	0.57
	Baltimore, MD	20	65	18	113	1	1	1	154	0.55
	Dallas, TX	16	36	10	64	0	0	1	106	0.55
	Los Angeles, CA	130	210	31	281	3	0	1	153	0.58
	Louisville, KY	1	9	3	18	2	0	1	154	0.61
	Montgomery County, MD	0	5	2	12	0	1	1	130	0.56
	Nashville, TN	5	62	33	169	3	0	1	154	0.34
	Phoenix, AZ	23	42	8	63	0	0	1	156	0.54
Serious assaults in residences	Austin, TX	58	89	12	136	2	1	1	153	0.56
	Baltimore, MD	3	35	8	60	2	0	1	154	0.58
	Dallas, TX	6	19	7	45	0	1	1	105	0.47
	Los Angeles, CA	68	106	16	162	1	1	1	154	0.57
	Louisville, KY	2	11	6	36	1	1	1	154	0.46
	Montgomery County, MD	1	7	3	17	0	0	1	131	0.55
	Nashville, TN	6	51	23	122	1	2	1	153	0.31
	Phoenix, AZ	25	49	10	79	1	1	1	154	0.44
Residential burglary	Austin, TX	20	53	13	101	5	0	1	151	0.44
	Baltimore, MD	15	80	23	139	2	0	1	154	0.41
	Boston, MA	10	28	8	52	2	0	1	154	0.48
	Chicago, IL	202	376	82	584	4	1	1	151	0.35
	Los Angeles, CA	145	209	28	293	4	0	1	152	0.46
	Louisville, KY	38	70	16	111	3	0	1	153	0.49
	Memphis, TN	59	123	22	198	1	1	1	154	0.52
	Minneapolis, MN	15	51	16	107	2	0	1	154	0.46
	Montgomery County, MD	4	21	6	40	3	0	1	128	0.46
	Phoenix, AZ	86	143	28	219	1	0	1	155	0.39
	San Francisco, CA	28	53	10	81	1	1	1	154	0.45
Non-residential burglary	Austin, TX	11	33	9	59	1	1	1	154	0.56
	Baltimore, MD	20	49	18	162	2	1	1	153	0.44
	Chicago, IL	42	89	21	174	2	0	1	154	0.51
	Los Angeles, CA	69	119	20	184	1	1	1	154	0.52
	Louisville, KY	11	31	8	57	1	1	1	154	0.56
	Memphis, TN	13	42	12	87	1	1	1	154	0.47
	Minneapolis, MN	2	13	5	33	1	1	1	154	0.56
	Montgomery County, MD	0	7	5	52	0	0	1	131	0.57
	Philadelphia, PA	13	26	8	70	5	0	1	151	0.58
	Phoenix, AZ	24	53	9	80	2	0	1	154	0.47
	San Francisco, CA	14	51	25	111	1	1	1	155	0.23
Theft of vehicle	Austin, TX	23	45	11	78	1	1	1	154	0.49
	Baltimore, MD	46	82	15	145	2	0	1	154	0.50
	Chicago, IL	264	398	65	596	2	0	1	154	0.50
	Los Angeles, CA	255	344	37	462	3	0	1	153	0.41
	Louisville, KY	47	78	13	118	4	0	1	152	0.45
	Memphis, TN	38	79	17	163	3	1	1	152	0.41
	Minneapolis, MN	15	44	14	131	1	1	0	155	0.51
	Montgomery County, MD	7	17	5	41	0	0	1	131	0.58
	Philadelphia, PA	23	44	9	69	1	0	1	155	0.54
	Phoenix, AZ	89	132	16	181	0	0	1	156	0.54
	San Francisco, CA	68	105	19	157	1	1	1	154	0.45
	Tucson, AZ	23	43	10	74	1	1	1	154	0.46
	Washington, DC	25	46	11	90	1	2	1	153	0.50
Theft from vehicle	Austin, TX	120	202	39	325	1	2	1	153	0.39
	Baltimore, MD	54	118	27	217	0	1	1	155	0.46
	Los Angeles, CA	537	632	41	743	2	0	1	154	0.49
	Louisville, KY	50	97	20	156	3	1	1	152	0.45
	Memphis, TN	82	149	31	228	1	1	1	154	0.52
	Minneapolis, MN	24	70	21	128	3	0	1	153	0.46
	Montgomery County, MD	25	85	18	141	1	1	1	129	0.42
	Philadelphia, PA	149	258	39	369	1	1	1	154	0.52
	San Francisco, CA	337	545	84	743	1	1	1	154	0.33
	Tucson, AZ	40	73	15	116	2	1	1	153	0.38
	Washington, DC	108	214	41	320	2	0	1	154	0.38
Personal robbery	Baltimore, MD	47	90	20	164	0	2	1	154	0.51
	Boston, MA	6	18	6	42	2	3	1	151	0.55
	Chicago, IL	170	344	81	544	2	0	1	154	0.41
	Dallas, TX	0	62	18	108	0	0	1	110	0.57
	Los Angeles, CA	119	168	19	227	0	0	1	156	0.53
	Louisville, KY	5	17	6	36	3	0	1	153	0.53
	Memphis, TN	24	53	13	86	0	0	1	156	0.58
	Minneapolis, MN	7	26	9	51	2	2	1	152	0.44
	Montgomery County, MD	0	7	4	21	0	1	1	130	0.55
	Philadelphia, PA	49	98	18	159	0	0	1	156	0.54
	San Francisco, CA	8	20	5	34	0	1	1	155	0.58
	Tucson, AZ	3	12	4	24	1	2	1	153	0.56

The final column of Table 1 shows the mean absolute scaled error (MASE) of each model (Hyndman and Koehler 2006). MASE values smaller than 1 indicate that the chosen model performs better (i.e. has a smaller error) than a ‘naïve’ model in which crime frequency is simply forecast to be the same as in the previous week. Comparing the chosen model to a naïve model is particularly relevant in this case because a naïve forecasting model is functionally identical to attempting to identify changes in crime frequency solely through week-to-week comparisons, as in the media stories referenced above. In every case, the predictive error of the chosen SARIMA model was less than that of the equivalent naïve model.

The SARIMA models were used to forecast how many crimes in each category would be expected to occur in each city in the weeks after 20 January 2020 in the absence of the virus or any other change in crime trends. These forecasts acted as a synthetic comparison group against which to compare the actual frequency of crime during the pandemic. Due to the large number of such comparisons (one for each crime type in each city in each week), some actual frequencies would be expected to be different from the forecast frequency by chance. Controlling for this (for example by adjusting the significance threshold) is not straightforward, since the data are temporally autocorrelated. For the purposes of this analysis, actual crime counts outside the 99% confidence interval (rather than the more conventional 95%) of the forecast crime count were considered to be significantly different to the expected count of crime in the absence of the virus, but only if the actual count was outside the confidence interval for at least two consecutive weeks. While it is not possible to exclude other sources of variation that may have occurred at the same time as the beginning of the pandemic, it is unlikely that any change unrelated to the virus would occur simultaneously in all the cities under study.

One alternative approach to modelling the influence of an event on the frequency of crime over time is the interrupted time series design, in which the presence of a relationship between the event and crime can be identified using a dummy variable denoting whether a crime count occurred before or after the event. This approach was not used here because the coronavirus pandemic was a slow-onset emergency that may have a relationship to crime that changes over time (Frailing and Harper 2017). Interrupted time series designs are not appropriate in such circumstances because they assume that a clear distinction can be made between crimes occurring before and after the event under study (Kontopantelis et al. 2015). Such designs also provide only a single overall measure of the relationship between crime and an external event, whereas it is important to be able to identify evolving relationships caused by, for example, people adjusting to a new normal as an emergency unfolds (Varano et al. 2010).

The data and annotated R code for this analysis are available at https://osf.io/ef4dw/. COVID-19 case data were taken from Dong et al. (2020) using the COVID19 R package (Guidotti and Ardia 2020). The online supplementary material for this article includes a summary of the data made available (Additional file 1) and the co-efficients for each SARIMA model (Additional file 2).

## Results and discussion

**Fig. 2:**
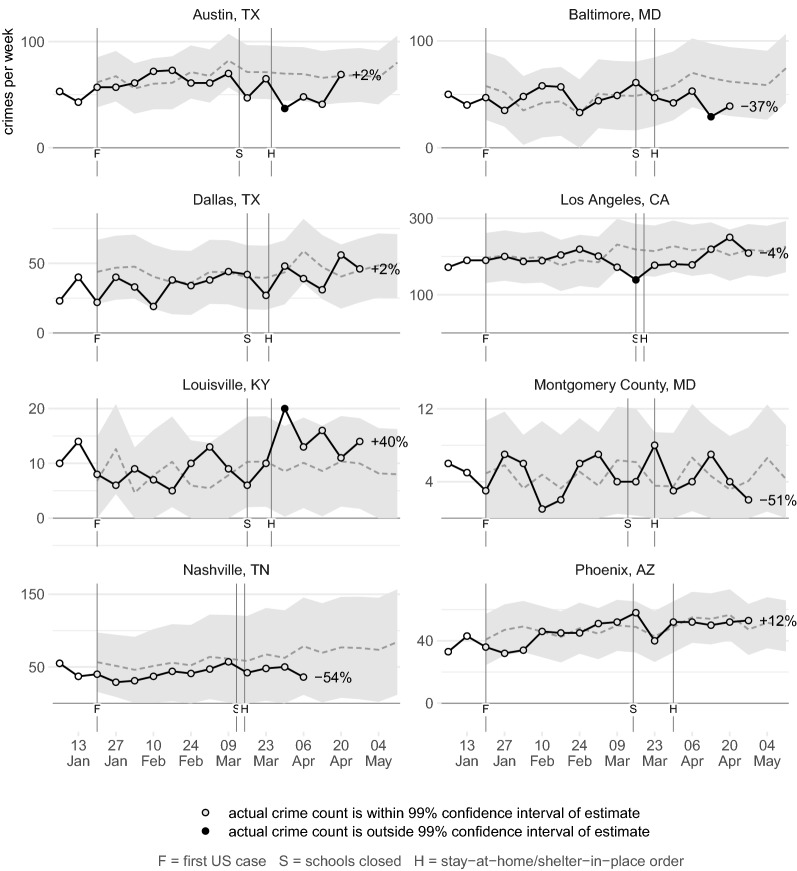
Frequency of serious assaults in public during coronavirus pandemic compared to estimates of the number of assaults that would have occurred under normal conditions

**Fig. 3:**
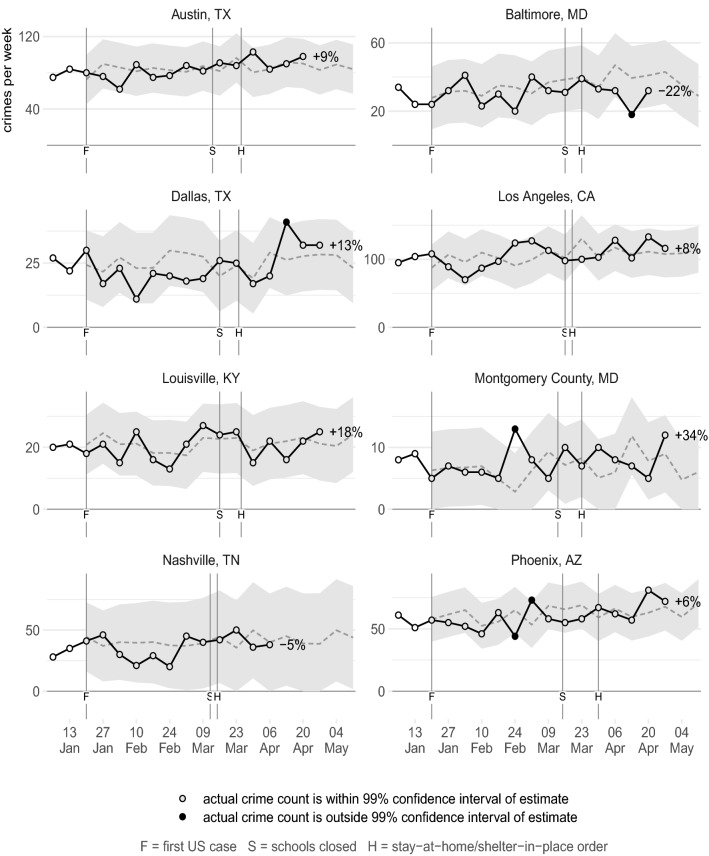
Frequency of serious assaults in residences during coronavirus pandemic compared to estimates of the number of assaults that would have occurred under normal conditions

**Fig. 4:**
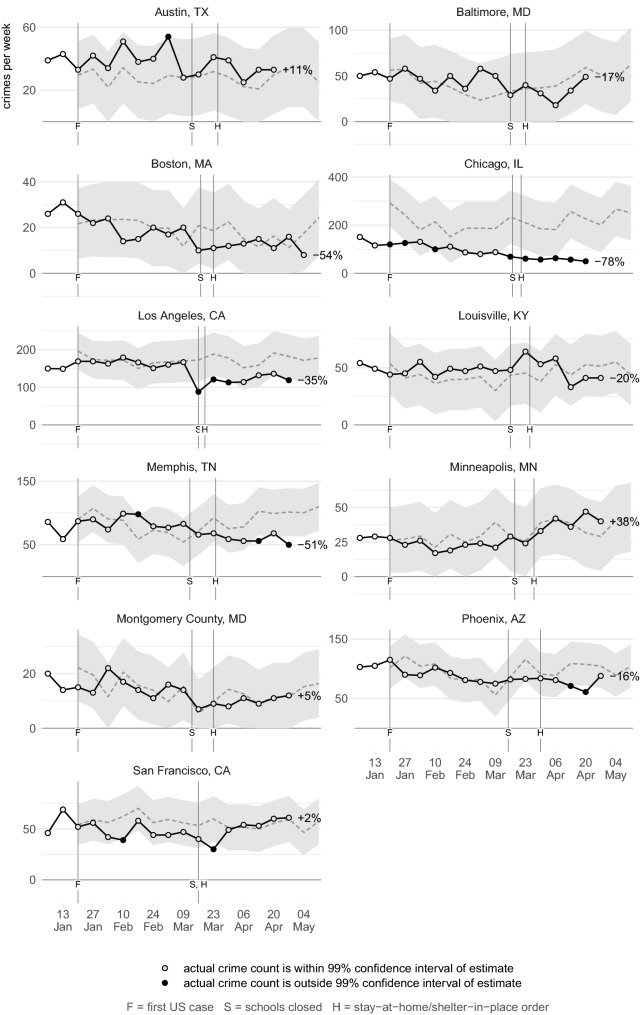
Frequency of residential burglaries during coronavirus pandemic compared to estimates of the number of assaults that would have occurred under normal conditions

**Fig. 5:**
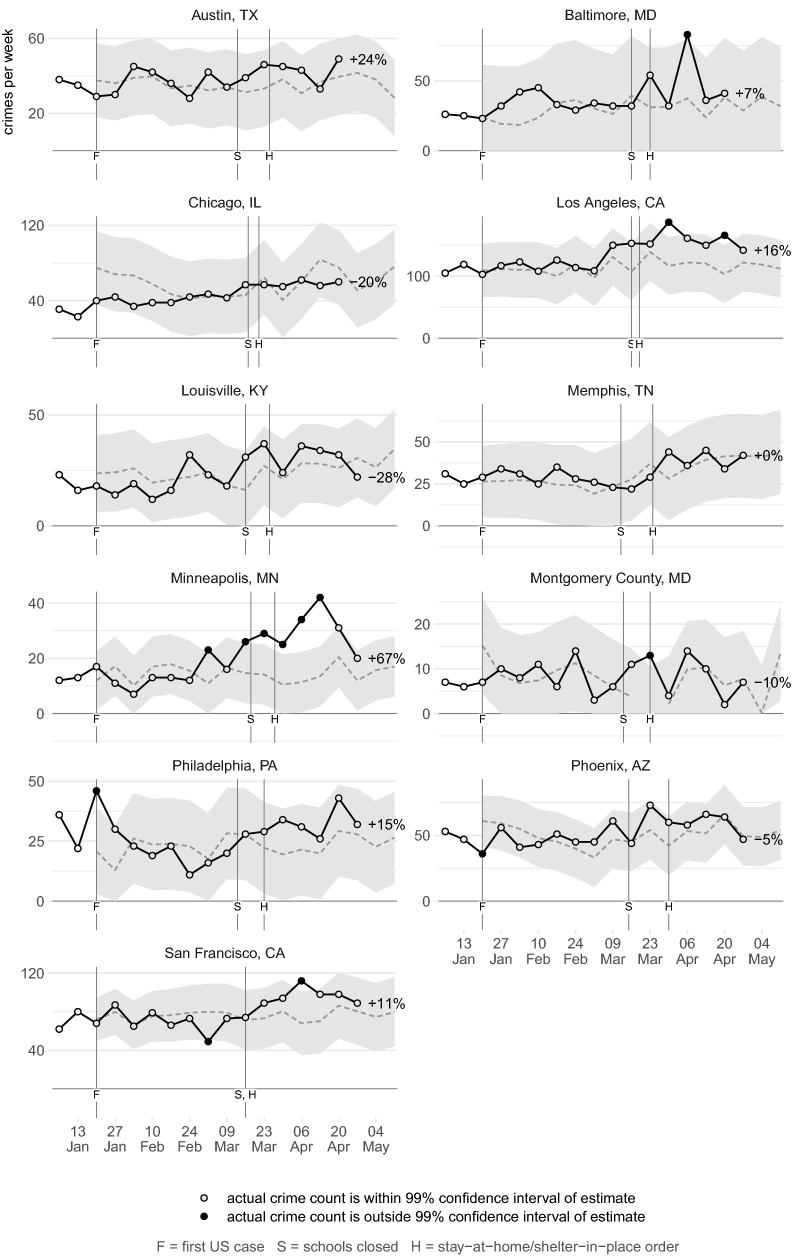
Frequency of non-residential burglaries during coronavirus pandemic compared to estimates of the number of assaults that would have occurred under normal conditions

**Fig. 6:**
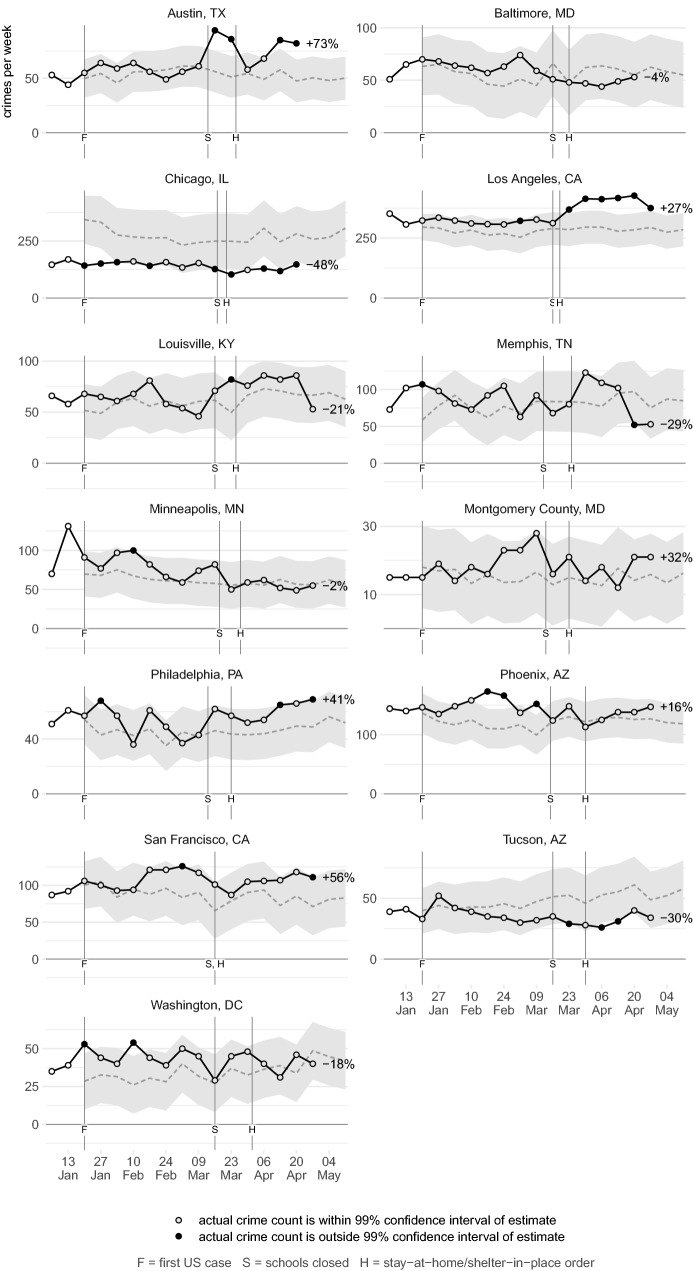
Frequency of thefts of vehicles during coronavirus pandemic compared to estimates of the number of assaults that would have occurred under normal conditions

**Fig. 7:**
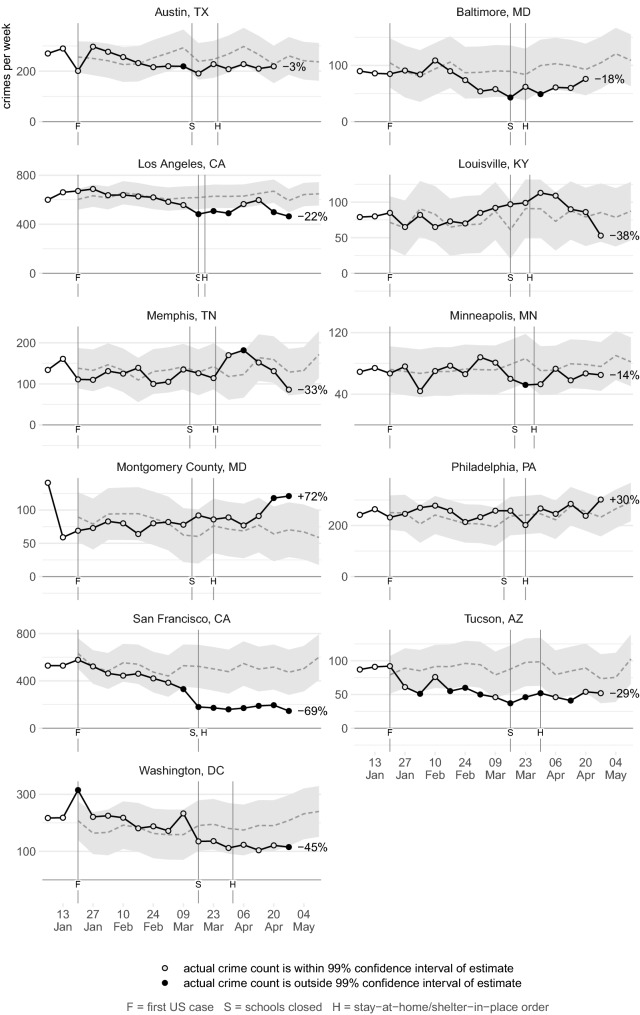
Frequency of thefts from vehicles during coronavirus pandemic compared to estimates of the number of assaults that would have occurred under normal conditions

Figures 2, 3, 4, 5, 6, 7 show the observed frequency of crimes of each type (the black line in each figure) along with the forecast frequency based on the corresponding SARIMA model. The model forecast is shown as a dashed line, with the associated 99% confidence interval shown as a grey band. Also shown on each figure are the date of the first COVID-19 case in the United States (which marks the end of the data with which the forecasts were made), together with the dates on which the relevant city or state government closed schools and issued a stay-at-home order. The percentages shown in the figures represent the percentage difference between the frequency of crime forecast by the model and the actual frequency. The figures include the observed frequency of crime up to the week ending 10 May 2020 inclusive, the most-recent complete week of available data at the time of writing.

Before considering patterns in individual types of crime, some general features across types can be identified. The first is that comparing actual crime to expected crime based on a forecasting model assumes that the frequency of crime would (in the absence of coronavirus) have continued to be shaped by the same forces as in previous years. While this will be true in many cases, it is likely that—for some types of crime in some cities—other factors will have lead crime frequency in 2020 to diverge from what might have been expected based on the frequency of crime in previous years. This can be seen, for example, in thefts of vehicles in Chicago, which were below the confidence interval even in late January. This is an inherent limitation of the synthetic comparison approach used in this study, but is likely to be unavoidable in the context of a global pandemic that meant there were no unaffected places to provide comparison data. The MASE values discussed above also show that, despite this limitation, the current approach is likely to be less error-prone than simple month-to-month and similar comparisons.

The second general feature of note is that there is no apparent relationship between COVID-19 and crime of any type between the first US case on 20 January and the beginning of March, with possible effects emerging only later. This is unsurprising, since although by 1 March there had been 2500 cases of COVID-19 globally, only 30 of those were in the United States (across seven states) and there had been only one US deaths. By the time the World Health Organization declared COVID-19 as a pandemic on 11 March (World Health Organization 2020), the number of US cases had increased to 1105 in 40 states, while from 22 March onward there were more new cases in the US each day than in any other country. Future researchers should therefore ensure their analytical methods do not conflate data from before and after early March, which might risk masking any effect.

The third general finding from the figures presented below is that no type of crime changed uniformly in all of the cities under study. This should also be unsurprising, since crime is known to be heavily context dependent and the contexts of different cities vary considerably. To give two simple examples, median household income in San Francisco is more than double that in Baltimore, while population density is more than six times higher in Chicago than in Louisville (US Census Bureau 2020). Understanding the context underlying different relationships between coronavirus and crime is likely to be an important question for future researchers in this area.

### Serious assaults

Figure 2 shows that the frequency of assaults in public places was below that estimated by the SARIMA model in five of eight cities, but in no case was the frequency consistently outside the 99% confidence interval of the model. This suggests the observed variation was within what would be expected based on the frequency of serious assaults in previous years. In some cases the confidence intervals are quite wide, reflecting the substantial week-to-week changes in crime frequency mentioned above. Overall, the frequency of serious assaults appears not to have systematically changed during the early weeks of the coronavirus pandemic.

This is theoretically unexpected, given the data discussed above showing that the amount of time people spend in public places decreased substantially during the pandemic. This change in people’s routine activities might be expected to have led to a decrease in assaults in public, but this does not appear to have happened. Further research into this question may need to explore any changes in who is involved in serious assaults during the pandemic. Since serious violence is known to be concentrated among a small number of persistent offenders and repeat victims (Jennings et al. 2012), and that offenders who commit serious offences are also more likely to commit minor infractions such as breaching stay-at-home orders (Roach 2018), it may be that those involved in serious assaults were no less likely to be on the streets during the pandemic than before. Future research should also look at the types of public place in which assaults have occurred: it may be that during the pandemic public assaults were displaced from those facilities seeing fewer people (such as retail malls) to those seeing more (such as parks).

In the first weeks of the pandemic there was substantial concern among lawmakers and practitioners that the stay-at-home orders issued by many cities and states would lead to an increase in domestic assaults because victims would be trapped at home with their abusers (Elinson and Chapman 2020; Taub 2020), with some media reporting that such increases had occurred. For example, in an online news article Tolan (2020) reported that of “20 large metropolitan police departments that provided data to CNN, nine saw double-digit percentage jumps in domestic violence cases or 911 calls in March, either compared to the previous year or to earlier months in 2020”. Notwithstanding that month-to-month comparisons between February are flawed because March has 11% more days than February, such comparisons ignore that 11 of the departments did not see an increase and month-to-month comparisons take no account of the variability of crime over time.

Figure 3 shows that, at least during the early part of the epidemic, concerns of a surge in domestic violence may have been unfounded. The observed frequency of serious assaults in residences (a category not necessarily identical to domestic violence) was above that forecast by the model in five cities and below the model forecast in another three, but in every case the observed frequency was within the confidence interval of the model estimates.

Understanding the nature of domestic abuse from police data is always problematic because many incidents are not reported to police. During lockdowns, this problem may be exacerbated because victims might find it harder to get in touch with those who can help them. For this reason, the present study looked at only serious assaults, which are more likely to be reported than minor assaults. Nevertheless the present result should be treated with caution: as yet there is no evidence of a systematic increase in serious assaults in residences in large US cities, but it is possible that this is due to limitations in the data. The current findings should be validated against survey-based measures of victimization that take longer to become available (and may be impossible to collect during lockdowns).

### Burglary

Figure 4 shows the frequency of residential burglaries across 11 cities. The frequency of burglary is below the model estimate in eight cities, although in five of those it remains within the 99% confidence interval. In Chicago, Los Angeles and Memphis the frequency of burglary decreased below the lower confidence interval, with the frequency around half of what was forecast. In Chicago and Los Angeles, these reductions began during the week in which schools closed and stay-at-home orders were issued in those cities and were sustained each week afterwards. This means that, for example, in the 4 weeks after a stay-at-home order was issued in Chicago, there were approximately 940 fewer residential burglaries than would have been expected based on the model forecast. This is consistent with the routine activities approach, since stay-at-home orders mean people are more likely to be at home and so able to act as guardians of their property.

Not all cities saw a decrease in residential burglary. For example, burglary in Austin, Louisville and Minneapolis has largely tracked the forecasts produced by the respective models. The inconsistency of these trends across different cities is likely to be an important issue to explore in later research that can make use of more extensive data.

Figure 5 shows the frequency of non-residential burglaries. In ten of 11 cities, non-residential burglary was higher after lockdown measures began than forecast, but in only one (Minneapolis) was the difference significant. This lack of a consistent increase may be theoretically unexpected, since the closure of many businesses and the increased number of people working from home might be expected to have decreased guardianship around many non-residential buildings. Further research into this area might usefully disaggregate non-residential burglary into different types of premises. It may be that non-residential burglary is particularly concentrated at establishments (such as pharmacies and liquor stores) that remained open throughout the lockdown period and so had the same levels of guardianship as previously.

### Vehicle theft

Figure 6 shows that the frequency of thefts of motor vehicles had divergent patterns across cities. In Austin and Los Angeles, vehicle theft increased significantly in the weeks after lockdowns began, while in another seven there were smaller increases that remained within the confidence interval. Conversely, in Chicago and Tucson vehicle theft decreased significantly. Figure 7 shows the frequency of thefts from vehicles, which decreased in eight cities, in three of them significantly. Some of these decreases were particularly large: between 9 March and 27 April there were around 2520 (62%) fewer thefts from motor vehicles in San Francisco than forecast by the SARIMA model, and 870 (17%) fewer than forecast in Los Angeles.

One of the main drivers of vehicle theft is likely to be the availability of unattended vehicles to steal or steal from. The distribution of vehicles may have changed during the pandemic, for example if more cars were parked outside the homes of furloughed people and fewer parked in downtown parking lots. Future research could therefore consider the changing distribution of thefts in different neighborhoods, e.g. did vehicle theft increase in suburbs while decreasing in business districts.

## Conclusion: suggestions for future research

At present, data are only available for the early months of the COVID-19 pandemic—the first US case was reported only 16 weeks prior to the time of writing. While future research will be able to understand the relationships between coronavirus and crime in greater detail, reporting the available evidence now is valuable to avoid potential spurious conclusions derived from simple week-to-week or year-on-year comparisons. These initial results also help identify questions for future research.

Overall, the present research found no significant changes in the frequency of serious assaults either in public or in residences (contrary to concerns among practitioners and policy makers), reductions in residential burglary in some (but not all) cities, little change in non-residential burglary (except in Minneapolis), decreases in thefts from vehicles in some cities, and diverging patterns of thefts of vehicles. It is noteworthy, however, that in no case were the patterns the same across all the cities under study.

Since there were clear differences across crime types in the relationships between the pandemic and crime, it will be important for future research on these relationships to study disaggregated crime types. Studying crime as an undifferentiated whole is almost always inadvisable (Cornish and Smith 2012) but the clear differences between crime types seen here suggest researchers should consider individual types disaggregated by location and victim type. Similarly, the differences seen across cities highlight the importance of studying the relationships between COVID-19 and different crime types in multiple settings. Much criminological research draws conclusions based on data from only one city (Ashby 2019), but obtaining a robust understanding of any relationships in the present case—and of the limitations of those relationships—is likely to require data from multiple cities or other areas. While the present study was able to access data only from large cities in the United States, future studies should analyse these research questions across countries, as well as in suburban and rural areas.

The present study focused solely on changes in crime immediately after cities and states implemented lockdowns to slow the progress of the epidemic. Future studies with access to more data will be able to understand how the patterns identified here will change over time, for example if increases or decreases in crime decay back to the level that would have been expected had the virus not occurred.

The changes to society caused by the COVID-19 pandemic are some of the largest and most sudden to happen in the United States in several decades. They provide a valuable opportunity both to test criminological theory and to inform practice. This article has used data from the initial wave of the pandemic to identify potential patterns in different types of crime in different cities. Future research into coronavirus and crime should use these findings to help identify more-detailed research questions and priorities for primary data collection.

## Supplementary information

**Additional file 1.** Summary of data accompanying this article.

**Additional file 2.** SARIMA model co-efficients.

## Data Availability

The data and annotated R code used in this article are available under a Creative Commons Attribution License in the Open Science Framework repository at https://osf.io/ef4dw/.

## Footnotes

1 Montgomery County – just outside Washington, DC – is not a single city, but a suburban county with a comparable population and a single county-wide police department.

2 Crime-count data from Dallas began on 28 November 2016 and data for Montgomery County began on 4 July 2016.
