# Supplementary material for: Initial evidence on the relationship between the coronavirus pandemic and crime in the United States
Source: Crime Sci. 2020 May 18;9(1):6. doi: 10.1186/s40163-020-00117-6 (PMC7233195; doi:10.1186/s40163-020-00117-6)
Supplement: Supplementary file 1 — Additional file 1. Summary of data accompanying this article. [file 40163_2020_117_MOESM1_ESM.pdf]

Coronavirus pandemic and crime in the United States: data summary

Matthew P J Ashby, Jill Dando Institute of Security and Crime Science, University College London

15 May 2020

The article 'Initial evidence on the relationship between the coronavirus pandemic and crime in the United States' uses seasonal auto-regressive integrated moving average (ARIMA) models to estimate the frequency of crime in large US cities. The article is accompanied by data files and materials available on the Open Science Framework at `https://osf.io/ef4dw/`

Three data files are provided. All data are available under a Creative Commons Attribution (CC-BY) license. Annotated R code for processing the data are also provided.

Crime counts

The file `crime_counts.csv` at `https://osf.io/2qx87/` contains the data used to estimate the ARIMA models used in the article. Each row represents a weekly count of crimes in a particular category in a particular city. The data extend from 1 January 2019 to 19 January 2020. A sample of rows from the file:

city_name	category	week_beginning	holiday_in_week	actual_crimes
Austin, TX	residential burglary	2016-01-11	FALSE	58
Austin, TX	residential burglary	2018-09-03	TRUE	52
Austin, TX	serious assaults in public	2016-05-23	FALSE	76
Baltimore, MD	personal robbery	2018-02-05	FALSE	87
Baltimore, MD	serious assaults in public	2017-02-27	FALSE	31
Boston, MA	residential burglary	2019-10-21	FALSE	25
Chicago, IL	non-residential burglary	2017-06-19	FALSE	56
Chicago, IL	serious assaults in public	2018-12-17	FALSE	131
Dallas, TX	serious assaults in public	2017-01-02	TRUE	23
Dallas, TX	serious assaults in residences	2017-12-25	TRUE	23
Los Angeles, CA	personal robbery	2020-02-17	TRUE	145
Louisville, KY	non-residential burglary	2018-05-14	FALSE	38
Louisville, KY	theft of vehicle	2019-01-14	FALSE	84
Memphis, TN	residential burglary	2019-09-30	FALSE	108
Memphis, TN	theft from vehicle	2017-04-17	FALSE	172
Philadelphia, PA	non-residential burglary	2018-12-31	TRUE	24
Philadelphia, PA	theft from vehicle	2017-08-21	FALSE	349
Philadelphia, PA	theft of vehicle	2017-08-21	FALSE	48
San Francisco, CA	non-residential burglary	2020-04-06	TRUE	91
Tucson, AZ	personal robbery	2020-04-06	TRUE	7

The field `holiday_in_week` shows whether there was a US federal holiday in the relevant week.

Model co-efficients

The file `model_results.csv` at `https://osf.io/qfsd5/` contains estimates, standard errors and associated *p*-values for the variables in each model. Additional information about this file is available at `https://osf.io/pzbvh/`

A sample of rows from the file:

city_name	category	term	estimate	std.error	statistic	p.value
Atlanta, GA	serious assaults in public	MA(1)	0.11	0.07	1.49	0.139
Atlanta, GA	serious assaults in public	SAR(1)	–0.39	0.08	–4.85	<0.001
Atlanta, GA	serious assaults in public	linear trend	–0.01	0.00	–2.82	0.005
Atlanta, GA	serious assaults in public	week 2 of the year	–3.16	1.74	–1.82	0.071
Atlanta, GA	serious assaults in public	week 3 of the year	–1.28	1.84	–0.70	0.488
Atlanta, GA	serious assaults in public	week 4 of the year	–3.72	2.06	–1.81	0.072
Atlanta, GA	serious assaults in public	week 5 of the year	–2.89	1.86	–1.55	0.122
Atlanta, GA	serious assaults in public	week 6 of the year	–6.11	1.84	–3.32	0.001
Atlanta, GA	serious assaults in public	week 7 of the year	–1.30	1.84	–0.71	0.480
Atlanta, GA	serious assaults in public	week 8 of the year	–1.73	1.84	–0.94	0.351
Atlanta, GA	serious assaults in public	week 9 of the year	–6.36	2.16	–2.95	0.004
Atlanta, GA	serious assaults in public	week 10 of the year	–4.36	1.91	–2.28	0.024
Atlanta, GA	serious assaults in public	week 11 of the year	–0.78	1.85	–0.42	0.675
Atlanta, GA	serious assaults in public	week 12 of the year	–2.98	2.08	–1.43	0.154
Atlanta, GA	serious assaults in public	week 13 of the year	–4.98	1.87	–2.66	0.009
Atlanta, GA	serious assaults in public	week 14 of the year	–3.37	1.85	–1.82	0.071
Atlanta, GA	serious assaults in public	week 15 of the year	0.53	1.85	0.29	0.775
Atlanta, GA	serious assaults in public	week 16 of the year	–1.47	1.86	–0.79	0.431
Atlanta, GA	serious assaults in public	week 17 of the year	–4.12	2.07	–1.99	0.048
Atlanta, GA	serious assaults in public	week 18 of the year	–2.47	1.85	–1.34	0.183

Model forecasts

The file `model_forecasts.csv` at `https://osf.io/9yqu7/` contains the forecasts of the frequency of crime produced by the ARIMA models. Again, each row represents a weekly count of crimes in a particular category in a particular city. A sample of rows from the file:

city_name	category	week_beginning	holiday_in_week	estimated_crimes	estimate_lower_ci	estimate_upper_ci	actual_crimes
Austin, TX	non-reside ...	2020-03-02	FALSE	32.2	11.9	52.6	41
Austin, TX	residentia ...	2020-03-02	FALSE	29.4	5.2	53.7	54
Baltimore, ...	theft from ...	2020-04-13	FALSE	99.7	53.7	145.7	NA
Baltimore, ...	theft of v ...	2020-04-13	FALSE	60.7	29.4	92.1	NA
Boston, MA	non-reside ...	2020-03-16	FALSE	3.3	0.0	11.8	NA
Boston, MA	residentia ...	2020-02-10	FALSE	23.5	7.0	40.1	13
Chicago, I ...	non-reside ...	2020-04-13	FALSE	83.8	44.1	123.5	NA
Chicago, I ...	serious as ...	2020-03-23	FALSE	253.8	156.0	351.5	4
Chicago, I ...	serious as ...	2020-03-02	FALSE	207.5	151.0	264.0	26
Los Angele ...	non-reside ...	2020-03-02	FALSE	97.7	51.7	143.6	108
Los Angele ...	non-reside ...	2020-03-16	FALSE	107.4	61.1	153.7	148
Los Angele ...	theft of v ...	2020-04-06	TRUE	295.5	226.8	364.2	404
Memphis, T ...	theft of v ...	2020-01-20	TRUE	58.7	28.5	88.9	107
Montgomery ...	non-reside ...	2020-03-23	FALSE	-0.1	0.0	10.6	12
Nashville, ...	serious as ...	2020-04-13	FALSE	44.9	4.5	85.4	NA
Phoenix, A ...	non-reside ...	2020-02-03	FALSE	55.0	33.3	76.7	40
Phoenix, A ...	serious as ...	2020-04-13	FALSE	54.0	37.7	70.4	NA
Tucson, AZ	theft of v ...	2020-02-03	FALSE	40.9	20.7	61.2	42
Tucson, AZ	theft of v ...	2020-02-24	FALSE	45.5	23.8	67.2	32
Washington ...	theft from ...	2020-04-13	FALSE	190.8	101.8	279.8	89

The field `estimated_crimes` shows the number of crimes forecast to occur during each week. The fields `estimate_lower_ci` and `estimate_upper_ci` show the limits of the 99% confidence interval around that forecast. Forecasts are provided for weeks from 20 January 2020 onwards, with these fields having `NA` values before that date. The field `actual_crimes` shows the number of crimes actually recorded by police in that week, for comparison against the forecast. Values of `actual_crimes` are `NA` for dates after 19 April 2020 (for which forecasts are nevertheless available) or where data were missing from the original source data.
