# Supplementary material for: Initial evidence on the relationship between the coronavirus pandemic and crime in the United States
Source: Crime Sci. 2020 May 18;9(1):6. doi: 10.1186/s40163-020-00117-6 (PMC7233195; doi:10.1186/s40163-020-00117-6)
Supplement: Supplementary file 2 — Additional file 2. SARIMA model co-efficients. [file 40163_2020_117_MOESM2_ESM.pdf]

Initial evidence on COVID-19 and crime in the United States: model results tables

Matthew P J Ashby, Jill Dando Institute of Security and Crime Science, University College London

15 May 2020

Contents

Introduction **1**

Serious assaults in public **3**

Serious assaults in residences **15**

Residential burglary **27**

Non-residential burglary **43**

Theft of vehicle **60**

Theft from vehicle **80**

References **97**

Introduction

The article 'Initial evidence on the relationship between the coronavirus pandemic and crime in the United States' – available at `https://osf.io/ef4dw/` – uses 93 seasonal auto-regressive integrated moving average (SARIMA) models to estimate the frequency of crime in 18 of the 100 largest US cities. For reasons of space, the models are summarised in the paper and presented in full form in this supplementary material.

Each model estimates the weekly frequency of a particular type of crime in a particular city. Each model contains the following variables:

- zero or more moving-average (MA) terms,
- zero or more auto-regressive (AR) terms,
- zero or more seasonal auto-regressive (SAR) terms,
- a linear trend term,
- 51 dummy term representing weeks of the year, and
- a dummy representing whether there is a US federal holiday in the week.

The number of MA, AR and SAR terms is chosen automatically using the algorithm outlined by Hyndman and Khandakar (2008), in which multiple models with different values for the SARIMA terms are estimated and the model which minimizes the Akaike information criterion (AIC) estimator of prediction error is chosen.

Table 1 of the article shows the mean absolute scaled error (MASE) of each model. The MASE, introduced by Hyndman and Koehler (2006), compares the within-sample error of each model to the error of a corresponding naïve forecasting model. MASE values greater than one indicate a naïve model would have lower error, while

values less than one indicate the chosen SARIMA model has a lower error. In all models used in this study, the MASE was lower than one. For many forecasting circumstances, it would be preferable to compare models by comparing out-of-sample forecasting error. In the present case no comparable out-of-sample data were available because the pandemic has occurred everywhere (precluding the use of data from other cities) and because data from after the beginning of the pandemic cannot be assumed to be comparable to those from before it began.

The data in the following tables is also available in CSV format at `https://osf.io/qfsd5/`

Serious assaults in public

Serious assaults in public in Atlanta, GA	estimate	SE	statistic	p value
intercept	16.45	1.39	11.84	<0.001
MA(1)	0.11	0.07	1.49	0.139
SAR(1)	–0.39	0.08	–4.85	<0.001
linear trend	–0.01	0.00	–2.82	0.005
week 2 of the year	–3.16	1.74	–1.82	0.071
week 3 of the year	–1.28	1.84	–0.70	0.488
week 4 of the year	–3.72	2.06	–1.81	0.072
week 5 of the year	–2.89	1.86	–1.55	0.122
week 6 of the year	–6.11	1.84	–3.32	0.001
week 7 of the year	–1.30	1.84	–0.71	0.480
week 8 of the year	–1.73	1.84	–0.94	0.351
week 9 of the year	–6.36	2.16	–2.95	0.004
week 10 of the year	–4.36	1.91	–2.28	0.024
week 11 of the year	–0.78	1.85	–0.42	0.675
week 12 of the year	–2.98	2.08	–1.43	0.154
week 13 of the year	–4.98	1.87	–2.66	0.009
week 14 of the year	–3.37	1.85	–1.82	0.071
week 15 of the year	0.53	1.85	0.29	0.775
week 16 of the year	–1.47	1.86	–0.79	0.431
week 17 of the year	–4.12	2.07	–1.99	0.048
week 18 of the year	–2.47	1.85	–1.34	0.183
week 19 of the year	–0.30	1.85	–0.16	0.872
week 20 of the year	–0.96	1.85	–0.52	0.603
week 21 of the year	–0.72	1.86	–0.39	0.700
week 22 of the year	–2.57	1.87	–1.37	0.172
week 23 of the year	–3.12	1.84	–1.69	0.092
week 24 of the year	–1.37	1.88	–0.73	0.468
week 25 of the year	–2.00	1.86	–1.07	0.284
week 26 of the year	2.73	1.84	1.48	0.140
week 27 of the year	0.13	1.84	0.07	0.944
week 28 of the year	3.09	1.84	1.68	0.095
week 29 of the year	4.01	1.85	2.17	0.031
week 30 of the year	0.58	1.84	0.32	0.753
week 31 of the year	0.66	2.17	0.31	0.760
week 32 of the year	3.22	1.84	1.75	0.083
week 33 of the year	3.81	1.84	2.07	0.040
week 34 of the year	0.08	1.84	0.04	0.966
week 35 of the year	3.16	1.85	1.71	0.089
week 36 of the year	–0.58	2.18	–0.27	0.789
week 37 of the year	3.77	1.84	2.05	0.042
week 38 of the year	–0.83	1.84	–0.45	0.653
week 39 of the year	0.34	1.84	0.19	0.852
week 40 of the year	–0.82	1.84	–0.45	0.657
week 41 of the year	–3.51	1.84	–1.90	0.059
week 42 of the year	0.28	1.84	0.15	0.878
week 43 of the year	0.38	1.84	0.20	0.839
week 44 of the year	3.05	1.84	1.65	0.100
week 45 of the year	–2.00	2.16	–0.93	0.356
week 46 of the year	0.76	1.84	0.41	0.680
week 47 of the year	4.62	1.85	2.50	0.014

Serious assaults in public in Atlanta, GA	estimate	SE	statistic	p value
week 48 of the year	0.71	1.85	0.38	0.702
week 49 of the year	1.87	1.84	1.02	0.310
week 50 of the year	–0.67	1.84	–0.37	0.715
week 51 of the year	1.30	1.84	0.71	0.481
week 52 of the year	–0.25	1.74	–0.15	0.884
holiday in week	1.67	1.14	1.46	0.146

Serious assaults in public in Austin, TX	estimate	SE	statistic	p value
intercept	66.57	3.92	16.99	<0.001
AR(1)	0.14	0.07	2.07	0.040
AR(2)	0.05	0.07	0.68	0.499
AR(3)	0.14	0.07	1.97	0.051
AR(4)	0.24	0.07	3.37	<0.001
SAR(1)	–0.35	0.09	–4.08	<0.001
linear trend	0.02	0.02	1.02	0.310
week 2 of the year	–4.89	4.14	–1.18	0.239
week 3 of the year	–9.44	4.34	–2.17	0.031
week 4 of the year	–13.98	4.64	–3.01	0.003
week 5 of the year	–9.39	3.95	–2.38	0.019
week 6 of the year	–15.80	4.38	–3.61	<0.001
week 7 of the year	–16.22	4.45	–3.64	<0.001
week 8 of the year	–19.57	4.41	–4.44	<0.001
week 9 of the year	–17.10	5.13	–3.34	0.001
week 10 of the year	–14.57	4.84	–3.01	0.003
week 11 of the year	–7.08	4.33	–1.64	0.104
week 12 of the year	–22.71	4.62	–4.92	<0.001
week 13 of the year	–12.28	4.64	–2.65	0.009
week 14 of the year	–5.40	4.63	–1.17	0.245
week 15 of the year	–11.86	4.64	–2.56	0.012
week 16 of the year	–10.46	4.67	–2.24	0.026
week 17 of the year	–11.93	5.12	–2.33	0.021
week 18 of the year	–2.43	4.68	–0.52	0.605
week 19 of the year	–2.88	4.69	–0.62	0.539
week 20 of the year	12.35	4.69	2.63	0.009
week 21 of the year	–3.47	4.74	–0.73	0.465
week 22 of the year	–2.03	4.76	–0.43	0.670
week 23 of the year	–3.27	4.70	–0.70	0.487
week 24 of the year	–4.79	4.77	–1.01	0.316
week 25 of the year	–1.93	4.73	–0.41	0.684
week 26 of the year	–1.74	4.70	–0.37	0.712
week 27 of the year	–1.08	4.70	–0.23	0.818
week 28 of the year	–4.13	4.70	–0.88	0.381
week 29 of the year	5.95	4.69	1.27	0.207
week 30 of the year	7.42	4.69	1.58	0.116
week 31 of the year	2.64	5.37	0.49	0.623
week 32 of the year	6.05	4.69	1.29	0.199
week 33 of the year	2.57	4.69	0.55	0.584
week 34 of the year	–2.32	4.68	–0.50	0.620
week 35 of the year	4.85	4.68	1.04	0.302
week 36 of the year	–0.87	5.35	–0.16	0.871
week 37 of the year	–2.23	4.65	–0.48	0.632

Serious assaults in public in Austin, TX	estimate	SE	statistic	p value
week 38 of the year	–4.92	4.64	–1.06	0.291
week 39 of the year	4.39	4.64	0.95	0.345
week 40 of the year	–5.14	4.62	–1.11	0.268
week 41 of the year	–3.73	4.61	–0.81	0.420
week 42 of the year	0.25	4.57	0.05	0.957
week 43 of the year	–6.84	4.57	–1.50	0.136
week 44 of the year	0.72	4.54	0.16	0.875
week 45 of the year	–1.05	5.16	–0.20	0.838
week 46 of the year	–1.53	4.42	–0.35	0.729
week 47 of the year	–1.38	4.48	–0.31	0.758
week 48 of the year	–6.53	4.40	–1.48	0.140
week 49 of the year	–0.22	3.92	–0.06	0.956
week 50 of the year	2.33	4.15	0.56	0.575
week 51 of the year	1.29	4.34	0.30	0.766
week 52 of the year	–13.31	4.15	–3.21	0.002
holiday in week	5.73	2.61	2.20	0.029

Serious assaults in public in Baltimore, MD	estimate	SE	statistic	p value
intercept	69.69	4.58	15.23	<0.001
AR(1)	0.48	0.31	1.54	0.125
MA(1)	–0.32	0.34	–0.96	0.340
SAR(1)	–0.36	0.08	–4.68	<0.001
linear trend	–0.04	0.01	–2.82	0.006
week 2 of the year	–5.72	5.44	–1.05	0.295
week 3 of the year	–1.89	5.72	–0.33	0.742
week 4 of the year	–10.24	6.44	–1.59	0.114
week 5 of the year	–14.41	5.96	–2.42	0.017
week 6 of the year	–1.37	5.94	–0.23	0.818
week 7 of the year	–10.23	5.96	–1.72	0.088
week 8 of the year	–3.47	5.97	–0.58	0.562
week 9 of the year	–8.60	6.81	–1.26	0.209
week 10 of the year	–17.79	6.30	–2.82	0.005
week 11 of the year	–20.25	5.63	–3.59	<0.001
week 12 of the year	–25.26	6.01	–4.21	<0.001
week 13 of the year	–5.21	6.02	–0.86	0.388
week 14 of the year	–15.32	6.00	–2.55	0.012
week 15 of the year	–23.46	6.00	–3.91	<0.001
week 16 of the year	–22.37	6.04	–3.70	<0.001
week 17 of the year	–9.94	6.56	–1.51	0.132
week 18 of the year	–28.05	6.00	–4.68	<0.001
week 19 of the year	–14.39	6.00	–2.40	0.018
week 20 of the year	–10.54	5.99	–1.76	0.081
week 21 of the year	–13.98	6.03	–2.32	0.022
week 22 of the year	–5.50	6.07	–0.91	0.366
week 23 of the year	–3.98	5.99	–0.66	0.508
week 24 of the year	10.85	6.07	1.79	0.076
week 25 of the year	2.51	6.03	0.42	0.677
week 26 of the year	4.83	5.98	0.81	0.421
week 27 of the year	5.26	5.99	0.88	0.381
week 28 of the year	–3.89	5.98	–0.65	0.516
week 29 of the year	14.43	5.98	2.41	0.017

Serious assaults in public in Baltimore, MD	estimate	SE	statistic	p value
week 30 of the year	3.42	6.00	0.57	0.570
week 31 of the year	9.78	6.87	1.42	0.157
week 32 of the year	20.34	5.98	3.40	<0.001
week 33 of the year	8.64	5.98	1.45	0.150
week 34 of the year	12.91	5.99	2.16	0.033
week 35 of the year	14.88	5.98	2.49	0.014
week 36 of the year	7.41	6.87	1.08	0.282
week 37 of the year	6.81	5.98	1.14	0.257
week 38 of the year	16.97	5.98	2.84	0.005
week 39 of the year	1.27	5.98	0.21	0.833
week 40 of the year	1.62	5.98	0.27	0.786
week 41 of the year	17.48	5.97	2.93	0.004
week 42 of the year	9.98	5.97	1.67	0.097
week 43 of the year	29.28	5.97	4.90	<0.001
week 44 of the year	14.23	5.98	2.38	0.019
week 45 of the year	–0.57	6.87	–0.08	0.934
week 46 of the year	3.76	5.97	0.63	0.530
week 47 of the year	17.86	5.96	3.00	0.003
week 48 of the year	2.46	5.95	0.41	0.680
week 49 of the year	10.75	5.92	1.82	0.071
week 50 of the year	16.71	5.85	2.86	0.005
week 51 of the year	0.95	5.72	0.17	0.868
week 52 of the year	2.14	5.44	0.39	0.695
holiday in week	–2.22	3.39	–0.65	0.514

Serious assaults in public in Chicago, IL	estimate	SE	statistic	p value
intercept	444.82	12.90	34.49	<0.001
SAR(1)	–0.46	0.07	–6.21	<0.001
linear trend	–0.25	0.03	–7.33	<0.001
week 2 of the year	–116.00	17.22	–6.74	<0.001
week 3 of the year	–149.22	17.20	–8.67	<0.001
week 4 of the year	–125.64	19.35	–6.49	<0.001
week 5 of the year	–131.95	17.38	–7.59	<0.001
week 6 of the year	–163.45	17.25	–9.48	<0.001
week 7 of the year	–161.18	17.26	–9.34	<0.001
week 8 of the year	–150.43	17.23	–8.73	<0.001
week 9 of the year	–153.40	20.55	–7.47	<0.001
week 10 of the year	–169.69	18.71	–9.07	<0.001
week 11 of the year	–185.33	16.26	–11.40	<0.001
week 12 of the year	–172.38	17.60	–9.80	<0.001
week 13 of the year	–162.31	17.45	–9.30	<0.001
week 14 of the year	–190.22	17.29	–11.00	<0.001
week 15 of the year	–204.07	17.27	–11.81	<0.001
week 16 of the year	–141.44	17.46	–8.10	<0.001
week 17 of the year	–174.52	19.39	–9.00	<0.001
week 18 of the year	–169.32	17.30	–9.79	<0.001
week 19 of the year	–140.90	17.25	–8.17	<0.001
week 20 of the year	–125.00	17.23	–7.25	<0.001
week 21 of the year	–111.72	17.38	–6.43	<0.001
week 22 of the year	–110.87	17.54	–6.32	<0.001
week 23 of the year	–110.66	17.25	–6.41	<0.001

Serious assaults in public in Chicago, IL	estimate	SE	statistic	p value
week 24 of the year	–59.77	17.56	–3.40	<0.001
week 25 of the year	–69.86	17.39	–4.02	<0.001
week 26 of the year	–97.68	17.24	–5.67	<0.001
week 27 of the year	–43.48	17.27	–2.52	0.013
week 28 of the year	–57.61	17.27	–3.34	0.001
week 29 of the year	31.92	17.29	1.85	0.067
week 30 of the year	10.77	17.21	0.63	0.532
week 31 of the year	25.99	20.49	1.27	0.207
week 32 of the year	8.06	17.28	0.47	0.642
week 33 of the year	30.96	17.24	1.80	0.074
week 34 of the year	17.01	17.22	0.99	0.325
week 35 of the year	57.35	17.23	3.33	0.001
week 36 of the year	73.60	20.49	3.59	<0.001
week 37 of the year	17.95	17.26	1.04	0.300
week 38 of the year	10.00	17.23	0.58	0.563
week 39 of the year	15.28	17.23	0.89	0.377
week 40 of the year	41.28	17.23	2.40	0.018
week 41 of the year	–14.90	17.22	–0.87	0.388
week 42 of the year	36.57	17.26	2.12	0.036
week 43 of the year	–16.86	17.22	–0.98	0.329
week 44 of the year	–15.35	17.23	–0.89	0.374
week 45 of the year	–38.44	20.48	–1.88	0.062
week 46 of the year	–32.44	17.23	–1.88	0.062
week 47 of the year	32.04	17.29	1.85	0.066
week 48 of the year	–40.40	17.30	–2.33	0.021
week 49 of the year	–52.78	17.31	–3.05	0.003
week 50 of the year	–47.08	17.21	–2.74	0.007
week 51 of the year	–26.58	17.20	–1.55	0.124
week 52 of the year	–57.83	17.21	–3.36	<0.001
holiday in week	9.11	11.20	0.81	0.417

Serious assaults in public in Dallas, TX	estimate	SE	statistic	p value
SAR(1)	–0.37	0.10	–3.70	<0.001
linear trend	0.08	0.01	6.72	<0.001
week 2 of the year	20.09	3.82	5.26	<0.001
week 3 of the year	28.23	3.86	7.31	<0.001
week 4 of the year	17.20	4.40	3.90	<0.001
week 5 of the year	24.18	3.90	6.20	<0.001
week 6 of the year	21.04	3.82	5.51	<0.001
week 7 of the year	13.77	3.82	3.61	<0.001
week 8 of the year	24.74	3.85	6.43	<0.001
week 9 of the year	17.43	4.08	4.27	<0.001
week 10 of the year	11.37	4.53	2.51	0.014
week 11 of the year	19.33	3.24	5.98	<0.001
week 12 of the year	17.76	3.63	4.89	<0.001
week 13 of the year	22.21	3.75	5.92	<0.001
week 14 of the year	26.07	3.63	7.18	<0.001
week 15 of the year	27.36	3.61	7.58	<0.001
week 16 of the year	21.50	3.61	5.96	<0.001
week 17 of the year	12.13	4.79	2.53	0.013
week 18 of the year	20.79	3.63	5.72	<0.001

Serious assaults in public in Dallas, TX	estimate	SE	statistic	p value
week 19 of the year	26.79	3.62	7.41	<0.001
week 20 of the year	27.30	3.62	7.54	<0.001
week 21 of the year	24.08	3.64	6.61	<0.001
week 22 of the year	22.39	3.85	5.82	<0.001
week 23 of the year	24.33	3.65	6.67	<0.001
week 24 of the year	31.28	3.76	8.33	<0.001
week 25 of the year	26.80	3.79	7.06	<0.001
week 26 of the year	21.88	3.64	6.01	<0.001
week 27 of the year	30.77	3.69	8.34	<0.001
week 28 of the year	32.17	3.66	8.78	<0.001
week 29 of the year	27.13	3.66	7.42	<0.001
week 30 of the year	35.87	3.69	9.72	<0.001
week 31 of the year	22.12	4.84	4.57	<0.001
week 32 of the year	45.08	3.68	12.27	<0.001
week 33 of the year	22.56	3.69	6.11	<0.001
week 34 of the year	29.85	3.71	8.05	<0.001
week 35 of the year	34.85	3.69	9.45	<0.001
week 36 of the year	28.96	4.88	5.93	<0.001
week 37 of the year	22.44	3.76	5.97	<0.001
week 38 of the year	32.77	3.72	8.81	<0.001
week 39 of the year	16.84	3.71	4.54	<0.001
week 40 of the year	30.28	3.74	8.09	<0.001
week 41 of the year	29.42	3.74	7.87	<0.001
week 42 of the year	26.35	3.71	7.09	<0.001
week 43 of the year	29.12	3.73	7.81	<0.001
week 44 of the year	31.86	3.77	8.46	<0.001
week 45 of the year	19.11	4.90	3.90	<0.001
week 46 of the year	29.15	3.80	7.67	<0.001
week 47 of the year	24.07	3.76	6.40	<0.001
week 48 of the year	23.59	3.77	6.25	<0.001
week 49 of the year	28.73	3.74	7.68	<0.001
week 50 of the year	22.39	3.76	5.95	<0.001
week 51 of the year	19.21	3.77	5.09	<0.001
week 52 of the year	24.69	3.79	6.51	<0.001
holiday in week	6.41	3.15	2.03	0.044

Serious assaults in public in Los Angeles, CA	estimate	SE	statistic	p value
intercept	211.66	9.46	22.37	<0.001
AR(1)	0.12	0.07	1.77	0.079
AR(2)	–0.06	0.07	–0.91	0.366
AR(3)	0.18	0.07	2.63	0.009
SAR(1)	–0.36	0.08	–4.54	<0.001
linear trend	0.06	0.03	2.16	0.033
week 2 of the year	5.14	11.65	0.44	0.660
week 3 of the year	–9.45	12.53	–0.75	0.452
week 4 of the year	–4.93	12.55	–0.39	0.695
week 5 of the year	–22.62	12.18	–1.86	0.065
week 6 of the year	–22.96	12.44	–1.85	0.067
week 7 of the year	–13.28	12.18	–1.09	0.277
week 8 of the year	–24.04	12.26	–1.96	0.052
week 9 of the year	–30.01	14.22	–2.11	0.036

Serious assaults in public in Los Angeles, CA	estimate	SE	statistic	p value
week 10 of the year	–25.27	13.08	–1.93	0.055
week 11 of the year	–37.56	11.63	–3.23	0.002
week 12 of the year	–37.73	12.46	–3.03	0.003
week 13 of the year	–23.70	12.46	–1.90	0.059
week 14 of the year	–26.61	12.36	–2.15	0.033
week 15 of the year	–39.28	12.39	–3.17	0.002
week 16 of the year	–32.44	12.52	–2.59	0.010
week 17 of the year	–33.98	13.69	–2.48	0.014
week 18 of the year	–35.50	12.39	–2.87	0.005
week 19 of the year	–31.11	12.40	–2.51	0.013
week 20 of the year	1.89	12.41	0.15	0.879
week 21 of the year	–2.14	12.53	–0.17	0.865
week 22 of the year	–2.42	12.60	–0.19	0.848
week 23 of the year	7.47	12.41	0.60	0.548
week 24 of the year	14.35	12.62	1.14	0.257
week 25 of the year	–6.79	12.48	–0.54	0.587
week 26 of the year	–25.38	12.36	–2.05	0.042
week 27 of the year	–9.49	12.38	–0.77	0.445
week 28 of the year	–16.83	12.36	–1.36	0.175
week 29 of the year	–1.08	12.36	–0.09	0.930
week 30 of the year	2.02	12.36	0.16	0.870
week 31 of the year	30.35	14.31	2.12	0.035
week 32 of the year	–5.59	12.35	–0.45	0.652
week 33 of the year	32.35	12.36	2.62	0.010
week 34 of the year	16.51	12.36	1.34	0.184
week 35 of the year	11.31	12.37	0.91	0.362
week 36 of the year	46.46	14.28	3.25	0.001
week 37 of the year	7.31	12.34	0.59	0.554
week 38 of the year	14.56	12.37	1.18	0.241
week 39 of the year	11.23	12.38	0.91	0.366
week 40 of the year	19.00	12.33	1.54	0.125
week 41 of the year	6.21	12.34	0.50	0.616
week 42 of the year	–1.29	12.38	–0.10	0.917
week 43 of the year	–6.59	12.33	–0.53	0.594
week 44 of the year	11.33	12.31	0.92	0.359
week 45 of the year	5.30	14.35	0.37	0.713
week 46 of the year	–8.71	12.27	–0.71	0.479
week 47 of the year	–5.66	12.24	–0.46	0.645
week 48 of the year	–15.38	12.40	–1.24	0.217
week 49 of the year	–4.55	12.06	–0.38	0.707
week 50 of the year	2.26	11.25	0.20	0.841
week 51 of the year	1.17	12.52	0.09	0.926
week 52 of the year	16.79	11.63	1.44	0.151
holiday in week	–11.06	7.19	–1.54	0.126

Serious assaults in public in Louisville, KY	estimate	SE	statistic	p value
intercept	10.57	1.23	8.57	<0.001
AR(1)	0.19	0.02	8.15	<0.001
AR(2)	0.08	0.02	3.35	0.001
SAR(1)	–0.27	0.02	–11.38	<0.001
linear trend	0.00	0.00	–0.15	0.883

Serious assaults in public in Louisville, KY	estimate	SE	statistic	p value
week 2 of the year	–4.04	1.44	–2.81	0.006
week 3 of the year	–0.94	1.51	–0.62	0.535
week 4 of the year	–1.44	1.71	–0.84	0.400
week 5 of the year	–3.30	1.61	–2.05	0.042
week 6 of the year	–2.48	1.60	–1.54	0.125
week 7 of the year	–1.15	1.61	–0.71	0.476
week 8 of the year	–2.49	1.61	–1.55	0.124
week 9 of the year	–1.21	1.80	–0.67	0.502
week 10 of the year	–2.54	1.67	–1.52	0.131
week 11 of the year	–4.53	1.52	–2.98	0.003
week 12 of the year	–0.59	1.61	–0.37	0.715
week 13 of the year	–4.42	1.62	–2.73	0.007
week 14 of the year	–0.32	1.61	–0.20	0.841
week 15 of the year	–5.88	1.62	–3.64	<0.001
week 16 of the year	–2.88	1.63	–1.77	0.079
week 17 of the year	1.26	1.75	0.72	0.471
week 18 of the year	–4.08	1.62	–2.53	0.013
week 19 of the year	–5.30	1.61	–3.28	0.001
week 20 of the year	–2.36	1.61	–1.46	0.146
week 21 of the year	–0.89	1.62	–0.55	0.585
week 22 of the year	–0.19	1.63	–0.11	0.910
week 23 of the year	–2.67	1.61	–1.66	0.100
week 24 of the year	0.44	1.63	0.27	0.790
week 25 of the year	–1.91	1.62	–1.18	0.241
week 26 of the year	–1.24	1.61	–0.77	0.443
week 27 of the year	–1.76	1.61	–1.09	0.276
week 28 of the year	–3.18	1.61	–1.97	0.051
week 29 of the year	–1.80	1.61	–1.11	0.267
week 30 of the year	–1.98	1.61	–1.23	0.222
week 31 of the year	0.02	1.82	0.01	0.992
week 32 of the year	–0.49	1.61	–0.30	0.763
week 33 of the year	–0.71	1.61	–0.44	0.661
week 34 of the year	–0.69	1.61	–0.43	0.668
week 35 of the year	–0.53	1.61	–0.33	0.741
week 36 of the year	0.23	1.82	0.13	0.898
week 37 of the year	–0.63	1.61	–0.39	0.694
week 38 of the year	–3.47	1.61	–2.16	0.033
week 39 of the year	–0.29	1.61	–0.18	0.857
week 40 of the year	2.81	1.61	1.74	0.083
week 41 of the year	–2.57	1.61	–1.60	0.113
week 42 of the year	–5.03	1.61	–3.12	0.002
week 43 of the year	0.95	1.61	0.59	0.554
week 44 of the year	–1.75	1.61	–1.09	0.279
week 45 of the year	–2.88	1.82	–1.58	0.115
week 46 of the year	0.07	1.61	0.04	0.965
week 47 of the year	1.21	1.61	0.76	0.451
week 48 of the year	–3.16	1.60	–1.97	0.050
week 49 of the year	–0.15	1.60	–0.09	0.925
week 50 of the year	–1.41	1.58	–0.89	0.374
week 51 of the year	–0.97	1.51	–0.64	0.523
week 52 of the year	–3.03	1.44	–2.11	0.037
holiday in week	–1.31	0.85	–1.54	0.125

Serious assaults in public in Montgomery County, MD	estimate	SE	statistic	p value
intercept	6.25	0.81	7.68	<0.001
MA(1)	0.16	0.08	1.96	0.052
SAR(1)	–0.39	0.08	–4.62	<0.001
linear trend	0.00	0.00	–0.03	0.980
week 2 of the year	–1.75	0.96	–1.82	0.071
week 3 of the year	–1.07	1.05	–1.02	0.309
week 4 of the year	–1.61	1.24	–1.29	0.198
week 5 of the year	–1.86	1.06	–1.76	0.081
week 6 of the year	–0.55	1.05	–0.52	0.603
week 7 of the year	–1.50	1.04	–1.44	0.153
week 8 of the year	–0.49	1.05	–0.47	0.641
week 9 of the year	–2.22	1.34	–1.66	0.099
week 10 of the year	1.69	1.33	1.27	0.207
week 11 of the year	–3.20	1.05	–3.05	0.003
week 12 of the year	–2.75	1.13	–2.43	0.016
week 13 of the year	–2.15	1.16	–1.85	0.066
week 14 of the year	–0.66	1.15	–0.57	0.569
week 15 of the year	–2.77	1.15	–2.41	0.017
week 16 of the year	–1.13	1.15	–0.99	0.326
week 17 of the year	–2.71	1.42	–1.91	0.058
week 18 of the year	–2.02	1.15	–1.76	0.081
week 19 of the year	–2.84	1.15	–2.47	0.015
week 20 of the year	–0.84	1.15	–0.73	0.468
week 21 of the year	–0.13	1.15	–0.11	0.910
week 22 of the year	–1.16	1.20	–0.96	0.337
week 23 of the year	–1.77	1.15	–1.54	0.126
week 24 of the year	0.54	1.18	0.46	0.646
week 25 of the year	–0.93	1.17	–0.79	0.428
week 26 of the year	–2.30	1.15	–1.99	0.048
week 27 of the year	–1.07	1.15	–0.93	0.355
week 28 of the year	1.87	1.15	1.63	0.105
week 29 of the year	–1.48	1.15	–1.28	0.202
week 30 of the year	–0.48	1.15	–0.41	0.680
week 31 of the year	–2.07	1.41	–1.47	0.144
week 32 of the year	1.99	1.15	1.73	0.086
week 33 of the year	–0.49	1.15	–0.43	0.671
week 34 of the year	0.17	1.15	0.15	0.880
week 35 of the year	–2.29	1.05	–2.19	0.030
week 36 of the year	–2.85	1.33	–2.14	0.034
week 37 of the year	0.22	1.05	0.21	0.836
week 38 of the year	–1.96	1.05	–1.87	0.064
week 39 of the year	–1.54	1.05	–1.47	0.144
week 40 of the year	–1.41	1.05	–1.35	0.181
week 41 of the year	–1.70	1.05	–1.62	0.107
week 42 of the year	–0.33	1.05	–0.31	0.755
week 43 of the year	–0.96	1.05	–0.91	0.363
week 44 of the year	–0.50	1.04	–0.48	0.632
week 45 of the year	0.48	1.33	0.36	0.720
week 46 of the year	0.84	1.04	0.80	0.425
week 47 of the year	0.25	1.05	0.24	0.808
week 48 of the year	–1.05	1.04	–1.00	0.319
week 49 of the year	–2.83	1.05	–2.70	0.008

Serious assaults in public in Montgomery County, MD	estimate	SE	statistic	p value
week 50 of the year	–0.66	1.04	–0.64	0.526
week 51 of the year	–1.83	1.05	–1.75	0.083
week 52 of the year	–0.49	0.96	–0.51	0.611
holiday in week	–0.06	0.82	–0.07	0.943

Serious assaults in public in Nashville, TN	estimate	SE	statistic	p value
AR(1)	0.31	0.07	4.71	<0.001
AR(2)	0.28	0.07	4.02	<0.001
AR(3)	0.35	0.07	5.09	<0.001
SAR(1)	–0.32	0.08	–4.05	<0.001
linear trend	0.46	0.09	5.08	<0.001
week 2 of the year	–6.53	6.98	–0.94	0.351
week 3 of the year	10.15	7.09	1.43	0.155
week 4 of the year	–4.56	7.50	–0.61	0.544
week 5 of the year	–1.20	8.01	–0.15	0.881
week 6 of the year	–5.73	8.28	–0.69	0.490
week 7 of the year	–13.40	8.58	–1.56	0.121
week 8 of the year	–1.38	9.00	–0.15	0.878
week 9 of the year	–11.03	10.18	–1.08	0.280
week 10 of the year	–15.75	10.17	–1.55	0.124
week 11 of the year	–17.64	9.77	–1.81	0.073
week 12 of the year	–22.06	10.28	–2.15	0.033
week 13 of the year	–20.12	10.56	–1.91	0.059
week 14 of the year	–25.22	10.76	–2.34	0.020
week 15 of the year	–22.13	10.97	–2.02	0.045
week 16 of the year	–24.49	11.24	–2.18	0.031
week 17 of the year	–26.44	11.82	–2.24	0.027
week 18 of the year	–20.78	11.58	–1.79	0.075
week 19 of the year	–15.80	11.73	–1.35	0.180
week 20 of the year	–13.02	11.87	–1.10	0.274
week 21 of the year	–19.30	12.00	–1.61	0.110
week 22 of the year	–16.34	12.11	–1.35	0.179
week 23 of the year	–9.36	12.14	–0.77	0.442
week 24 of the year	–6.88	12.21	–0.56	0.574
week 25 of the year	–16.61	12.22	–1.36	0.176
week 26 of the year	0.60	12.26	0.05	0.961
week 27 of the year	–5.26	12.24	–0.43	0.668
week 28 of the year	–6.76	12.22	–0.55	0.581
week 29 of the year	–1.20	12.18	–0.10	0.922
week 30 of the year	5.26	12.11	0.43	0.665
week 31 of the year	–2.02	12.71	–0.16	0.874
week 32 of the year	29.46	11.93	2.47	0.015
week 33 of the year	29.62	11.87	2.50	0.014
week 34 of the year	14.86	11.73	1.27	0.207
week 35 of the year	20.07	11.61	1.73	0.086
week 36 of the year	26.83	12.17	2.20	0.029
week 37 of the year	19.02	11.31	1.68	0.095
week 38 of the year	26.85	11.14	2.41	0.017
week 39 of the year	13.28	10.94	1.21	0.227
week 40 of the year	9.70	10.73	0.90	0.367
week 41 of the year	13.22	10.51	1.26	0.210

Serious assaults in public in Nashville, TN	estimate	SE	statistic	p value
week 42 of the year	10.78	10.26	1.05	0.295
week 43 of the year	6.14	9.97	0.62	0.539
week 44 of the year	6.23	9.70	0.64	0.522
week 45 of the year	20.44	10.22	2.00	0.047
week 46 of the year	8.95	9.09	0.98	0.326
week 47 of the year	23.90	8.55	2.79	0.006
week 48 of the year	9.80	8.30	1.18	0.240
week 49 of the year	4.06	7.97	0.51	0.611
week 50 of the year	–6.61	6.85	–0.97	0.336
week 51 of the year	18.14	7.09	2.56	0.011
week 52 of the year	–1.01	7.00	–0.14	0.886
holiday in week	5.12	3.96	1.29	0.198

Serious assaults in public in Phoenix, AZ	estimate	SE	statistic	p value
intercept	36.85	2.25	16.35	<0.001
SAR(1)	–0.38	0.08	–4.79	<0.001
linear trend	0.05	0.01	8.64	<0.001
week 2 of the year	5.88	3.01	1.95	0.053
week 3 of the year	2.97	3.01	0.99	0.325
week 4 of the year	–5.28	3.35	–1.57	0.117
week 5 of the year	–2.53	3.03	–0.83	0.406
week 6 of the year	0.95	3.01	0.32	0.752
week 7 of the year	–7.60	3.01	–2.53	0.013
week 8 of the year	0.54	3.01	0.18	0.857
week 9 of the year	–13.82	3.56	–3.88	<0.001
week 10 of the year	–7.53	3.26	–2.31	0.022
week 11 of the year	–9.87	2.84	–3.48	<0.001
week 12 of the year	–7.55	3.07	–2.46	0.015
week 13 of the year	–11.20	3.05	–3.67	<0.001
week 14 of the year	–4.14	3.01	–1.37	0.171
week 15 of the year	–0.96	3.02	–0.32	0.750
week 16 of the year	2.07	3.05	0.68	0.498
week 17 of the year	–10.46	3.38	–3.09	0.002
week 18 of the year	5.27	3.03	1.74	0.084
week 19 of the year	0.46	3.02	0.15	0.879
week 20 of the year	–0.43	3.02	–0.14	0.887
week 21 of the year	3.48	3.05	1.14	0.255
week 22 of the year	–1.24	3.07	–0.40	0.687
week 23 of the year	0.38	3.01	0.13	0.899
week 24 of the year	2.01	3.06	0.66	0.513
week 25 of the year	4.94	3.04	1.62	0.106
week 26 of the year	2.24	3.01	0.75	0.457
week 27 of the year	1.48	3.02	0.49	0.624
week 28 of the year	0.39	3.01	0.13	0.896
week 29 of the year	1.36	3.01	0.45	0.652
week 30 of the year	–3.34	3.01	–1.11	0.269
week 31 of the year	–4.68	3.54	–1.32	0.189
week 32 of the year	2.55	3.02	0.85	0.398
week 33 of the year	3.69	3.01	1.23	0.222
week 34 of the year	–3.54	3.01	–1.18	0.242
week 35 of the year	5.32	3.02	1.77	0.079

Serious assaults in public in Phoenix, AZ	estimate	SE	statistic	p value
week 36 of the year	–0.43	3.55	–0.12	0.903
week 37 of the year	–0.02	3.01	–0.01	0.993
week 38 of the year	0.40	3.01	0.13	0.896
week 39 of the year	–2.73	3.01	–0.91	0.365
week 40 of the year	–0.13	3.01	–0.04	0.965
week 41 of the year	–1.28	3.01	–0.43	0.670
week 42 of the year	4.98	3.01	1.65	0.101
week 43 of the year	1.89	3.01	0.63	0.531
week 44 of the year	4.43	3.02	1.47	0.144
week 45 of the year	–5.35	3.55	–1.51	0.133
week 46 of the year	0.94	3.01	0.31	0.756
week 47 of the year	–5.82	3.01	–1.93	0.055
week 48 of the year	–0.60	3.01	–0.20	0.842
week 49 of the year	0.53	3.01	0.18	0.861
week 50 of the year	–1.63	3.01	–0.54	0.589
week 51 of the year	5.67	3.01	1.89	0.061
week 52 of the year	0.41	3.01	0.14	0.892
holiday in week	4.56	1.87	2.43	0.016

Serious assaults in residences

Serious assaults in residences in Atlanta, GA	estimate	SE	statistic	p value
intercept	30.30	1.82	16.67	<0.001
AR(1)	0.64	0.20	3.24	0.001
MA(1)	–0.39	0.24	–1.61	0.109
SAR(1)	–0.36	0.08	–4.41	<0.001
linear trend	–0.03	0.01	–4.21	<0.001
week 2 of the year	–10.35	1.92	–5.38	<0.001
week 3 of the year	–10.36	2.05	–5.04	<0.001
week 4 of the year	–4.65	2.35	–1.98	0.050
week 5 of the year	–8.06	2.20	–3.66	<0.001
week 6 of the year	–5.39	2.22	–2.43	0.016
week 7 of the year	–6.49	2.24	–2.90	0.004
week 8 of the year	–14.20	2.25	–6.32	<0.001
week 9 of the year	–5.26	2.56	–2.06	0.041
week 10 of the year	–12.05	2.30	–5.25	<0.001
week 11 of the year	–6.93	2.28	–3.04	0.003
week 12 of the year	–14.40	2.47	–5.83	<0.001
week 13 of the year	–10.27	2.30	–4.46	<0.001
week 14 of the year	–11.97	2.29	–5.23	<0.001
week 15 of the year	–12.34	2.29	–5.39	<0.001
week 16 of the year	–14.81	2.30	–6.43	<0.001
week 17 of the year	–12.58	2.49	–5.06	<0.001
week 18 of the year	–12.84	2.29	–5.61	<0.001
week 19 of the year	–9.21	2.29	–4.03	<0.001
week 20 of the year	–8.76	2.29	–3.83	<0.001
week 21 of the year	–7.73	2.30	–3.36	<0.001
week 22 of the year	–3.73	2.31	–1.61	0.109
week 23 of the year	–6.33	2.28	–2.77	0.006
week 24 of the year	–6.38	2.31	–2.76	0.006
week 25 of the year	–7.50	2.30	–3.25	0.001
week 26 of the year	–8.24	2.28	–3.61	<0.001
week 27 of the year	–4.21	2.28	–1.85	0.067
week 28 of the year	–2.41	2.28	–1.06	0.292
week 29 of the year	–1.40	2.28	–0.62	0.539
week 30 of the year	–9.70	2.28	–4.25	<0.001
week 31 of the year	–2.61	2.59	–1.01	0.316
week 32 of the year	–6.68	2.28	–2.93	0.004
week 33 of the year	–4.57	2.28	–2.00	0.047
week 34 of the year	–6.02	2.28	–2.64	0.009
week 35 of the year	–4.80	2.28	–2.10	0.037
week 36 of the year	–7.50	2.59	–2.89	0.004
week 37 of the year	1.72	2.28	0.75	0.453
week 38 of the year	–2.66	2.28	–1.17	0.245
week 39 of the year	–7.51	2.28	–3.30	0.001
week 40 of the year	–6.25	2.27	–2.75	0.007
week 41 of the year	–2.53	2.27	–1.11	0.268
week 42 of the year	–7.56	2.27	–3.33	0.001
week 43 of the year	–1.82	2.27	–0.80	0.424
week 44 of the year	–2.87	2.27	–1.26	0.208
week 45 of the year	–2.49	2.58	–0.96	0.336
week 46 of the year	–11.75	2.25	–5.21	<0.001

Serious assaults in residences in Atlanta, GA	estimate	SE	statistic	p value
week 47 of the year	–8.32	2.24	–3.71	<0.001
week 48 of the year	–6.81	2.22	–3.07	0.003
week 49 of the year	–6.16	2.19	–2.81	0.006
week 50 of the year	–7.25	2.14	–3.39	<0.001
week 51 of the year	–5.89	2.06	–2.86	0.005
week 52 of the year	–11.42	1.92	–5.94	<0.001
holiday in week	0.42	1.24	0.34	0.733

Serious assaults in residences in Austin, TX	estimate	SE	statistic	p value
intercept	96.43	3.75	25.70	<0.001
AR(1)	0.81	NA	NA	NA
AR(2)	0.05	0.06	0.83	0.409
MA(1)	–0.84	NA	NA	NA
SAR(1)	–0.36	0.08	–4.76	<0.001
linear trend	–0.03	0.01	–2.45	0.015
week 2 of the year	–12.82	5.10	–2.52	0.013
week 3 of the year	–10.90	4.95	–2.20	0.029
week 4 of the year	–12.11	5.54	–2.19	0.030
week 5 of the year	–5.60	5.01	–1.12	0.266
week 6 of the year	–11.36	4.97	–2.28	0.024
week 7 of the year	–9.70	4.98	–1.95	0.053
week 8 of the year	–10.76	4.97	–2.17	0.032
week 9 of the year	–1.83	5.89	–0.31	0.756
week 10 of the year	–10.00	5.37	–1.86	0.065
week 11 of the year	–13.61	4.71	–2.89	0.004
week 12 of the year	–7.43	5.08	–1.46	0.146
week 13 of the year	–16.45	5.04	–3.26	0.001
week 14 of the year	–4.56	5.00	–0.91	0.364
week 15 of the year	–3.37	5.01	–0.67	0.502
week 16 of the year	–11.28	5.05	–2.23	0.027
week 17 of the year	–0.34	5.57	–0.06	0.951
week 18 of the year	–8.81	5.02	–1.76	0.081
week 19 of the year	–13.04	5.02	–2.60	0.010
week 20 of the year	–5.12	5.02	–1.02	0.309
week 21 of the year	–4.65	5.06	–0.92	0.359
week 22 of the year	–1.76	5.10	–0.35	0.731
week 23 of the year	–4.07	5.03	–0.81	0.419
week 24 of the year	–5.84	5.09	–1.15	0.254
week 25 of the year	1.19	5.06	0.24	0.815
week 26 of the year	–1.67	5.02	–0.33	0.740
week 27 of the year	–10.30	5.02	–2.05	0.042
week 28 of the year	–4.00	5.01	–0.80	0.426
week 29 of the year	–6.92	5.01	–1.38	0.170
week 30 of the year	1.20	5.01	0.24	0.811
week 31 of the year	8.10	5.90	1.37	0.172
week 32 of the year	4.54	5.01	0.91	0.366
week 33 of the year	–4.20	5.01	–0.84	0.404
week 34 of the year	2.83	5.01	0.56	0.573
week 35 of the year	–2.63	5.01	–0.52	0.601
week 36 of the year	19.82	5.89	3.37	<0.001
week 37 of the year	4.44	5.01	0.89	0.377

Serious assaults in residences in Austin, TX	estimate	SE	statistic	p value
week 38 of the year	7.09	5.01	1.42	0.159
week 39 of the year	3.96	5.00	0.79	0.430
week 40 of the year	1.33	5.00	0.27	0.791
week 41 of the year	–4.99	5.00	–1.00	0.319
week 42 of the year	1.66	5.00	0.33	0.740
week 43 of the year	5.05	5.00	1.01	0.314
week 44 of the year	0.56	4.99	0.11	0.911
week 45 of the year	–1.87	5.90	–0.32	0.752
week 46 of the year	9.09	4.99	1.82	0.070
week 47 of the year	0.67	4.98	0.13	0.893
week 48 of the year	–11.86	4.98	–2.38	0.018
week 49 of the year	–4.49	4.97	–0.90	0.368
week 50 of the year	–5.10	4.97	–1.03	0.306
week 51 of the year	–8.66	4.95	–1.75	0.082
week 52 of the year	–5.23	5.09	–1.03	0.306
holiday in week	–4.00	3.10	–1.29	0.199

Serious assaults in residences in Baltimore, MD	estimate	SE	statistic	p value
intercept	35.40	2.71	13.08	<0.001
AR(1)	0.15	NA	NA	NA
AR(2)	0.06	NA	NA	NA
SAR(1)	–0.30	0.01	–39.17	<0.001
linear trend	0.00	0.01	–0.57	0.570
week 2 of the year	–4.11	3.27	–1.26	0.210
week 3 of the year	–2.69	3.42	–0.79	0.433
week 4 of the year	–5.45	3.84	–1.42	0.159
week 5 of the year	1.50	3.57	0.42	0.675
week 6 of the year	–1.82	3.56	–0.51	0.610
week 7 of the year	0.35	3.56	0.10	0.923
week 8 of the year	0.45	3.56	0.13	0.901
week 9 of the year	–3.33	4.04	–0.82	0.411
week 10 of the year	–12.05	3.74	–3.22	0.002
week 11 of the year	–5.31	3.36	–1.58	0.116
week 12 of the year	–10.10	3.57	–2.83	0.005
week 13 of the year	–8.79	3.59	–2.45	0.015
week 14 of the year	–3.40	3.57	–0.95	0.343
week 15 of the year	–3.79	3.57	–1.06	0.291
week 16 of the year	–5.75	3.60	–1.60	0.112
week 17 of the year	–2.32	3.89	–0.60	0.551
week 18 of the year	–1.35	3.57	–0.38	0.705
week 19 of the year	–6.00	3.57	–1.68	0.095
week 20 of the year	2.22	3.57	0.62	0.535
week 21 of the year	3.26	3.60	0.91	0.365
week 22 of the year	3.89	3.61	1.08	0.284
week 23 of the year	–3.51	3.57	–0.98	0.327
week 24 of the year	6.62	3.61	1.83	0.069
week 25 of the year	1.11	3.60	0.31	0.758
week 26 of the year	6.29	3.57	1.76	0.080
week 27 of the year	7.16	3.57	2.01	0.046
week 28 of the year	0.18	3.57	0.05	0.960
week 29 of the year	–2.43	3.57	–0.68	0.497

Serious assaults in residences in Baltimore, MD	estimate	SE	statistic	p value
week 30 of the year	3.08	3.57	0.86	0.389
week 31 of the year	–6.62	4.07	–1.63	0.106
week 32 of the year	5.42	3.57	1.52	0.131
week 33 of the year	1.51	3.57	0.42	0.673
week 34 of the year	7.06	3.56	1.98	0.050
week 35 of the year	2.13	3.56	0.60	0.551
week 36 of the year	0.00	4.07	0.00	>0.999
week 37 of the year	–4.87	3.56	–1.37	0.174
week 38 of the year	3.06	3.56	0.86	0.393
week 39 of the year	–1.73	3.56	–0.49	0.628
week 40 of the year	–0.34	3.56	–0.09	0.925
week 41 of the year	–1.67	3.56	–0.47	0.639
week 42 of the year	–3.26	3.56	–0.91	0.362
week 43 of the year	0.63	3.56	0.18	0.859
week 44 of the year	0.69	3.56	0.19	0.847
week 45 of the year	–4.23	4.07	–1.04	0.300
week 46 of the year	6.50	3.56	1.83	0.070
week 47 of the year	0.13	3.56	0.04	0.970
week 48 of the year	3.50	3.56	0.98	0.326
week 49 of the year	1.65	3.55	0.46	0.643
week 50 of the year	3.93	3.52	1.12	0.266
week 51 of the year	–6.49	3.42	–1.90	0.059
week 52 of the year	–5.65	3.27	–1.73	0.085
holiday in week	3.14	1.96	1.60	0.112

Serious assaults in residences in Chicago, IL	estimate	SE	statistic	p value
intercept	186.84	7.20	25.95	<0.001
AR(1)	0.27	NA	NA	NA
MA(1)	–0.38	NA	NA	NA
MA(2)	–0.17	0.01	–20.97	<0.001
SAR(1)	–0.44	NA	NA	NA
linear trend	0.27	0.01	22.52	<0.001
week 2 of the year	–20.78	10.33	–2.01	0.046
week 3 of the year	–42.91	10.84	–3.96	<0.001
week 4 of the year	–31.11	11.47	–2.71	0.007
week 5 of the year	–22.79	10.14	–2.25	0.026
week 6 of the year	–14.89	9.99	–1.49	0.138
week 7 of the year	–27.38	9.97	–2.75	0.007
week 8 of the year	–42.79	9.97	–4.29	<0.001
week 9 of the year	–15.12	11.95	–1.27	0.207
week 10 of the year	–25.14	10.89	–2.31	0.022
week 11 of the year	–38.59	9.40	–4.11	<0.001
week 12 of the year	–25.54	10.22	–2.50	0.014
week 13 of the year	–36.84	10.08	–3.65	<0.001
week 14 of the year	–24.46	9.99	–2.45	0.015
week 15 of the year	–21.34	10.00	–2.13	0.034
week 16 of the year	–20.86	10.09	–2.07	0.040
week 17 of the year	–27.68	11.26	–2.46	0.015
week 18 of the year	–44.61	10.00	–4.46	<0.001
week 19 of the year	–38.45	9.99	–3.85	<0.001
week 20 of the year	–25.57	9.99	–2.56	0.011

Serious assaults in residences in Chicago, IL	estimate	SE	statistic	*p* value
week 21 of the year	–28.01	10.08	–2.78	0.006
week 22 of the year	–5.24	10.18	–0.52	0.607
week 23 of the year	–32.16	9.99	–3.22	0.002
week 24 of the year	–5.15	10.17	–0.51	0.613
week 25 of the year	–22.87	10.08	–2.27	0.025
week 26 of the year	1.70	9.99	0.17	0.865
week 27 of the year	1.35	9.99	0.14	0.892
week 28 of the year	–13.93	9.99	–1.40	0.165
week 29 of the year	–4.46	9.99	–0.45	0.656
week 30 of the year	15.26	9.98	1.53	0.128
week 31 of the year	41.56	11.93	3.49	<0.001
week 32 of the year	–4.58	9.99	–0.46	0.647
week 33 of the year	5.19	9.99	0.52	0.604
week 34 of the year	9.38	9.99	0.94	0.349
week 35 of the year	2.17	9.98	0.22	0.829
week 36 of the year	53.07	11.93	4.45	<0.001
week 37 of the year	11.16	9.98	1.12	0.265
week 38 of the year	12.15	9.98	1.22	0.226
week 39 of the year	0.15	9.99	0.02	0.988
week 40 of the year	–4.49	9.99	–0.45	0.654
week 41 of the year	10.01	9.99	1.00	0.318
week 42 of the year	13.64	9.99	1.37	0.174
week 43 of the year	–14.27	9.98	–1.43	0.155
week 44 of the year	–9.54	9.98	–0.96	0.341
week 45 of the year	–16.60	11.93	–1.39	0.166
week 46 of the year	1.07	9.99	0.11	0.915
week 47 of the year	–10.76	9.99	–1.08	0.283
week 48 of the year	–1.01	10.00	–0.10	0.920
week 49 of the year	–27.77	10.05	–2.76	0.006
week 50 of the year	–26.36	10.22	–2.58	0.011
week 51 of the year	–24.17	10.84	–2.23	0.027
week 52 of the year	–5.72	10.33	–0.55	0.581
holiday in week	2.35	6.53	0.36	0.719

Serious assaults in residences in Dallas, TX	estimate	SE	statistic	*p* value
MA(1)	0.22	0.07	2.93	0.004
SAR(1)	–0.59	0.08	–7.56	<0.001
linear trend	0.08	0.01	10.81	<0.001
week 2 of the year	5.38	2.03	2.65	0.009
week 3 of the year	7.71	2.12	3.64	<0.001
week 4 of the year	8.42	2.46	3.43	<0.001
week 5 of the year	3.12	2.18	1.43	0.156
week 6 of the year	8.03	2.13	3.77	<0.001
week 7 of the year	3.83	2.13	1.80	0.075
week 8 of the year	10.53	2.12	4.96	<0.001
week 9 of the year	13.10	2.26	5.79	<0.001
week 10 of the year	16.03	2.49	6.44	<0.001
week 11 of the year	4.12	1.79	2.30	0.023
week 12 of the year	8.89	1.99	4.48	<0.001
week 13 of the year	4.78	2.02	2.36	0.020
week 14 of the year	3.90	1.98	1.97	0.052

Serious assaults in residences in Dallas, TX	estimate	SE	statistic	p value
week 15 of the year	6.20	1.98	3.13	0.002
week 16 of the year	4.61	1.98	2.32	0.022
week 17 of the year	9.46	2.64	3.58	<0.001
week 18 of the year	8.75	1.99	4.39	<0.001
week 19 of the year	8.82	1.99	4.43	<0.001
week 20 of the year	5.25	2.00	2.63	0.010
week 21 of the year	6.34	2.00	3.17	0.002
week 22 of the year	7.39	2.14	3.45	<0.001
week 23 of the year	2.30	2.01	1.14	0.255
week 24 of the year	13.12	2.07	6.34	<0.001
week 25 of the year	7.30	2.07	3.53	<0.001
week 26 of the year	7.51	2.02	3.72	<0.001
week 27 of the year	6.71	2.02	3.31	0.001
week 28 of the year	7.63	2.03	3.76	<0.001
week 29 of the year	4.76	2.03	2.35	0.021
week 30 of the year	7.24	2.03	3.57	<0.001
week 31 of the year	16.45	2.67	6.16	<0.001
week 32 of the year	15.08	2.04	7.41	<0.001
week 33 of the year	8.61	2.04	4.22	<0.001
week 34 of the year	10.21	2.04	4.99	<0.001
week 35 of the year	16.83	2.08	8.10	<0.001
week 36 of the year	13.18	2.69	4.91	<0.001
week 37 of the year	10.68	2.05	5.21	<0.001
week 38 of the year	10.30	2.06	5.01	<0.001
week 39 of the year	9.30	2.06	4.52	<0.001
week 40 of the year	19.04	2.09	9.11	<0.001
week 41 of the year	10.05	2.07	4.85	<0.001
week 42 of the year	8.92	2.08	4.29	<0.001
week 43 of the year	4.44	2.08	2.14	0.035
week 44 of the year	8.63	2.08	4.16	<0.001
week 45 of the year	11.57	2.71	4.28	<0.001
week 46 of the year	7.09	2.09	3.40	<0.001
week 47 of the year	5.91	2.10	2.82	0.006
week 48 of the year	8.87	2.09	4.24	<0.001
week 49 of the year	7.74	2.10	3.69	<0.001
week 50 of the year	17.90	2.11	8.50	<0.001
week 51 of the year	7.86	2.10	3.74	<0.001
week 52 of the year	6.74	2.02	3.34	0.001
holiday in week	–0.80	1.76	–0.45	0.652

Serious assaults in residences in Los Angeles, CA	estimate	SE	statistic	p value
intercept	98.16	4.93	19.90	<0.001
AR(1)	0.83	0.12	6.68	<0.001
MA(1)	–0.77	0.14	–5.60	<0.001
SAR(1)	–0.34	0.08	–4.40	<0.001
linear trend	0.02	0.02	0.99	0.325
week 2 of the year	10.19	6.21	1.64	0.103
week 3 of the year	–4.69	6.25	–0.75	0.454
week 4 of the year	14.63	6.94	2.11	0.037
week 5 of the year	–6.56	6.35	–1.03	0.303
week 6 of the year	–5.51	6.34	–0.87	0.386

Serious assaults in residences in Los Angeles, CA	estimate	SE	statistic	p value
week 7 of the year	5.35	6.34	0.84	0.401
week 8 of the year	–0.50	6.36	–0.08	0.937
week 9 of the year	26.61	7.40	3.59	<0.001
week 10 of the year	18.39	6.81	2.70	0.008
week 11 of the year	–4.99	6.03	–0.83	0.409
week 12 of the year	3.88	6.47	0.60	0.549
week 13 of the year	–5.42	6.47	–0.84	0.404
week 14 of the year	4.52	6.43	0.70	0.483
week 15 of the year	–4.81	6.43	–0.75	0.456
week 16 of the year	3.29	6.49	0.51	0.613
week 17 of the year	3.22	7.10	0.45	0.651
week 18 of the year	–7.89	6.44	–1.23	0.222
week 19 of the year	–3.87	6.44	–0.60	0.549
week 20 of the year	8.76	6.44	1.36	0.176
week 21 of the year	2.95	6.49	0.45	0.650
week 22 of the year	26.96	6.53	4.13	<0.001
week 23 of the year	6.78	6.45	1.05	0.295
week 24 of the year	17.12	6.54	2.62	0.010
week 25 of the year	5.94	6.49	0.91	0.362
week 26 of the year	7.67	6.44	1.19	0.236
week 27 of the year	9.11	6.44	1.41	0.159
week 28 of the year	2.26	6.44	0.35	0.726
week 29 of the year	9.17	6.44	1.42	0.156
week 30 of the year	–2.35	6.44	–0.36	0.716
week 31 of the year	27.89	7.47	3.74	<0.001
week 32 of the year	15.34	6.43	2.38	0.018
week 33 of the year	25.24	6.43	3.93	<0.001
week 34 of the year	10.86	6.43	1.69	0.093
week 35 of the year	10.84	6.43	1.69	0.094
week 36 of the year	41.24	7.46	5.53	<0.001
week 37 of the year	26.07	6.44	4.05	<0.001
week 38 of the year	17.28	6.42	2.69	0.008
week 39 of the year	9.18	6.42	1.43	0.155
week 40 of the year	5.53	6.41	0.86	0.390
week 41 of the year	–0.81	6.40	–0.13	0.899
week 42 of the year	7.88	6.40	1.23	0.220
week 43 of the year	14.76	6.40	2.31	0.022
week 44 of the year	18.91	6.38	2.96	0.004
week 45 of the year	0.64	7.42	0.09	0.931
week 46 of the year	–2.43	6.36	–0.38	0.703
week 47 of the year	–0.80	6.35	–0.13	0.899
week 48 of the year	6.99	6.33	1.10	0.271
week 49 of the year	14.24	6.30	2.26	0.025
week 50 of the year	–2.20	6.28	–0.35	0.727
week 51 of the year	6.71	6.25	1.07	0.285
week 52 of the year	4.60	6.21	0.74	0.459
holiday in week	–4.98	3.78	–1.32	0.189

Serious assaults in residences in Louisville, KY	estimate	SE	statistic	p value
intercept	7.83	2.93	2.67	0.008
AR(1)	0.97	0.02	49.33	<0.001

Serious assaults in residences in Louisville, KY	estimate	SE	statistic	p value
MA(1)	–0.75	0.05	–13.77	<0.001
SAR(1)	–0.33	0.08	–4.24	<0.001
linear trend	0.05	0.02	2.19	0.030
week 2 of the year	–2.04	1.61	–1.27	0.207
week 3 of the year	1.20	1.65	0.73	0.469
week 4 of the year	–1.01	1.85	–0.55	0.584
week 5 of the year	–1.64	1.71	–0.95	0.341
week 6 of the year	–2.29	1.73	–1.32	0.187
week 7 of the year	1.81	1.75	1.04	0.302
week 8 of the year	–0.52	1.77	–0.29	0.771
week 9 of the year	–2.25	2.05	–1.10	0.272
week 10 of the year	–2.52	1.94	–1.30	0.195
week 11 of the year	–1.78	1.76	–1.01	0.312
week 12 of the year	–3.12	1.88	–1.66	0.098
week 13 of the year	–0.38	1.89	–0.20	0.842
week 14 of the year	0.44	1.89	0.23	0.815
week 15 of the year	–1.98	1.91	–1.04	0.301
week 16 of the year	–1.79	1.93	–0.93	0.354
week 17 of the year	–2.28	2.07	–1.10	0.274
week 18 of the year	–4.03	1.94	–2.08	0.039
week 19 of the year	–4.10	1.94	–2.11	0.037
week 20 of the year	–0.09	1.95	–0.05	0.963
week 21 of the year	0.39	1.97	0.20	0.844
week 22 of the year	–0.35	1.98	–0.18	0.859
week 23 of the year	–2.63	1.96	–1.34	0.182
week 24 of the year	1.17	1.99	0.59	0.558
week 25 of the year	–1.00	1.98	–0.51	0.614
week 26 of the year	0.47	1.97	0.24	0.811
week 27 of the year	–0.73	1.97	–0.37	0.711
week 28 of the year	–1.79	1.97	–0.91	0.365
week 29 of the year	0.72	1.97	0.37	0.713
week 30 of the year	2.17	1.96	1.10	0.272
week 31 of the year	–0.37	2.19	–0.17	0.866
week 32 of the year	–1.02	1.96	–0.52	0.604
week 33 of the year	–1.93	1.95	–0.99	0.324
week 34 of the year	0.80	1.94	0.41	0.680
week 35 of the year	–0.68	1.93	–0.35	0.725
week 36 of the year	1.71	2.16	0.79	0.430
week 37 of the year	–0.65	1.91	–0.34	0.736
week 38 of the year	–3.06	1.90	–1.61	0.110
week 39 of the year	–2.61	1.89	–1.38	0.170
week 40 of the year	–3.23	1.88	–1.72	0.087
week 41 of the year	–3.00	1.87	–1.61	0.110
week 42 of the year	–1.83	1.85	–0.99	0.324
week 43 of the year	0.26	1.83	0.14	0.888
week 44 of the year	–0.36	1.82	–0.20	0.842
week 45 of the year	5.25	2.05	2.56	0.011
week 46 of the year	3.97	1.77	2.24	0.027
week 47 of the year	2.06	1.75	1.18	0.240
week 48 of the year	2.52	1.73	1.46	0.147
week 49 of the year	0.40	1.70	0.23	0.815
week 50 of the year	–0.29	1.67	–0.17	0.862

Serious assaults in residences in Louisville, KY	estimate	SE	statistic	p value
week 51 of the year	5.88	1.65	3.57	<0.001
week 52 of the year	1.62	1.61	1.00	0.318
holiday in week	–1.52	0.99	–1.54	0.127

Serious assaults in residences in Montgomery County, MD	estimate	SE	statistic	p value
intercept	6.79	0.81	8.36	<0.001
SAR(1)	–0.49	0.08	–6.05	<0.001
linear trend	0.00	0.00	–0.27	0.785
week 2 of the year	2.29	1.06	2.16	0.033
week 3 of the year	–0.70	1.06	–0.66	0.508
week 4 of the year	1.70	1.30	1.31	0.193
week 5 of the year	–0.64	1.07	–0.60	0.550
week 6 of the year	–1.89	1.06	–1.79	0.076
week 7 of the year	–0.60	1.06	–0.56	0.573
week 8 of the year	0.80	1.06	0.76	0.451
week 9 of the year	3.16	1.41	2.23	0.027
week 10 of the year	1.75	1.41	1.24	0.218
week 11 of the year	–1.50	1.06	–1.41	0.160
week 12 of the year	–1.17	1.16	–1.01	0.314
week 13 of the year	–0.21	1.20	–0.18	0.858
week 14 of the year	0.15	1.17	0.13	0.900
week 15 of the year	–0.14	1.17	–0.12	0.902
week 16 of the year	–1.00	1.17	–0.85	0.394
week 17 of the year	–3.46	1.49	–2.31	0.022
week 18 of the year	–4.42	1.17	–3.79	<0.001
week 19 of the year	–1.13	1.17	–0.97	0.333
week 20 of the year	2.57	1.17	2.20	0.029
week 21 of the year	2.44	1.17	2.09	0.038
week 22 of the year	0.55	1.23	0.44	0.659
week 23 of the year	–0.27	1.17	–0.24	0.814
week 24 of the year	–1.49	1.20	–1.25	0.215
week 25 of the year	3.37	1.20	2.82	0.006
week 26 of the year	–0.42	1.17	–0.36	0.718
week 27 of the year	2.01	1.17	1.72	0.087
week 28 of the year	–1.13	1.17	–0.97	0.334
week 29 of the year	–1.28	1.17	–1.09	0.276
week 30 of the year	2.30	1.17	1.98	0.050
week 31 of the year	0.69	1.49	0.46	0.643
week 32 of the year	5.58	1.17	4.78	<0.001
week 33 of the year	0.16	1.17	0.14	0.891
week 34 of the year	–1.13	1.17	–0.97	0.336
week 35 of the year	–0.11	1.06	–0.10	0.919
week 36 of the year	1.24	1.41	0.88	0.383
week 37 of the year	–0.41	1.06	–0.39	0.698
week 38 of the year	1.88	1.07	1.76	0.080
week 39 of the year	2.19	1.06	2.07	0.041
week 40 of the year	0.78	1.06	0.74	0.461
week 41 of the year	1.49	1.06	1.41	0.161
week 42 of the year	–0.71	1.06	–0.67	0.506
week 43 of the year	–1.21	1.06	–1.14	0.256

Serious assaults in residences in Montgomery County, MD	estimate	SE	statistic	p value
week 44 of the year	–0.31	1.06	–0.29	0.771
week 45 of the year	–3.26	1.41	–2.31	0.023
week 46 of the year	1.19	1.06	1.12	0.264
week 47 of the year	0.09	1.06	0.09	0.931
week 48 of the year	1.09	1.06	1.02	0.309
week 49 of the year	–0.31	1.06	–0.29	0.770
week 50 of the year	0.59	1.06	0.56	0.576
week 51 of the year	–2.20	1.06	–2.08	0.039
week 52 of the year	1.10	1.06	1.03	0.303
holiday in week	0.75	0.94	0.80	0.424

Serious assaults in residences in Nashville, TN	estimate	SE	statistic	p value
intercept	43.74	9.76	4.48	<0.001
AR(1)	0.96	0.02	40.44	<0.001
MA(1)	–0.72	0.08	–9.26	<0.001
MA(2)	0.15	0.07	2.00	0.048
SAR(1)	–0.37	0.08	–4.74	<0.001
linear trend	0.13	0.07	1.84	0.067
week 2 of the year	0.48	4.65	0.10	0.918
week 3 of the year	–12.41	4.46	–2.79	0.006
week 4 of the year	–7.06	5.23	–1.35	0.179
week 5 of the year	–6.60	4.96	–1.33	0.185
week 6 of the year	–15.15	5.10	–2.97	0.003
week 7 of the year	–5.25	5.27	–0.99	0.321
week 8 of the year	–6.65	5.43	–1.23	0.222
week 9 of the year	6.25	6.30	0.99	0.323
week 10 of the year	–9.43	6.12	–1.54	0.126
week 11 of the year	–19.30	5.66	–3.41	<0.001
week 12 of the year	–23.45	6.05	–3.87	<0.001
week 13 of the year	–20.80	6.11	–3.41	<0.001
week 14 of the year	–18.32	6.16	–2.97	0.003
week 15 of the year	–19.33	6.25	–3.09	0.002
week 16 of the year	–22.76	6.36	–3.58	<0.001
week 17 of the year	–20.72	6.82	–3.04	0.003
week 18 of the year	–22.28	6.47	–3.44	<0.001
week 19 of the year	–26.16	6.52	–4.01	<0.001
week 20 of the year	–20.53	6.57	–3.12	0.002
week 21 of the year	–14.83	6.64	–2.23	0.027
week 22 of the year	–23.83	6.71	–3.55	<0.001
week 23 of the year	–14.24	6.67	–2.14	0.034
week 24 of the year	–21.14	6.76	–3.13	0.002
week 25 of the year	–21.23	6.74	–3.15	0.002
week 26 of the year	–14.87	6.72	–2.21	0.028
week 27 of the year	–20.76	6.71	–3.09	0.002
week 28 of the year	–13.41	6.70	–2.00	0.047
week 29 of the year	–16.42	6.70	–2.45	0.015
week 30 of the year	1.33	6.70	0.20	0.843
week 31 of the year	7.23	7.29	0.99	0.323
week 32 of the year	1.67	6.62	0.25	0.801
week 33 of the year	5.73	6.58	0.87	0.385

Serious assaults in residences in Nashville, TN	estimate	SE	statistic	p value
week 34 of the year	4.10	6.54	0.63	0.531
week 35 of the year	–3.21	6.49	–0.49	0.622
week 36 of the year	–5.51	7.07	–0.78	0.437
week 37 of the year	–1.63	6.38	–0.26	0.799
week 38 of the year	14.15	6.31	2.24	0.026
week 39 of the year	6.83	6.23	1.10	0.275
week 40 of the year	20.04	6.15	3.26	0.001
week 41 of the year	0.81	6.06	0.13	0.893
week 42 of the year	–15.41	5.96	–2.59	0.011
week 43 of the year	16.22	5.85	2.77	0.006
week 44 of the year	4.33	5.72	0.76	0.451
week 45 of the year	–9.24	6.32	–1.46	0.146
week 46 of the year	0.61	5.44	0.11	0.911
week 47 of the year	–5.89	5.28	–1.12	0.266
week 48 of the year	–3.72	5.11	–0.73	0.468
week 49 of the year	6.24	4.92	1.27	0.206
week 50 of the year	–0.03	4.70	–0.01	0.994
week 51 of the year	–8.72	4.46	–1.96	0.052
week 52 of the year	–8.84	4.65	–1.90	0.059
holiday in week	2.88	2.90	0.99	0.322

Serious assaults in residences in Phoenix, AZ	estimate	SE	statistic	p value
intercept	38.43	3.15	12.21	<0.001
AR(1)	0.95	0.03	28.10	<0.001
MA(1)	–0.82	0.06	–13.50	<0.001
SAR(1)	–0.43	0.08	–5.68	<0.001
linear trend	0.09	0.02	5.45	<0.001
week 2 of the year	3.70	3.02	1.23	0.222
week 3 of the year	–8.55	3.02	–2.83	0.005
week 4 of the year	3.46	3.42	1.01	0.312
week 5 of the year	–3.25	3.09	–1.05	0.295
week 6 of the year	–11.85	3.09	–3.84	<0.001
week 7 of the year	3.96	3.11	1.27	0.205
week 8 of the year	–5.26	3.12	–1.69	0.094
week 9 of the year	0.43	3.70	0.12	0.907
week 10 of the year	–5.78	3.43	–1.69	0.094
week 11 of the year	–3.07	3.01	–1.02	0.309
week 12 of the year	–6.08	3.25	–1.87	0.063
week 13 of the year	–4.90	3.24	–1.51	0.133
week 14 of the year	0.48	3.23	0.15	0.883
week 15 of the year	0.44	3.24	0.14	0.892
week 16 of the year	–9.09	3.27	–2.78	0.006
week 17 of the year	–4.27	3.60	–1.19	0.237
week 18 of the year	3.46	3.26	1.06	0.290
week 19 of the year	–4.90	3.27	–1.50	0.136
week 20 of the year	3.21	3.27	0.98	0.328
week 21 of the year	4.42	3.29	1.34	0.182
week 22 of the year	10.59	3.32	3.19	0.002
week 23 of the year	–0.37	3.28	–0.11	0.911
week 24 of the year	6.41	3.33	1.92	0.056
week 25 of the year	–2.67	3.30	–0.81	0.420

Serious assaults in residences in Phoenix, AZ	estimate	SE	statistic	p value
week 26 of the year	–0.97	3.28	–0.30	0.767
week 27 of the year	5.29	3.28	1.61	0.109
week 28 of the year	2.16	3.27	0.66	0.510
week 29 of the year	7.15	3.28	2.18	0.031
week 30 of the year	4.00	3.27	1.22	0.223
week 31 of the year	–0.51	3.79	–0.13	0.894
week 32 of the year	1.28	3.26	0.39	0.695
week 33 of the year	2.45	3.26	0.75	0.453
week 34 of the year	2.95	3.26	0.90	0.367
week 35 of the year	5.05	3.25	1.56	0.122
week 36 of the year	3.52	3.78	0.93	0.354
week 37 of the year	6.98	3.23	2.16	0.032
week 38 of the year	0.71	3.22	0.22	0.827
week 39 of the year	5.58	3.21	1.74	0.085
week 40 of the year	1.39	3.20	0.43	0.665
week 41 of the year	4.27	3.19	1.34	0.184
week 42 of the year	3.64	3.18	1.15	0.254
week 43 of the year	9.92	3.17	3.13	0.002
week 44 of the year	8.71	3.15	2.76	0.006
week 45 of the year	8.73	3.68	2.37	0.019
week 46 of the year	–1.51	3.12	–0.48	0.629
week 47 of the year	2.18	3.11	0.70	0.483
week 48 of the year	7.76	3.09	2.51	0.013
week 49 of the year	0.37	3.07	0.12	0.905
week 50 of the year	–3.11	3.05	–1.02	0.308
week 51 of the year	3.34	3.02	1.11	0.270
week 52 of the year	4.04	3.00	1.35	0.180
holiday in week	1.26	1.94	0.65	0.516

Residential burglary

Residential burglary in Atlanta, GA	estimate	SE	statistic	p value
intercept	70.31	4.23	16.63	<0.001
AR(1)	0.83	0.16	5.25	<0.001
MA(1)	–0.57	0.18	–3.23	0.002
MA(2)	0.00	0.11	–0.03	0.977
SAR(1)	–0.34	0.09	–3.96	<0.001
linear trend	–0.18	0.02	–8.70	<0.001
week 2 of the year	–3.10	3.61	–0.86	0.392
week 3 of the year	6.33	3.78	1.67	0.097
week 4 of the year	5.10	4.33	1.18	0.241
week 5 of the year	8.00	4.05	1.98	0.050
week 6 of the year	1.05	4.11	0.26	0.798
week 7 of the year	3.93	4.20	0.94	0.350
week 8 of the year	21.62	4.27	5.06	<0.001
week 9 of the year	1.77	4.94	0.36	0.721
week 10 of the year	–8.55	4.48	–1.91	0.058
week 11 of the year	–3.39	4.46	–0.76	0.448
week 12 of the year	–8.91	4.87	–1.83	0.069
week 13 of the year	–0.59	4.58	–0.13	0.897
week 14 of the year	–7.81	4.58	–1.70	0.090
week 15 of the year	–4.97	4.59	–1.08	0.281
week 16 of the year	–2.41	4.65	–0.52	0.606
week 17 of the year	–13.81	5.05	–2.73	0.007
week 18 of the year	–15.89	4.64	–3.42	<0.001
week 19 of the year	–10.87	4.64	–2.34	0.020
week 20 of the year	–14.85	4.63	–3.20	0.002
week 21 of the year	–16.04	4.68	–3.43	<0.001
week 22 of the year	–7.35	4.70	–1.56	0.120
week 23 of the year	–4.38	4.63	–0.95	0.346
week 24 of the year	–10.98	4.68	–2.35	0.020
week 25 of the year	–4.79	4.65	–1.03	0.304
week 26 of the year	–3.05	4.60	–0.66	0.508
week 27 of the year	–1.79	4.59	–0.39	0.698
week 28 of the year	–4.05	4.61	–0.88	0.380
week 29 of the year	–3.98	4.58	–0.87	0.386
week 30 of the year	0.33	4.59	0.07	0.943
week 31 of the year	–3.76	5.20	–0.72	0.471
week 32 of the year	–2.22	4.57	–0.49	0.628
week 33 of the year	–7.66	4.55	–1.69	0.094
week 34 of the year	–3.41	4.54	–0.75	0.454
week 35 of the year	–2.73	4.54	–0.60	0.548
week 36 of the year	5.53	5.15	1.07	0.285
week 37 of the year	–1.43	4.51	–0.32	0.752
week 38 of the year	8.91	4.49	1.98	0.049
week 39 of the year	–1.92	4.49	–0.43	0.670
week 40 of the year	–2.45	4.47	–0.55	0.586
week 41 of the year	–0.23	4.43	–0.05	0.959
week 42 of the year	3.91	4.40	0.89	0.375
week 43 of the year	–0.27	4.37	–0.06	0.951
week 44 of the year	3.95	4.33	0.91	0.363
week 45 of the year	–1.87	4.92	–0.38	0.705

Residential burglary in Atlanta, GA	estimate	SE	statistic	p value
week 46 of the year	0.32	4.25	0.08	0.939
week 47 of the year	–0.39	4.18	–0.09	0.926
week 48 of the year	2.41	4.11	0.59	0.559
week 49 of the year	2.40	4.02	0.60	0.552
week 50 of the year	–8.12	3.93	–2.07	0.041
week 51 of the year	2.26	3.79	0.60	0.551
week 52 of the year	2.52	3.61	0.70	0.486
holiday in week	–1.78	2.35	–0.76	0.451

Residential burglary in Austin, TX	estimate	SE	statistic	p value
intercept	77.65	4.13	18.80	<0.001
AR(1)	0.19	0.07	2.80	0.006
AR(2)	0.23	0.07	3.31	0.001
AR(3)	–0.05	0.07	–0.72	0.474
AR(4)	0.17	0.07	2.43	0.016
AR(5)	0.19	0.07	2.84	0.005
SAR(1)	–0.45	0.08	–5.93	<0.001
linear trend	–0.15	0.02	–6.30	<0.001
week 2 of the year	–8.45	3.54	–2.39	0.018
week 3 of the year	5.67	3.47	1.63	0.105
week 4 of the year	–1.57	4.23	–0.37	0.711
week 5 of the year	10.63	3.57	2.98	0.003
week 6 of the year	–2.64	3.53	–0.75	0.456
week 7 of the year	8.51	3.76	2.26	0.025
week 8 of the year	0.41	3.87	0.11	0.915
week 9 of the year	–3.84	4.51	–0.85	0.396
week 10 of the year	–10.17	4.21	–2.41	0.017
week 11 of the year	–8.38	3.80	–2.20	0.029
week 12 of the year	–2.47	4.08	–0.60	0.546
week 13 of the year	–7.81	4.13	–1.89	0.060
week 14 of the year	–9.06	4.14	–2.19	0.030
week 15 of the year	–15.44	4.16	–3.71	<0.001
week 16 of the year	–10.06	4.19	–2.40	0.018
week 17 of the year	–12.96	4.53	–2.86	0.005
week 18 of the year	–16.74	4.24	–3.95	<0.001
week 19 of the year	–14.27	4.26	–3.35	0.001
week 20 of the year	–10.89	4.26	–2.55	0.012
week 21 of the year	–13.66	4.30	–3.18	0.002
week 22 of the year	–15.11	4.33	–3.49	<0.001
week 23 of the year	–15.82	4.29	–3.69	<0.001
week 24 of the year	–16.90	4.35	–3.89	<0.001
week 25 of the year	–19.02	4.32	–4.40	<0.001
week 26 of the year	–8.82	4.30	–2.05	0.042
week 27 of the year	–8.54	4.30	–1.99	0.049
week 28 of the year	–11.59	4.30	–2.70	0.008
week 29 of the year	–12.36	4.29	–2.88	0.005
week 30 of the year	–10.26	4.28	–2.40	0.018
week 31 of the year	–2.74	4.82	–0.57	0.571
week 32 of the year	–18.59	4.27	–4.35	<0.001
week 33 of the year	–17.31	4.25	–4.07	<0.001
week 34 of the year	–8.44	4.24	–1.99	0.048

Residential burglary in Austin, TX	estimate	SE	statistic	p value
week 35 of the year	–11.64	4.23	–2.75	0.007
week 36 of the year	–8.35	4.77	–1.75	0.082
week 37 of the year	–4.36	4.19	–1.04	0.299
week 38 of the year	–17.16	4.16	–4.12	<0.001
week 39 of the year	–3.31	4.14	–0.80	0.426
week 40 of the year	–12.98	4.13	–3.14	0.002
week 41 of the year	–5.93	4.10	–1.45	0.150
week 42 of the year	–10.30	4.04	–2.55	0.012
week 43 of the year	–1.20	3.98	–0.30	0.763
week 44 of the year	–2.21	3.94	–0.56	0.575
week 45 of the year	–2.66	4.55	–0.59	0.559
week 46 of the year	–13.40	3.87	–3.46	<0.001
week 47 of the year	–6.98	3.77	–1.85	0.066
week 48 of the year	–3.45	3.54	–0.97	0.331
week 49 of the year	–4.11	3.55	–1.16	0.249
week 50 of the year	–10.28	3.85	–2.67	0.008
week 51 of the year	–3.40	3.46	–0.98	0.327
week 52 of the year	0.04	3.54	0.01	0.990
holiday in week	–6.38	2.22	–2.88	0.005

Residential burglary in Baltimore, MD	estimate	SE	statistic	p value
intercept	122.06	5.98	20.40	<0.001
AR(1)	0.51	0.07	7.55	<0.001
AR(2)	0.02	0.07	0.32	0.748
SAR(1)	–0.38	0.08	–4.98	<0.001
linear trend	–0.25	0.02	–10.16	<0.001
week 2 of the year	–0.36	5.04	–0.07	0.944
week 3 of the year	–0.93	6.16	–0.15	0.881
week 4 of the year	–9.29	7.13	–1.30	0.195
week 5 of the year	–5.14	7.00	–0.73	0.464
week 6 of the year	–2.63	7.12	–0.37	0.713
week 7 of the year	0.86	7.19	0.12	0.905
week 8 of the year	2.25	7.22	0.31	0.755
week 9 of the year	–12.62	7.73	–1.63	0.105
week 10 of the year	–28.37	7.36	–3.85	<0.001
week 11 of the year	–34.21	6.89	–4.96	<0.001
week 12 of the year	–30.49	7.15	–4.27	<0.001
week 13 of the year	–12.72	7.24	–1.76	0.081
week 14 of the year	–16.48	7.32	–2.25	0.026
week 15 of the year	–27.97	7.34	–3.81	<0.001
week 16 of the year	–18.49	7.38	–2.51	0.013
week 17 of the year	–21.18	7.75	–2.73	0.007
week 18 of the year	–33.32	7.35	–4.53	<0.001
week 19 of the year	–38.33	7.35	–5.22	<0.001
week 20 of the year	–36.20	7.35	–4.92	<0.001
week 21 of the year	–30.55	7.37	–4.14	<0.001
week 22 of the year	–31.13	7.40	–4.21	<0.001
week 23 of the year	–29.45	7.34	–4.01	<0.001
week 24 of the year	–18.14	7.39	–2.45	0.015
week 25 of the year	–21.12	7.36	–2.87	0.005
week 26 of the year	–10.87	7.33	–1.48	0.140

Residential burglary in Baltimore, MD	estimate	SE	statistic	p value
week 27 of the year	–22.50	7.33	–3.07	0.003
week 28 of the year	–11.37	7.33	–1.55	0.123
week 29 of the year	–4.03	7.32	–0.55	0.582
week 30 of the year	–20.10	7.32	–2.75	0.007
week 31 of the year	–10.29	7.97	–1.29	0.199
week 32 of the year	–7.40	7.32	–1.01	0.313
week 33 of the year	–17.80	7.33	–2.43	0.016
week 34 of the year	–27.14	7.32	–3.71	<0.001
week 35 of the year	–20.21	7.31	–2.76	0.006
week 36 of the year	–17.70	7.95	–2.23	0.027
week 37 of the year	–21.70	7.31	–2.97	0.003
week 38 of the year	–16.32	7.31	–2.23	0.027
week 39 of the year	–5.52	7.31	–0.76	0.451
week 40 of the year	–9.11	7.31	–1.25	0.214
week 41 of the year	–13.53	7.30	–1.85	0.066
week 42 of the year	–0.36	7.30	–0.05	0.961
week 43 of the year	–6.40	7.29	–0.88	0.381
week 44 of the year	–11.03	7.29	–1.51	0.132
week 45 of the year	–16.11	7.92	–2.04	0.044
week 46 of the year	–2.09	7.25	–0.29	0.774
week 47 of the year	2.25	7.20	0.31	0.755
week 48 of the year	1.35	7.12	0.19	0.850
week 49 of the year	–1.12	6.97	–0.16	0.873
week 50 of the year	11.96	6.70	1.79	0.076
week 51 of the year	12.40	6.16	2.01	0.046
week 52 of the year	–0.80	5.04	–0.16	0.874
holiday in week	–5.95	3.12	–1.91	0.058

Residential burglary in Boston, MA	estimate	SE	statistic	p value
intercept	31.95	2.42	13.19	<0.001
AR(1)	0.23	0.07	3.45	<0.001
AR(2)	0.24	0.07	3.60	<0.001
SAR(1)	–0.42	0.07	–5.60	<0.001
linear trend	–0.07	0.01	–6.81	<0.001
week 2 of the year	–2.18	2.49	–0.88	0.383
week 3 of the year	3.88	2.47	1.57	0.119
week 4 of the year	3.68	3.02	1.22	0.225
week 5 of the year	2.58	2.83	0.91	0.363
week 6 of the year	0.83	2.89	0.29	0.775
week 7 of the year	0.32	2.91	0.11	0.914
week 8 of the year	10.59	2.93	3.62	<0.001
week 9 of the year	2.42	3.30	0.73	0.465
week 10 of the year	3.48	3.09	1.13	0.262
week 11 of the year	1.72	2.80	0.62	0.539
week 12 of the year	6.41	2.97	2.16	0.032
week 13 of the year	2.64	2.98	0.89	0.376
week 14 of the year	4.04	2.98	1.36	0.177
week 15 of the year	1.25	3.01	0.42	0.679
week 16 of the year	6.14	3.01	2.04	0.043
week 17 of the year	7.78	3.24	2.40	0.018
week 18 of the year	1.05	3.00	0.35	0.728

Residential burglary in Boston, MA	estimate	SE	statistic	p value
week 19 of the year	–0.24	3.00	–0.08	0.935
week 20 of the year	–2.44	3.00	–0.82	0.416
week 21 of the year	0.74	3.01	0.25	0.806
week 22 of the year	3.44	3.03	1.13	0.258
week 23 of the year	2.04	3.00	0.68	0.498
week 24 of the year	1.18	3.03	0.39	0.697
week 25 of the year	–4.85	3.01	–1.61	0.109
week 26 of the year	0.57	2.99	0.19	0.849
week 27 of the year	–5.57	2.99	–1.86	0.064
week 28 of the year	0.70	2.99	0.24	0.814
week 29 of the year	2.33	3.00	0.78	0.438
week 30 of the year	8.98	2.99	3.00	0.003
week 31 of the year	4.98	3.37	1.48	0.142
week 32 of the year	6.70	2.99	2.24	0.026
week 33 of the year	9.84	2.99	3.29	0.001
week 34 of the year	4.23	2.99	1.41	0.159
week 35 of the year	1.40	2.98	0.47	0.640
week 36 of the year	5.76	3.37	1.71	0.090
week 37 of the year	4.88	2.99	1.63	0.104
week 38 of the year	2.24	2.98	0.75	0.455
week 39 of the year	6.56	2.99	2.20	0.029
week 40 of the year	3.83	2.98	1.29	0.201
week 41 of the year	7.25	2.99	2.43	0.016
week 42 of the year	3.77	2.98	1.27	0.207
week 43 of the year	0.70	2.97	0.24	0.813
week 44 of the year	6.34	2.97	2.14	0.034
week 45 of the year	6.00	3.35	1.79	0.075
week 46 of the year	6.72	2.95	2.28	0.024
week 47 of the year	8.39	2.92	2.87	0.005
week 48 of the year	9.94	2.89	3.44	<0.001
week 49 of the year	7.70	2.81	2.74	0.007
week 50 of the year	1.57	2.76	0.57	0.569
week 51 of the year	11.19	2.48	4.52	<0.001
week 52 of the year	4.54	2.49	1.82	0.070
holiday in week	–3.94	1.57	–2.51	0.013

Residential burglary in Chicago, IL	estimate	SE	statistic	p value
intercept	513.07	18.99	27.02	<0.001
AR(1)	1.18	0.14	8.18	<0.001
AR(2)	–0.24	0.12	–2.05	0.042
AR(3)	–0.10	0.11	–0.97	0.336
AR(4)	0.10	0.07	1.40	0.165
MA(1)	–0.76	0.13	–5.92	<0.001
SAR(1)	–0.34	0.07	–4.55	<0.001
linear trend	–0.94	0.10	–9.25	<0.001
week 2 of the year	–7.57	15.19	–0.50	0.619
week 3 of the year	–13.57	17.66	–0.77	0.443
week 4 of the year	–3.67	20.49	–0.18	0.858
week 5 of the year	–24.55	19.05	–1.29	0.199
week 6 of the year	–25.31	18.84	–1.34	0.181
week 7 of the year	–28.04	18.83	–1.49	0.139

Residential burglary in Chicago, IL	estimate	SE	statistic	p value
week 8 of the year	1.60	19.02	0.08	0.933
week 9 of the year	–83.18	21.04	–3.95	<0.001
week 10 of the year	–67.19	20.19	–3.33	0.001
week 11 of the year	–13.68	18.61	–0.74	0.463
week 12 of the year	–54.82	19.60	–2.80	0.006
week 13 of the year	–24.38	19.88	–1.23	0.222
week 14 of the year	–87.05	20.10	–4.33	<0.001
week 15 of the year	–113.45	20.22	–5.61	<0.001
week 16 of the year	–92.50	20.36	–4.54	<0.001
week 17 of the year	–138.13	21.62	–6.39	<0.001
week 18 of the year	–125.11	20.34	–6.15	<0.001
week 19 of the year	–122.34	20.38	–6.00	<0.001
week 20 of the year	–123.22	20.43	–6.03	<0.001
week 21 of the year	–77.53	20.56	–3.77	<0.001
week 22 of the year	–93.71	20.66	–4.54	<0.001
week 23 of the year	–116.13	20.50	–5.67	<0.001
week 24 of the year	–110.57	20.71	–5.34	<0.001
week 25 of the year	–50.76	20.62	–2.46	0.015
week 26 of the year	–66.46	20.52	–3.24	0.001
week 27 of the year	–91.77	20.51	–4.48	<0.001
week 28 of the year	–41.11	20.49	–2.01	0.047
week 29 of the year	–51.90	20.48	–2.53	0.012
week 30 of the year	–51.24	20.46	–2.50	0.013
week 31 of the year	–31.37	22.49	–1.39	0.165
week 32 of the year	–43.17	20.39	–2.12	0.036
week 33 of the year	–45.55	20.35	–2.24	0.027
week 34 of the year	–17.86	20.31	–0.88	0.380
week 35 of the year	13.51	20.25	0.67	0.506
week 36 of the year	–2.99	22.29	–0.13	0.894
week 37 of the year	–1.97	20.14	–0.10	0.922
week 38 of the year	26.09	20.08	1.30	0.196
week 39 of the year	6.33	19.98	0.32	0.752
week 40 of the year	–5.59	19.88	–0.28	0.779
week 41 of the year	20.37	19.78	1.03	0.305
week 42 of the year	28.68	19.68	1.46	0.147
week 43 of the year	24.72	19.57	1.26	0.208
week 44 of the year	36.10	19.41	1.86	0.065
week 45 of the year	–41.17	21.51	–1.91	0.058
week 46 of the year	26.91	19.04	1.41	0.160
week 47 of the year	9.42	18.85	0.50	0.618
week 48 of the year	11.43	18.87	0.61	0.546
week 49 of the year	4.91	18.98	0.26	0.796
week 50 of the year	–14.27	19.07	–0.75	0.455
week 51 of the year	12.35	17.61	0.70	0.484
week 52 of the year	5.39	15.18	0.35	0.723
holiday in week	–3.30	9.38	–0.35	0.725

Residential burglary in Los Angeles, CA	estimate	SE	statistic	p value
intercept	249.06	9.66	25.78	<0.001
AR(1)	0.17	0.07	2.54	0.012
AR(2)	0.17	0.07	2.47	0.014

Residential burglary in Los Angeles, CA	estimate	SE	statistic	p value
AR(3)	0.22	0.07	3.22	0.002
AR(4)	0.16	0.07	2.33	0.021
SAR(1)	–0.40	0.07	–5.61	<0.001
linear trend	–0.23	0.05	–4.24	<0.001
week 2 of the year	–7.67	8.15	–0.94	0.348
week 3 of the year	–13.37	8.19	–1.63	0.105
week 4 of the year	0.11	8.91	0.01	0.990
week 5 of the year	9.45	8.25	1.15	0.254
week 6 of the year	–10.61	8.80	–1.21	0.230
week 7 of the year	19.15	8.89	2.15	0.033
week 8 of the year	34.24	9.02	3.80	<0.001
week 9 of the year	–26.92	10.51	–2.56	0.011
week 10 of the year	–28.05	10.00	–2.80	0.006
week 11 of the year	–12.45	9.08	–1.37	0.172
week 12 of the year	–13.75	9.71	–1.42	0.159
week 13 of the year	5.55	9.82	0.57	0.573
week 14 of the year	–5.80	9.83	–0.59	0.556
week 15 of the year	–12.09	9.89	–1.22	0.224
week 16 of the year	–13.62	10.01	–1.36	0.176
week 17 of the year	–26.49	10.81	–2.45	0.015
week 18 of the year	–15.82	10.10	–1.57	0.119
week 19 of the year	–26.08	10.15	–2.57	0.011
week 20 of the year	–32.97	10.20	–3.23	0.002
week 21 of the year	–16.71	10.26	–1.63	0.106
week 22 of the year	–10.94	10.33	–1.06	0.291
week 23 of the year	–23.69	10.25	–2.31	0.022
week 24 of the year	–25.82	10.36	–2.49	0.014
week 25 of the year	–28.99	10.32	–2.81	0.006
week 26 of the year	–9.84	10.27	–0.96	0.339
week 27 of the year	–11.30	10.27	–1.10	0.273
week 28 of the year	–26.68	10.25	–2.60	0.010
week 29 of the year	–16.53	10.25	–1.61	0.109
week 30 of the year	6.86	10.24	0.67	0.504
week 31 of the year	–32.86	11.42	–2.88	0.005
week 32 of the year	–30.09	10.19	–2.95	0.004
week 33 of the year	–26.90	10.17	–2.65	0.009
week 34 of the year	–13.30	10.14	–1.31	0.192
week 35 of the year	–16.95	10.10	–1.68	0.095
week 36 of the year	–20.48	11.29	–1.81	0.072
week 37 of the year	–20.79	10.01	–2.08	0.040
week 38 of the year	–11.95	9.95	–1.20	0.232
week 39 of the year	–13.49	9.89	–1.36	0.175
week 40 of the year	–2.87	9.83	–0.29	0.770
week 41 of the year	–13.68	9.74	–1.40	0.162
week 42 of the year	–13.98	9.63	–1.45	0.149
week 43 of the year	–23.91	9.53	–2.51	0.013
week 44 of the year	–7.51	9.45	–0.79	0.428
week 45 of the year	–26.73	10.57	–2.53	0.013
week 46 of the year	–3.65	9.05	–0.40	0.688
week 47 of the year	–2.16	8.92	–0.24	0.809
week 48 of the year	–12.74	8.83	–1.44	0.151
week 49 of the year	–13.63	8.19	–1.66	0.098

Residential burglary in Los Angeles, CA	estimate	SE	statistic	p value
week 50 of the year	–29.92	7.97	–3.75	<0.001
week 51 of the year	–19.32	8.19	–2.36	0.020
week 52 of the year	–9.05	8.15	–1.11	0.269
holiday in week	–6.00	5.09	–1.18	0.240

Residential burglary in Louisville, KY	estimate	SE	statistic	p value
intercept	93.44	3.73	25.06	<0.001
AR(1)	0.12	0.07	1.75	0.081
AR(2)	–0.09	0.07	–1.30	0.196
AR(3)	0.11	0.07	1.62	0.107
SAR(1)	–0.36	0.08	–4.52	<0.001
linear trend	–0.20	0.01	–18.79	<0.001
week 2 of the year	1.13	4.68	0.24	0.810
week 3 of the year	–2.95	5.10	–0.58	0.564
week 4 of the year	–1.94	5.22	–0.37	0.711
week 5 of the year	–1.92	4.91	–0.39	0.696
week 6 of the year	–5.20	4.97	–1.05	0.297
week 7 of the year	–2.57	4.92	–0.52	0.602
week 8 of the year	1.52	4.94	0.31	0.758
week 9 of the year	–13.99	5.70	–2.46	0.015
week 10 of the year	–9.68	5.24	–1.85	0.067
week 11 of the year	3.22	4.66	0.69	0.491
week 12 of the year	–13.51	4.98	–2.71	0.007
week 13 of the year	–2.80	4.98	–0.56	0.575
week 14 of the year	–9.52	4.96	–1.92	0.057
week 15 of the year	–7.31	4.99	–1.47	0.145
week 16 of the year	–9.80	4.99	–1.96	0.051
week 17 of the year	–12.46	5.45	–2.29	0.024
week 18 of the year	–11.05	4.96	–2.23	0.028
week 19 of the year	–9.17	4.96	–1.85	0.066
week 20 of the year	–16.97	4.95	–3.43	<0.001
week 21 of the year	–6.08	5.00	–1.22	0.225
week 22 of the year	–5.14	5.03	–1.02	0.308
week 23 of the year	–8.28	4.95	–1.67	0.097
week 24 of the year	–3.30	5.03	–0.66	0.513
week 25 of the year	–5.24	4.99	–1.05	0.295
week 26 of the year	3.45	4.95	0.70	0.486
week 27 of the year	1.66	4.95	0.34	0.738
week 28 of the year	7.57	4.95	1.53	0.128
week 29 of the year	–1.05	4.96	–0.21	0.832
week 30 of the year	3.26	4.95	0.66	0.510
week 31 of the year	–6.34	5.72	–1.11	0.269
week 32 of the year	–11.78	4.95	–2.38	0.018
week 33 of the year	5.47	4.95	1.10	0.271
week 34 of the year	3.66	4.95	0.74	0.461
week 35 of the year	10.71	4.95	2.16	0.032
week 36 of the year	–9.47	5.72	–1.66	0.100
week 37 of the year	1.74	4.96	0.35	0.726
week 38 of the year	–0.53	4.95	–0.11	0.915
week 39 of the year	7.34	4.94	1.48	0.140
week 40 of the year	5.28	4.94	1.07	0.287

Residential burglary in Louisville, KY	estimate	SE	statistic	p value
week 41 of the year	4.66	4.95	0.94	0.348
week 42 of the year	6.08	4.94	1.23	0.220
week 43 of the year	–3.31	4.94	–0.67	0.504
week 44 of the year	–2.50	4.94	–0.51	0.614
week 45 of the year	–10.31	5.72	–1.80	0.073
week 46 of the year	–0.65	4.93	–0.13	0.896
week 47 of the year	–3.66	4.93	–0.74	0.459
week 48 of the year	–3.04	4.98	–0.61	0.543
week 49 of the year	–4.76	4.88	–0.98	0.331
week 50 of the year	–5.93	4.70	–1.26	0.209
week 51 of the year	–0.36	5.11	–0.07	0.945
week 52 of the year	0.34	4.67	0.07	0.942
holiday in week	4.17	2.87	1.46	0.147

Residential burglary in Memphis, TN	estimate	SE	statistic	p value
intercept	148.16	5.60	26.47	<0.001
AR(1)	0.63	0.16	4.06	<0.001
MA(1)	–0.40	0.18	–2.23	0.027
SAR(1)	–0.33	0.08	–4.08	<0.001
linear trend	–0.24	0.02	–11.64	<0.001
week 2 of the year	1.33	6.03	0.22	0.826
week 3 of the year	13.23	6.43	2.06	0.041
week 4 of the year	2.16	7.27	0.30	0.766
week 5 of the year	21.12	6.86	3.08	0.002
week 6 of the year	16.17	6.90	2.34	0.020
week 7 of the year	14.72	6.96	2.12	0.036
week 8 of the year	14.00	6.99	2.00	0.047
week 9 of the year	–14.42	7.87	–1.83	0.069
week 10 of the year	0.04	7.34	0.01	0.995
week 11 of the year	7.78	6.65	1.17	0.244
week 12 of the year	–0.93	7.02	–0.13	0.895
week 13 of the year	6.19	7.10	0.87	0.385
week 14 of the year	14.53	7.11	2.05	0.043
week 15 of the year	1.94	7.12	0.27	0.785
week 16 of the year	–0.28	7.16	–0.04	0.969
week 17 of the year	–19.33	7.67	–2.52	0.013
week 18 of the year	–26.82	7.10	–3.78	<0.001
week 19 of the year	–25.35	7.10	–3.57	<0.001
week 20 of the year	–33.54	7.13	–4.71	<0.001
week 21 of the year	–18.06	7.14	–2.53	0.012
week 22 of the year	–2.53	7.17	–0.35	0.725
week 23 of the year	–14.62	7.09	–2.06	0.041
week 24 of the year	–6.73	7.16	–0.94	0.349
week 25 of the year	4.51	7.12	0.63	0.528
week 26 of the year	2.40	7.08	0.34	0.735
week 27 of the year	0.50	7.08	0.07	0.944
week 28 of the year	3.17	7.08	0.45	0.655
week 29 of the year	12.35	7.08	1.74	0.083
week 30 of the year	21.51	7.07	3.04	0.003
week 31 of the year	24.02	7.98	3.01	0.003
week 32 of the year	3.06	7.07	0.43	0.666

Residential burglary in Memphis, TN	estimate	SE	statistic	p value
week 33 of the year	–14.26	7.07	–2.02	0.045
week 34 of the year	14.55	7.07	2.06	0.041
week 35 of the year	4.93	7.07	0.70	0.487
week 36 of the year	–1.55	7.98	–0.19	0.846
week 37 of the year	3.63	7.07	0.51	0.608
week 38 of the year	13.94	7.07	1.97	0.050
week 39 of the year	6.39	7.06	0.91	0.367
week 40 of the year	–4.01	7.06	–0.57	0.571
week 41 of the year	7.44	7.05	1.06	0.293
week 42 of the year	4.20	7.07	0.59	0.554
week 43 of the year	18.00	7.04	2.55	0.012
week 44 of the year	10.60	7.04	1.51	0.134
week 45 of the year	–6.31	7.95	–0.79	0.429
week 46 of the year	7.79	7.00	1.11	0.267
week 47 of the year	7.39	6.96	1.06	0.290
week 48 of the year	0.35	6.91	0.05	0.960
week 49 of the year	2.09	6.82	0.31	0.759
week 50 of the year	–2.66	6.69	–0.40	0.692
week 51 of the year	–0.06	6.44	–0.01	0.993
week 52 of the year	4.48	6.07	0.74	0.461
holiday in week	–5.99	3.70	–1.62	0.107

Residential burglary in Minneapolis, MN	estimate	SE	statistic	p value
intercept	71.89	3.37	21.35	<0.001
AR(1)	0.31	0.05	6.15	<0.001
AR(2)	0.06	0.05	1.15	0.250
SAR(1)	–0.51	0.04	–11.63	<0.001
linear trend	–0.07	0.01	–5.57	<0.001
week 2 of the year	1.26	3.47	0.36	0.716
week 3 of the year	–1.85	3.88	–0.48	0.634
week 4 of the year	–1.55	4.50	–0.34	0.731
week 5 of the year	–19.24	4.20	–4.58	<0.001
week 6 of the year	–16.28	4.21	–3.87	<0.001
week 7 of the year	–19.03	4.23	–4.50	<0.001
week 8 of the year	–8.53	4.23	–2.02	0.045
week 9 of the year	–29.55	4.76	–6.20	<0.001
week 10 of the year	–32.32	4.46	–7.25	<0.001
week 11 of the year	–31.68	3.99	–7.93	<0.001
week 12 of the year	–31.01	4.24	–7.31	<0.001
week 13 of the year	–31.85	4.25	–7.50	<0.001
week 14 of the year	–29.11	4.26	–6.84	<0.001
week 15 of the year	–30.17	4.26	–7.07	<0.001
week 16 of the year	–34.73	4.29	–8.10	<0.001
week 17 of the year	–37.13	4.65	–7.98	<0.001
week 18 of the year	–28.32	4.26	–6.64	<0.001
week 19 of the year	–30.16	4.26	–7.07	<0.001
week 20 of the year	–25.37	4.26	–5.95	<0.001
week 21 of the year	–24.11	4.29	–5.62	<0.001
week 22 of the year	–26.79	4.32	–6.21	<0.001
week 23 of the year	–16.60	4.26	–3.90	<0.001
week 24 of the year	–22.84	4.31	–5.29	<0.001

Residential burglary in Minneapolis, MN	estimate	SE	statistic	p value
week 25 of the year	–17.54	4.28	–4.10	<0.001
week 26 of the year	–20.28	4.26	–4.77	<0.001
week 27 of the year	–23.63	4.26	–5.55	<0.001
week 28 of the year	–15.53	4.25	–3.65	<0.001
week 29 of the year	–13.93	4.25	–3.27	0.001
week 30 of the year	–12.99	4.25	–3.06	0.003
week 31 of the year	–14.76	4.85	–3.04	0.003
week 32 of the year	–15.88	4.25	–3.74	<0.001
week 33 of the year	–10.78	4.25	–2.54	0.012
week 34 of the year	–15.72	4.25	–3.70	<0.001
week 35 of the year	–4.28	4.25	–1.01	0.315
week 36 of the year	–13.90	4.85	–2.87	0.005
week 37 of the year	–14.32	4.25	–3.37	<0.001
week 38 of the year	–8.85	4.25	–2.08	0.039
week 39 of the year	–7.00	4.25	–1.65	0.101
week 40 of the year	–6.22	4.25	–1.46	0.145
week 41 of the year	–2.93	4.24	–0.69	0.491
week 42 of the year	0.35	4.24	0.08	0.934
week 43 of the year	–6.31	4.24	–1.49	0.139
week 44 of the year	12.93	4.25	3.04	0.003
week 45 of the year	–5.91	4.84	–1.22	0.224
week 46 of the year	5.41	4.24	1.28	0.204
week 47 of the year	–2.82	4.23	–0.67	0.506
week 48 of the year	0.36	4.21	0.09	0.932
week 49 of the year	–7.67	4.17	–1.84	0.068
week 50 of the year	–4.51	4.09	–1.10	0.272
week 51 of the year	4.17	3.88	1.08	0.283
week 52 of the year	3.63	3.47	1.05	0.296
holiday in week	4.43	2.34	1.90	0.060

Residential burglary in Montgomery County, MD	estimate	SE	statistic	p value
intercept	30.43	1.73	17.63	<0.001
AR(1)	0.10	0.07	1.31	0.191
AR(2)	0.10	0.07	1.38	0.171
AR(3)	0.13	0.08	1.77	0.079
SAR(1)	–0.49	0.08	–6.02	<0.001
linear trend	–0.05	0.01	–7.36	<0.001
week 2 of the year	–6.05	1.95	–3.10	0.002
week 3 of the year	–1.09	1.95	–0.56	0.577
week 4 of the year	–2.25	2.36	–0.95	0.343
week 5 of the year	0.28	2.07	0.14	0.892
week 6 of the year	–2.65	2.05	–1.29	0.199
week 7 of the year	–2.79	2.06	–1.36	0.178
week 8 of the year	–2.10	2.08	–1.01	0.314
week 9 of the year	–5.50	2.70	–2.04	0.044
week 10 of the year	–2.61	2.72	–0.96	0.338
week 11 of the year	–2.48	2.09	–1.19	0.238
week 12 of the year	–1.13	2.26	–0.50	0.618
week 13 of the year	–1.73	2.35	–0.74	0.464
week 14 of the year	–1.49	2.30	–0.65	0.518
week 15 of the year	–8.15	2.30	–3.54	<0.001

Residential burglary in Montgomery County, MD	estimate	SE	statistic	p value
week 16 of the year	1.79	2.31	0.78	0.438
week 17 of the year	–6.52	2.86	–2.28	0.024
week 18 of the year	–5.43	2.31	–2.35	0.020
week 19 of the year	–9.21	2.31	–3.99	<0.001
week 20 of the year	–4.75	2.32	–2.05	0.042
week 21 of the year	–10.66	2.30	–4.63	<0.001
week 22 of the year	–8.43	2.42	–3.49	<0.001
week 23 of the year	–6.28	2.31	–2.72	0.007
week 24 of the year	–4.80	2.36	–2.04	0.044
week 25 of the year	–10.34	2.35	–4.39	<0.001
week 26 of the year	–6.96	2.30	–3.02	0.003
week 27 of the year	–4.04	2.30	–1.76	0.082
week 28 of the year	–2.44	2.30	–1.06	0.292
week 29 of the year	–6.83	2.31	–2.96	0.004
week 30 of the year	–0.79	2.32	–0.34	0.733
week 31 of the year	–5.60	2.88	–1.95	0.054
week 32 of the year	0.31	2.31	0.14	0.893
week 33 of the year	–1.63	2.29	–0.71	0.478
week 34 of the year	–8.29	2.29	–3.61	<0.001
week 35 of the year	–8.08	2.10	–3.85	<0.001
week 36 of the year	–6.52	2.71	–2.41	0.017
week 37 of the year	–3.76	2.09	–1.80	0.075
week 38 of the year	–4.02	2.10	–1.92	0.057
week 39 of the year	0.21	2.10	0.10	0.919
week 40 of the year	–6.61	2.09	–3.16	0.002
week 41 of the year	–1.26	2.09	–0.60	0.548
week 42 of the year	0.30	2.09	0.15	0.885
week 43 of the year	–3.76	2.09	–1.80	0.075
week 44 of the year	–2.09	2.08	–1.00	0.318
week 45 of the year	–7.93	2.71	–2.93	0.004
week 46 of the year	–0.09	2.08	–0.04	0.965
week 47 of the year	–5.92	2.06	–2.87	0.005
week 48 of the year	–5.47	2.05	–2.67	0.009
week 49 of the year	4.49	2.04	2.20	0.030
week 50 of the year	0.24	1.92	0.12	0.902
week 51 of the year	–1.60	1.95	–0.82	0.412
week 52 of the year	–3.85	1.95	–1.97	0.051
holiday in week	0.61	1.73	0.35	0.725

Residential burglary in Phoenix, AZ	estimate	SE	statistic	p value
intercept	187.85	5.00	37.59	<0.001
AR(1)	0.27	NA	NA	NA
SAR(1)	–0.44	NA	NA	NA
linear trend	–0.39	0.02	–23.94	<0.001
week 2 of the year	1.79	5.55	0.32	0.748
week 3 of the year	–6.72	6.24	–1.08	0.283
week 4 of the year	–14.66	7.02	–2.09	0.038
week 5 of the year	–0.83	6.50	–0.13	0.898
week 6 of the year	–6.13	6.47	–0.95	0.345
week 7 of the year	6.72	6.48	1.04	0.301
week 8 of the year	18.57	6.48	2.87	0.005

Residential burglary in Phoenix, AZ	estimate	SE	statistic	p value
week 9 of the year	–24.78	7.31	–3.39	<0.001
week 10 of the year	–12.77	6.81	–1.88	0.063
week 11 of the year	–4.29	6.10	–0.70	0.484
week 12 of the year	–0.94	6.49	–0.14	0.886
week 13 of the year	–6.33	6.50	–0.97	0.332
week 14 of the year	10.84	6.50	1.67	0.098
week 15 of the year	–7.00	6.51	–1.08	0.284
week 16 of the year	–1.99	6.55	–0.30	0.762
week 17 of the year	–14.53	7.10	–2.05	0.042
week 18 of the year	–15.02	6.50	–2.31	0.022
week 19 of the year	–16.52	6.50	–2.54	0.012
week 20 of the year	–41.72	6.50	–6.42	<0.001
week 21 of the year	–17.40	6.54	–2.66	0.009
week 22 of the year	8.40	6.58	1.28	0.204
week 23 of the year	–10.68	6.50	–1.64	0.102
week 24 of the year	–10.28	6.58	–1.56	0.120
week 25 of the year	12.00	6.53	1.84	0.068
week 26 of the year	8.21	6.49	1.26	0.208
week 27 of the year	8.67	6.49	1.34	0.184
week 28 of the year	–2.28	6.49	–0.35	0.726
week 29 of the year	7.56	6.49	1.16	0.246
week 30 of the year	15.33	6.49	2.36	0.019
week 31 of the year	–14.42	7.41	–1.95	0.053
week 32 of the year	–3.38	6.49	–0.52	0.603
week 33 of the year	–1.90	6.49	–0.29	0.770
week 34 of the year	4.29	6.48	0.66	0.509
week 35 of the year	12.88	6.48	1.99	0.049
week 36 of the year	–7.33	7.41	–0.99	0.324
week 37 of the year	–11.29	6.48	–1.74	0.084
week 38 of the year	–15.39	6.48	–2.37	0.019
week 39 of the year	–4.15	6.48	–0.64	0.523
week 40 of the year	–5.07	6.48	–0.78	0.435
week 41 of the year	–3.30	6.48	–0.51	0.612
week 42 of the year	–3.72	6.48	–0.57	0.567
week 43 of the year	6.10	6.48	0.94	0.348
week 44 of the year	9.37	6.48	1.45	0.150
week 45 of the year	–10.82	7.40	–1.46	0.146
week 46 of the year	–1.03	6.48	–0.16	0.874
week 47 of the year	–1.24	6.48	–0.19	0.849
week 48 of the year	–2.68	6.47	–0.41	0.679
week 49 of the year	1.42	6.46	0.22	0.826
week 50 of the year	–5.49	6.42	–0.86	0.393
week 51 of the year	–0.07	6.24	–0.01	0.991
week 52 of the year	0.09	5.55	0.02	0.988
holiday in week	1.31	3.58	0.37	0.715

Residential burglary in Sacramento, CA	estimate	SE	statistic	p value
intercept	44.82	2.94	15.25	<0.001
AR(1)	0.86	0.08	10.64	<0.001
MA(1)	–0.71	0.10	–7.08	<0.001
SAR(1)	–0.35	0.08	–4.23	<0.001

Residential burglary in Sacramento, CA	estimate	SE	statistic	p value
linear trend	–0.07	0.01	–5.20	<0.001
week 2 of the year	–5.47	3.17	–1.72	0.087
week 3 of the year	–9.17	3.23	–2.84	0.005
week 4 of the year	–3.70	3.61	–1.02	0.308
week 5 of the year	–0.48	3.34	–0.14	0.886
week 6 of the year	–4.19	3.35	–1.25	0.213
week 7 of the year	–4.38	3.38	–1.30	0.196
week 8 of the year	2.11	3.40	0.62	0.537
week 9 of the year	–3.54	3.93	–0.90	0.370
week 10 of the year	–3.14	3.66	–0.86	0.392
week 11 of the year	–6.78	3.28	–2.07	0.041
week 12 of the year	–5.25	3.51	–1.50	0.136
week 13 of the year	–0.30	3.53	–0.09	0.932
week 14 of the year	–4.66	3.51	–1.33	0.186
week 15 of the year	–0.59	3.52	–0.17	0.868
week 16 of the year	–1.85	3.55	–0.52	0.604
week 17 of the year	–1.68	3.85	–0.44	0.663
week 18 of the year	–3.62	3.54	–1.02	0.309
week 19 of the year	–8.53	3.54	–2.41	0.017
week 20 of the year	–2.35	3.56	–0.66	0.510
week 21 of the year	0.85	3.58	0.24	0.812
week 22 of the year	–6.75	3.60	–1.87	0.063
week 23 of the year	–9.39	3.55	–2.64	0.009
week 24 of the year	–1.80	3.60	–0.50	0.617
week 25 of the year	–7.52	3.58	–2.10	0.037
week 26 of the year	–2.04	3.55	–0.57	0.566
week 27 of the year	–0.83	3.55	–0.23	0.817
week 28 of the year	0.51	3.55	0.14	0.887
week 29 of the year	–1.16	3.54	–0.33	0.743
week 30 of the year	2.45	3.55	0.69	0.492
week 31 of the year	–1.83	4.05	–0.45	0.652
week 32 of the year	–6.64	3.54	–1.87	0.063
week 33 of the year	–6.06	3.54	–1.71	0.089
week 34 of the year	–2.84	3.53	–0.80	0.422
week 35 of the year	1.28	3.53	0.36	0.716
week 36 of the year	–2.09	4.05	–0.52	0.606
week 37 of the year	2.86	3.52	0.81	0.417
week 38 of the year	–2.27	3.51	–0.65	0.518
week 39 of the year	2.76	3.51	0.79	0.433
week 40 of the year	–5.10	3.50	–1.46	0.147
week 41 of the year	–5.99	3.48	–1.72	0.087
week 42 of the year	0.36	3.47	0.10	0.917
week 43 of the year	–5.64	3.46	–1.63	0.105
week 44 of the year	–4.98	3.44	–1.45	0.150
week 45 of the year	–7.53	3.96	–1.90	0.059
week 46 of the year	–9.01	3.40	–2.65	0.009
week 47 of the year	–9.29	3.38	–2.75	0.007
week 48 of the year	–3.33	3.35	–0.99	0.322
week 49 of the year	–6.35	3.32	–1.92	0.057
week 50 of the year	–4.76	3.28	–1.45	0.148
week 51 of the year	–2.47	3.23	–0.76	0.446
week 52 of the year	–3.74	3.17	–1.18	0.241

Residential burglary in Sacramento, CA	estimate	SE	statistic	p value
holiday in week	1.74	1.97	0.89	0.378

Residential burglary in San Francisco, CA	estimate	SE	statistic	p value
intercept	42.61	3.59	11.86	<0.001
AR(1)	0.84	0.07	11.25	<0.001
MA(1)	–0.54	0.12	–4.41	<0.001
SAR(1)	–0.40	0.07	–5.56	<0.001
linear trend	–0.02	0.02	–1.06	0.292
week 2 of the year	11.13	3.09	3.60	<0.001
week 3 of the year	2.56	3.26	0.78	0.435
week 4 of the year	10.58	3.74	2.83	0.005
week 5 of the year	0.01	3.54	0.00	0.998
week 6 of the year	21.30	3.61	5.90	<0.001
week 7 of the year	22.63	3.68	6.14	<0.001
week 8 of the year	22.15	3.74	5.93	<0.001
week 9 of the year	6.11	4.23	1.44	0.151
week 10 of the year	18.47	4.02	4.59	<0.001
week 11 of the year	9.94	3.69	2.69	0.008
week 12 of the year	20.01	3.91	5.12	<0.001
week 13 of the year	14.32	3.95	3.63	<0.001
week 14 of the year	17.06	3.97	4.30	<0.001
week 15 of the year	16.58	4.00	4.15	<0.001
week 16 of the year	19.96	4.04	4.94	<0.001
week 17 of the year	26.72	4.31	6.20	<0.001
week 18 of the year	14.51	4.04	3.59	<0.001
week 19 of the year	15.07	4.06	3.72	<0.001
week 20 of the year	15.51	4.06	3.82	<0.001
week 21 of the year	13.44	4.08	3.29	0.001
week 22 of the year	19.44	4.11	4.73	<0.001
week 23 of the year	8.19	4.08	2.01	0.046
week 24 of the year	9.94	4.12	2.41	0.017
week 25 of the year	9.48	4.09	2.32	0.022
week 26 of the year	11.63	4.09	2.84	0.005
week 27 of the year	17.77	4.09	4.35	<0.001
week 28 of the year	12.42	4.08	3.04	0.003
week 29 of the year	18.02	4.08	4.42	<0.001
week 30 of the year	14.66	4.07	3.61	<0.001
week 31 of the year	11.41	4.51	2.53	0.012
week 32 of the year	8.99	4.06	2.21	0.028
week 33 of the year	3.97	4.06	0.98	0.330
week 34 of the year	6.61	4.05	1.63	0.105
week 35 of the year	8.97	4.04	2.22	0.028
week 36 of the year	11.46	4.48	2.56	0.012
week 37 of the year	10.09	4.01	2.51	0.013
week 38 of the year	12.27	4.00	3.07	0.003
week 39 of the year	6.04	4.00	1.51	0.133
week 40 of the year	6.10	3.97	1.54	0.126
week 41 of the year	9.03	3.94	2.29	0.023
week 42 of the year	12.47	3.92	3.18	0.002
week 43 of the year	10.48	3.89	2.69	0.008
week 44 of the year	21.96	3.86	5.69	<0.001

Residential burglary in San Francisco, CA	estimate	SE	statistic	p value
week 45 of the year	12.08	4.29	2.82	0.006
week 46 of the year	10.49	3.75	2.80	0.006
week 47 of the year	20.97	3.69	5.68	<0.001
week 48 of the year	15.48	3.62	4.28	<0.001
week 49 of the year	16.29	3.52	4.62	<0.001
week 50 of the year	9.18	3.41	2.69	0.008
week 51 of the year	14.98	3.27	4.58	<0.001
week 52 of the year	11.91	3.08	3.86	<0.001
holiday in week	–0.77	1.97	–0.39	0.698

Non-residential burglary

Non-residential burglary in Atlanta, GA	estimate	SE	statistic	p value
intercept	16.67	1.72	9.70	<0.001
AR(1)	0.08	0.07	1.19	0.234
AR(2)	0.24	0.07	3.23	0.002
SAR(1)	–0.43	0.08	–5.70	<0.001
linear trend	–0.02	0.01	–4.06	<0.001
week 2 of the year	2.38	2.07	1.15	0.253
week 3 of the year	0.82	1.90	0.43	0.665
week 4 of the year	–4.27	2.40	–1.78	0.078
week 5 of the year	1.24	2.14	0.58	0.562
week 6 of the year	1.15	2.17	0.53	0.598
week 7 of the year	3.71	2.18	1.70	0.090
week 8 of the year	5.76	2.18	2.65	0.009
week 9 of the year	4.56	2.59	1.76	0.080
week 10 of the year	0.54	2.27	0.24	0.813
week 11 of the year	4.06	2.20	1.85	0.066
week 12 of the year	1.88	2.43	0.78	0.439
week 13 of the year	1.20	2.22	0.54	0.589
week 14 of the year	1.93	2.20	0.88	0.382
week 15 of the year	0.83	2.20	0.38	0.707
week 16 of the year	3.65	2.22	1.64	0.102
week 17 of the year	–2.41	2.45	–0.98	0.327
week 18 of the year	–0.73	2.20	–0.33	0.741
week 19 of the year	–1.32	2.20	–0.60	0.550
week 20 of the year	–0.26	2.20	–0.12	0.905
week 21 of the year	–1.09	2.22	–0.49	0.624
week 22 of the year	–0.09	2.23	–0.04	0.969
week 23 of the year	1.90	2.20	0.87	0.388
week 24 of the year	0.79	2.23	0.35	0.725
week 25 of the year	–1.19	2.21	–0.54	0.591
week 26 of the year	–3.82	2.20	–1.74	0.084
week 27 of the year	0.62	2.20	0.28	0.779
week 28 of the year	1.94	2.19	0.88	0.379
week 29 of the year	–0.89	2.19	–0.41	0.685
week 30 of the year	4.90	2.19	2.23	0.027
week 31 of the year	8.74	2.58	3.39	<0.001
week 32 of the year	2.62	2.19	1.20	0.234
week 33 of the year	4.88	2.19	2.23	0.028
week 34 of the year	3.32	2.19	1.51	0.132
week 35 of the year	2.26	2.19	1.03	0.305
week 36 of the year	–0.43	2.60	–0.17	0.868
week 37 of the year	1.16	2.19	0.53	0.598
week 38 of the year	5.95	2.19	2.71	0.007
week 39 of the year	2.18	2.19	0.99	0.322
week 40 of the year	5.26	2.19	2.40	0.017
week 41 of the year	–3.24	2.19	–1.48	0.141
week 42 of the year	3.14	2.19	1.43	0.154
week 43 of the year	1.31	2.19	0.60	0.551
week 44 of the year	9.48	2.19	4.33	<0.001
week 45 of the year	0.00	2.58	0.00	>0.999
week 46 of the year	5.00	2.19	2.28	0.024

Non-residential burglary in Atlanta, GA	estimate	SE	statistic	p value
week 47 of the year	0.38	2.17	0.17	0.863
week 48 of the year	–2.01	2.17	–0.92	0.357
week 49 of the year	–0.66	2.12	–0.31	0.755
week 50 of the year	2.01	2.14	0.94	0.349
week 51 of the year	0.51	1.90	0.27	0.791
week 52 of the year	–0.97	2.07	–0.47	0.640
holiday in week	0.27	1.36	0.20	0.843

Non-residential burglary in Austin, TX	estimate	SE	statistic	p value
intercept	35.61	3.22	11.05	<0.001
AR(1)	0.87	0.10	9.07	<0.001
MA(1)	–0.73	0.13	–5.72	<0.001
SAR(1)	–0.33	0.08	–4.16	<0.001
linear trend	0.03	0.01	2.30	0.023
week 2 of the year	–1.50	3.47	–0.43	0.666
week 3 of the year	–7.49	3.52	–2.13	0.035
week 4 of the year	–0.32	3.93	–0.08	0.934
week 5 of the year	1.46	3.63	0.40	0.688
week 6 of the year	–9.63	3.64	–2.65	0.009
week 7 of the year	–7.78	3.67	–2.12	0.036
week 8 of the year	–2.32	3.69	–0.63	0.530
week 9 of the year	–0.98	4.27	–0.23	0.819
week 10 of the year	–2.61	3.97	–0.66	0.513
week 11 of the year	–4.18	3.57	–1.17	0.243
week 12 of the year	–5.88	3.81	–1.54	0.125
week 13 of the year	–6.47	3.82	–1.69	0.093
week 14 of the year	–8.21	3.81	–2.15	0.033
week 15 of the year	–5.77	3.82	–1.51	0.133
week 16 of the year	–2.92	3.86	–0.76	0.451
week 17 of the year	–8.28	4.18	–1.98	0.049
week 18 of the year	–8.05	3.84	–2.09	0.038
week 19 of the year	–9.74	3.85	–2.53	0.012
week 20 of the year	–5.28	3.85	–1.37	0.173
week 21 of the year	–8.78	3.88	–2.26	0.025
week 22 of the year	–9.74	3.90	–2.49	0.014
week 23 of the year	–7.48	3.86	–1.94	0.055
week 24 of the year	–11.53	3.91	–2.95	0.004
week 25 of the year	–7.80	3.89	–2.01	0.047
week 26 of the year	–9.23	3.86	–2.39	0.018
week 27 of the year	–2.99	3.87	–0.77	0.441
week 28 of the year	–5.72	3.86	–1.48	0.140
week 29 of the year	–16.33	3.86	–4.23	<0.001
week 30 of the year	–5.86	3.86	–1.52	0.130
week 31 of the year	–9.78	4.40	–2.23	0.028
week 32 of the year	1.58	3.85	0.41	0.681
week 33 of the year	5.17	3.86	1.34	0.183
week 34 of the year	–6.05	3.84	–1.58	0.117
week 35 of the year	–1.72	3.84	–0.45	0.655
week 36 of the year	–4.22	4.38	–0.97	0.336
week 37 of the year	–7.58	3.82	–1.98	0.049
week 38 of the year	–1.60	3.81	–0.42	0.676

Non-residential burglary in Austin, TX	estimate	SE	statistic	p value
week 39 of the year	–11.19	3.80	–2.94	0.004
week 40 of the year	–10.80	3.79	–2.85	0.005
week 41 of the year	–13.90	3.79	–3.67	<0.001
week 42 of the year	–11.64	3.77	–3.09	0.002
week 43 of the year	–8.74	3.76	–2.32	0.021
week 44 of the year	–12.76	3.74	–3.41	<0.001
week 45 of the year	–5.37	4.28	–1.26	0.211
week 46 of the year	–8.65	3.70	–2.34	0.021
week 47 of the year	–3.62	3.69	–0.98	0.328
week 48 of the year	–6.90	3.64	–1.90	0.060
week 49 of the year	–12.56	3.61	–3.48	<0.001
week 50 of the year	–7.06	3.57	–1.98	0.049
week 51 of the year	–3.93	3.52	–1.12	0.266
week 52 of the year	–9.22	3.47	–2.66	0.009
holiday in week	0.81	2.11	0.39	0.700

Non-residential burglary in Baltimore, MD	estimate	SE	statistic	p value
intercept	57.89	6.37	9.08	<0.001
AR(1)	1.31	0.34	3.87	<0.001
AR(2)	–0.38	0.19	–2.00	0.047
MA(1)	–0.85	0.32	–2.62	0.010
SAR(1)	–0.43	0.08	–5.44	<0.001
linear trend	–0.07	0.03	–2.57	0.011
week 2 of the year	–5.04	5.50	–0.92	0.361
week 3 of the year	–12.11	6.59	–1.84	0.068
week 4 of the year	–1.25	7.55	–0.17	0.869
week 5 of the year	–2.46	7.25	–0.34	0.735
week 6 of the year	–7.15	7.31	–0.98	0.330
week 7 of the year	–4.91	7.36	–0.67	0.506
week 8 of the year	25.45	7.44	3.42	<0.001
week 9 of the year	–17.63	8.06	–2.19	0.030
week 10 of the year	6.92	7.64	0.91	0.367
week 11 of the year	15.60	7.07	2.20	0.029
week 12 of the year	3.87	7.39	0.52	0.601
week 13 of the year	–10.52	7.49	–1.40	0.162
week 14 of the year	–15.34	7.60	–2.02	0.045
week 15 of the year	–14.71	7.63	–1.93	0.056
week 16 of the year	–12.04	7.67	–1.57	0.119
week 17 of the year	–8.12	8.12	–1.00	0.319
week 18 of the year	–7.38	7.63	–0.97	0.335
week 19 of the year	–14.22	7.62	–1.87	0.064
week 20 of the year	–14.34	7.61	–1.88	0.061
week 21 of the year	0.56	7.64	0.07	0.942
week 22 of the year	–8.07	7.67	–1.05	0.294
week 23 of the year	–6.88	7.60	–0.91	0.367
week 24 of the year	–5.11	7.66	–0.67	0.506
week 25 of the year	–15.55	7.62	–2.04	0.043
week 26 of the year	2.66	7.59	0.35	0.726
week 27 of the year	–10.51	7.58	–1.39	0.168
week 28 of the year	–4.51	7.58	–0.59	0.553
week 29 of the year	–4.39	7.57	–0.58	0.563

Non-residential burglary in Baltimore, MD	estimate	SE	statistic	p value
week 30 of the year	2.31	7.57	0.30	0.761
week 31 of the year	18.41	8.36	2.20	0.029
week 32 of the year	–1.18	7.56	–0.16	0.876
week 33 of the year	0.85	7.56	0.11	0.911
week 34 of the year	13.10	7.55	1.74	0.085
week 35 of the year	4.89	7.54	0.65	0.518
week 36 of the year	2.32	8.31	0.28	0.780
week 37 of the year	3.10	7.53	0.41	0.681
week 38 of the year	17.39	7.53	2.31	0.022
week 39 of the year	11.64	7.52	1.55	0.124
week 40 of the year	–2.54	7.51	–0.34	0.736
week 41 of the year	–2.33	7.49	–0.31	0.756
week 42 of the year	5.64	7.49	0.75	0.453
week 43 of the year	12.01	7.47	1.61	0.110
week 44 of the year	–1.22	7.45	–0.16	0.870
week 45 of the year	8.09	8.21	0.99	0.326
week 46 of the year	–1.25	7.40	–0.17	0.867
week 47 of the year	5.24	7.37	0.71	0.478
week 48 of the year	1.46	7.31	0.20	0.842
week 49 of the year	3.21	7.21	0.44	0.657
week 50 of the year	–5.98	7.02	–0.85	0.396
week 51 of the year	–1.94	6.59	–0.29	0.769
week 52 of the year	5.49	5.50	1.00	0.319
holiday in week	3.03	3.50	0.86	0.389

Non-residential burglary in Boston, MA	estimate	SE	statistic	p value
intercept	10.17	1.43	7.09	<0.001
AR(1)	0.16	0.07	2.25	0.026
AR(2)	0.07	0.07	0.99	0.324
AR(3)	0.10	0.07	1.41	0.160
AR(4)	0.17	0.07	2.41	0.017
SAR(1)	–0.35	0.08	–4.17	<0.001
linear trend	–0.02	0.01	–3.12	0.002
week 2 of the year	–1.98	1.62	–1.22	0.226
week 3 of the year	–5.02	1.69	–2.97	0.004
week 4 of the year	–2.57	1.95	–1.32	0.191
week 5 of the year	–1.09	1.61	–0.68	0.499
week 6 of the year	0.17	1.74	0.10	0.923
week 7 of the year	–3.01	1.76	–1.71	0.090
week 8 of the year	–2.69	1.76	–1.52	0.130
week 9 of the year	–6.32	2.01	–3.14	0.002
week 10 of the year	–3.03	1.86	–1.63	0.105
week 11 of the year	–2.56	1.68	–1.52	0.130
week 12 of the year	–1.74	1.86	–0.94	0.350
week 13 of the year	–3.90	1.70	–2.29	0.023
week 14 of the year	–2.73	1.69	–1.61	0.109
week 15 of the year	–2.12	1.70	–1.25	0.214
week 16 of the year	0.08	1.71	0.05	0.962
week 17 of the year	–7.23	1.87	–3.86	<0.001
week 18 of the year	–5.15	1.70	–3.03	0.003
week 19 of the year	–2.91	1.70	–1.72	0.089

Non-residential burglary in Boston, MA	estimate	SE	statistic	p value
week 20 of the year	–4.68	1.70	–2.75	0.007
week 21 of the year	–2.53	1.71	–1.48	0.142
week 22 of the year	–2.83	1.73	–1.64	0.103
week 23 of the year	–4.90	1.70	–2.88	0.005
week 24 of the year	–1.95	1.73	–1.13	0.262
week 25 of the year	0.28	1.72	0.16	0.869
week 26 of the year	0.70	1.71	0.41	0.684
week 27 of the year	0.12	1.70	0.07	0.946
week 28 of the year	–1.67	1.70	–0.98	0.329
week 29 of the year	–0.06	1.70	–0.03	0.973
week 30 of the year	–0.40	1.70	–0.24	0.814
week 31 of the year	–3.38	1.97	–1.72	0.088
week 32 of the year	–2.44	1.70	–1.43	0.154
week 33 of the year	–1.36	1.70	–0.80	0.426
week 34 of the year	–0.98	1.70	–0.57	0.567
week 35 of the year	4.43	1.71	2.60	0.010
week 36 of the year	–2.52	1.97	–1.28	0.203
week 37 of the year	–3.93	1.70	–2.32	0.022
week 38 of the year	–0.89	1.70	–0.52	0.603
week 39 of the year	0.33	1.69	0.20	0.844
week 40 of the year	–1.07	1.69	–0.63	0.527
week 41 of the year	–1.05	1.69	–0.62	0.533
week 42 of the year	0.78	1.69	0.46	0.646
week 43 of the year	–0.15	1.68	–0.09	0.928
week 44 of the year	–3.05	1.68	–1.81	0.072
week 45 of the year	–5.00	1.93	–2.59	0.011
week 46 of the year	1.25	1.65	0.76	0.449
week 47 of the year	2.57	1.65	1.56	0.122
week 48 of the year	–0.44	1.63	–0.27	0.789
week 49 of the year	–0.56	1.60	–0.35	0.729
week 50 of the year	1.40	1.66	0.84	0.403
week 51 of the year	2.96	1.69	1.75	0.082
week 52 of the year	0.49	1.62	0.30	0.764
holiday in week	3.45	0.99	3.47	<0.001

Non-residential burglary in Chicago, IL	estimate	SE	statistic	p value
intercept	105.15	5.41	19.44	<0.001
AR(1)	0.14	0.01	17.56	<0.001
AR(2)	0.06	0.02	3.55	<0.001
SAR(1)	–0.42	NA	NA	NA
linear trend	–0.17	0.02	–9.97	<0.001
week 2 of the year	13.53	6.55	2.07	0.041
week 3 of the year	5.57	6.80	0.82	0.414
week 4 of the year	14.14	7.77	1.82	0.071
week 5 of the year	21.97	7.11	3.09	0.002
week 6 of the year	–5.93	7.08	–0.84	0.404
week 7 of the year	15.00	7.08	2.12	0.036
week 8 of the year	22.32	7.08	3.15	0.002
week 9 of the year	35.68	8.18	4.36	<0.001
week 10 of the year	2.63	7.55	0.35	0.728
week 11 of the year	6.85	6.68	1.03	0.307

Non-residential burglary in Chicago, IL	estimate	SE	statistic	p value
week 12 of the year	–4.16	7.16	–0.58	0.562
week 13 of the year	8.34	7.14	1.17	0.244
week 14 of the year	–8.49	7.11	–1.19	0.234
week 15 of the year	–4.51	7.11	–0.63	0.527
week 16 of the year	–10.90	7.16	–1.52	0.130
week 17 of the year	–11.53	7.85	–1.47	0.144
week 18 of the year	–25.40	7.11	–3.57	<0.001
week 19 of the year	–26.06	7.11	–3.67	<0.001
week 20 of the year	–20.11	7.11	–2.83	0.005
week 21 of the year	–17.92	7.16	–2.50	0.013
week 22 of the year	–2.77	7.21	–0.38	0.701
week 23 of the year	–29.60	7.10	–4.17	<0.001
week 24 of the year	–9.21	7.21	–1.28	0.204
week 25 of the year	6.16	7.15	0.86	0.390
week 26 of the year	–1.76	7.10	–0.25	0.805
week 27 of the year	–4.45	7.10	–0.63	0.532
week 28 of the year	–8.39	7.10	–1.18	0.239
week 29 of the year	2.90	7.10	0.41	0.683
week 30 of the year	–8.50	7.10	–1.20	0.233
week 31 of the year	2.39	8.24	0.29	0.772
week 32 of the year	–13.84	7.10	–1.95	0.053
week 33 of the year	7.17	7.09	1.01	0.314
week 34 of the year	–0.70	7.09	–0.10	0.921
week 35 of the year	–1.81	7.09	–0.25	0.799
week 36 of the year	18.30	8.24	2.22	0.028
week 37 of the year	12.63	7.09	1.78	0.077
week 38 of the year	30.15	7.09	4.25	<0.001
week 39 of the year	12.61	7.09	1.78	0.077
week 40 of the year	9.19	7.09	1.30	0.197
week 41 of the year	12.68	7.09	1.79	0.076
week 42 of the year	10.41	7.09	1.47	0.144
week 43 of the year	4.45	7.09	0.63	0.531
week 44 of the year	12.88	7.09	1.82	0.071
week 45 of the year	19.99	8.24	2.43	0.016
week 46 of the year	2.03	7.09	0.29	0.775
week 47 of the year	9.56	7.08	1.35	0.179
week 48 of the year	3.97	7.08	0.56	0.576
week 49 of the year	–2.51	7.06	–0.36	0.723
week 50 of the year	0.77	7.02	0.11	0.913
week 51 of the year	23.01	6.80	3.39	<0.001
week 52 of the year	14.78	6.55	2.26	0.025
holiday in week	–7.53	4.20	–1.79	0.075

Non-residential burglary in Los Angeles, CA	estimate	SE	statistic	p value
intercept	123.01	7.67	16.04	<0.001
AR(1)	0.90	0.06	15.72	<0.001
MA(1)	–0.72	0.09	–7.81	<0.001
SAR(1)	–0.34	0.08	–4.34	<0.001
linear trend	–0.03	0.04	–0.66	0.508
week 2 of the year	–16.49	7.40	–2.23	0.027
week 3 of the year	2.37	7.54	0.31	0.753

Non-residential burglary in Los Angeles, CA	estimate	SE	statistic	p value
week 4 of the year	13.18	8.46	1.56	0.121
week 5 of the year	6.79	7.85	0.86	0.389
week 6 of the year	–19.22	7.93	–2.42	0.016
week 7 of the year	4.33	8.00	0.54	0.589
week 8 of the year	32.64	8.07	4.04	<0.001
week 9 of the year	18.30	9.30	1.97	0.051
week 10 of the year	11.53	8.70	1.33	0.187
week 11 of the year	6.48	7.88	0.82	0.412
week 12 of the year	–0.35	8.41	–0.04	0.966
week 13 of the year	–7.35	8.46	–0.87	0.386
week 14 of the year	–0.80	8.46	–0.09	0.924
week 15 of the year	1.56	8.50	0.18	0.855
week 16 of the year	–6.70	8.57	–0.78	0.436
week 17 of the year	–12.81	9.26	–1.38	0.169
week 18 of the year	–2.22	8.59	–0.26	0.797
week 19 of the year	–16.40	8.60	–1.91	0.058
week 20 of the year	5.76	8.61	0.67	0.505
week 21 of the year	–0.93	8.71	–0.11	0.915
week 22 of the year	17.28	8.73	1.98	0.049
week 23 of the year	–12.37	8.64	–1.43	0.154
week 24 of the year	6.99	8.76	0.80	0.426
week 25 of the year	1.24	8.71	0.14	0.887
week 26 of the year	–4.65	8.67	–0.54	0.592
week 27 of the year	2.98	8.66	0.34	0.732
week 28 of the year	2.81	8.65	0.32	0.746
week 29 of the year	–3.40	8.66	–0.39	0.695
week 30 of the year	10.95	8.64	1.27	0.207
week 31 of the year	–0.33	9.75	–0.03	0.973
week 32 of the year	–10.01	8.60	–1.16	0.246
week 33 of the year	–9.37	8.60	–1.09	0.277
week 34 of the year	–2.51	8.59	–0.29	0.771
week 35 of the year	0.53	8.56	0.06	0.950
week 36 of the year	–21.66	9.70	–2.23	0.027
week 37 of the year	–5.70	8.51	–0.67	0.505
week 38 of the year	1.55	8.48	0.18	0.856
week 39 of the year	–12.24	8.44	–1.45	0.149
week 40 of the year	11.13	8.43	1.32	0.189
week 41 of the year	–9.43	8.39	–1.12	0.263
week 42 of the year	–0.39	8.34	–0.05	0.963
week 43 of the year	–12.11	8.30	–1.46	0.147
week 44 of the year	0.06	8.23	0.01	0.994
week 45 of the year	–15.36	9.34	–1.65	0.102
week 46 of the year	–0.94	8.07	–0.12	0.907
week 47 of the year	–0.80	7.99	–0.10	0.920
week 48 of the year	22.38	7.90	2.83	0.005
week 49 of the year	–5.85	7.83	–0.75	0.456
week 50 of the year	–1.51	7.68	–0.20	0.844
week 51 of the year	1.88	7.54	0.25	0.804
week 52 of the year	6.98	7.40	0.94	0.347
holiday in week	0.77	4.53	0.17	0.866

Non-residential burglary in Louisville, KY	estimate	SE	statistic	p value
intercept	38.45	2.59	14.87	<0.001
AR(1)	0.62	0.23	2.68	0.008
MA(1)	–0.45	0.26	–1.72	0.088
SAR(1)	–0.33	0.08	–4.33	<0.001
linear trend	–0.02	0.01	–2.47	0.015
week 2 of the year	–1.99	3.01	–0.66	0.511
week 3 of the year	–5.11	3.14	–1.63	0.106
week 4 of the year	–5.93	3.53	–1.68	0.095
week 5 of the year	5.65	3.29	1.72	0.088
week 6 of the year	–17.07	3.30	–5.18	<0.001
week 7 of the year	–12.08	3.31	–3.65	<0.001
week 8 of the year	–12.82	3.32	–3.86	<0.001
week 9 of the year	–13.54	3.78	–3.58	<0.001
week 10 of the year	–6.21	3.50	–1.77	0.078
week 11 of the year	–11.97	3.15	–3.80	<0.001
week 12 of the year	–11.72	3.34	–3.50	<0.001
week 13 of the year	–10.69	3.36	–3.18	0.002
week 14 of the year	–7.89	3.35	–2.36	0.020
week 15 of the year	–6.68	3.35	–1.99	0.048
week 16 of the year	–13.89	3.37	–4.12	<0.001
week 17 of the year	–15.85	3.65	–4.35	<0.001
week 18 of the year	–12.26	3.35	–3.66	<0.001
week 19 of the year	–8.95	3.35	–2.67	0.008
week 20 of the year	–12.92	3.35	–3.86	<0.001
week 21 of the year	–15.38	3.37	–4.56	<0.001
week 22 of the year	–7.67	3.39	–2.26	0.025
week 23 of the year	–10.20	3.35	–3.05	0.003
week 24 of the year	–10.04	3.40	–2.96	0.004
week 25 of the year	–7.22	3.37	–2.14	0.034
week 26 of the year	–5.64	3.35	–1.68	0.095
week 27 of the year	1.03	3.35	0.31	0.759
week 28 of the year	–3.88	3.35	–1.16	0.249
week 29 of the year	–1.44	3.35	–0.43	0.668
week 30 of the year	5.09	3.35	1.52	0.130
week 31 of the year	–3.86	3.82	–1.01	0.314
week 32 of the year	–4.87	3.34	–1.46	0.147
week 33 of the year	–1.85	3.34	–0.55	0.582
week 34 of the year	–3.33	3.34	–0.99	0.322
week 35 of the year	–2.74	3.34	–0.82	0.414
week 36 of the year	–8.25	3.82	–2.16	0.033
week 37 of the year	0.74	3.34	0.22	0.825
week 38 of the year	0.33	3.34	0.10	0.923
week 39 of the year	1.42	3.34	0.43	0.671
week 40 of the year	2.59	3.34	0.77	0.440
week 41 of the year	–5.96	3.34	–1.78	0.076
week 42 of the year	1.55	3.34	0.47	0.642
week 43 of the year	–7.99	3.34	–2.40	0.018
week 44 of the year	–1.04	3.33	–0.31	0.755
week 45 of the year	–12.20	3.81	–3.21	0.002
week 46 of the year	–5.01	3.33	–1.51	0.134
week 47 of the year	–7.84	3.31	–2.37	0.019
week 48 of the year	–3.03	3.29	–0.92	0.360

Non-residential burglary in Louisville, KY	estimate	SE	statistic	p value
week 49 of the year	–5.02	3.27	–1.54	0.127
week 50 of the year	–0.28	3.22	–0.09	0.932
week 51 of the year	4.17	3.14	1.33	0.187
week 52 of the year	–7.81	3.01	–2.59	0.010
holiday in week	3.25	1.84	1.76	0.080

Non-residential burglary in Memphis, TN	estimate	SE	statistic	p value
intercept	46.40	4.35	10.67	<0.001
AR(1)	0.91	0.05	17.34	<0.001
MA(1)	–0.64	0.09	–6.81	<0.001
SAR(1)	–0.42	0.07	–5.74	<0.001
linear trend	–0.01	0.02	–0.23	0.820
week 2 of the year	–5.71	3.36	–1.70	0.092
week 3 of the year	–9.23	3.49	–2.65	0.009
week 4 of the year	–2.03	3.98	–0.51	0.610
week 5 of the year	–0.55	3.71	–0.15	0.882
week 6 of the year	–6.08	3.77	–1.61	0.109
week 7 of the year	–6.52	3.84	–1.70	0.092
week 8 of the year	–6.81	3.90	–1.75	0.083
week 9 of the year	–4.33	4.50	–0.96	0.338
week 10 of the year	–8.78	4.29	–2.05	0.042
week 11 of the year	–15.30	3.90	–3.92	<0.001
week 12 of the year	–11.55	4.16	–2.77	0.006
week 13 of the year	–13.56	4.20	–3.23	0.001
week 14 of the year	–16.15	4.21	–3.83	<0.001
week 15 of the year	–11.48	4.25	–2.70	0.008
week 16 of the year	–13.09	4.29	–3.05	0.003
week 17 of the year	–15.10	4.62	–3.27	0.001
week 18 of the year	–20.25	4.32	–4.69	<0.001
week 19 of the year	–26.59	4.33	–6.14	<0.001
week 20 of the year	–18.96	4.35	–4.36	<0.001
week 21 of the year	–15.42	4.38	–3.52	<0.001
week 22 of the year	–7.35	4.41	–1.67	0.098
week 23 of the year	–13.15	4.37	–3.01	0.003
week 24 of the year	–3.84	4.43	–0.87	0.387
week 25 of the year	–6.80	4.40	–1.54	0.124
week 26 of the year	–4.41	4.38	–1.01	0.315
week 27 of the year	–0.35	4.38	–0.08	0.936
week 28 of the year	–4.79	4.37	–1.10	0.275
week 29 of the year	–0.30	4.37	–0.07	0.945
week 30 of the year	4.41	4.36	1.01	0.313
week 31 of the year	13.45	4.87	2.76	0.006
week 32 of the year	5.06	4.34	1.17	0.246
week 33 of the year	8.51	4.33	1.97	0.051
week 34 of the year	12.56	4.32	2.91	0.004
week 35 of the year	0.47	4.30	0.11	0.912
week 36 of the year	9.29	4.81	1.93	0.055
week 37 of the year	5.54	4.26	1.30	0.196
week 38 of the year	6.03	4.24	1.42	0.157
week 39 of the year	9.73	4.21	2.31	0.022
week 40 of the year	8.04	4.18	1.92	0.056

Non-residential burglary in Memphis, TN	estimate	SE	statistic	p value
week 41 of the year	7.15	4.15	1.72	0.087
week 42 of the year	3.29	4.11	0.80	0.425
week 43 of the year	1.84	4.07	0.45	0.652
week 44 of the year	–4.59	4.02	–1.14	0.255
week 45 of the year	7.62	4.53	1.68	0.095
week 46 of the year	3.25	3.91	0.83	0.407
week 47 of the year	–0.91	3.85	–0.24	0.813
week 48 of the year	–6.78	3.77	–1.80	0.074
week 49 of the year	3.27	3.69	0.89	0.376
week 50 of the year	0.15	3.60	0.04	0.968
week 51 of the year	–3.03	3.49	–0.87	0.387
week 52 of the year	–4.53	3.36	–1.35	0.180
holiday in week	–2.94	2.17	–1.35	0.178

Non-residential burglary in Minneapolis, MN	estimate	SE	statistic	p value
intercept	12.87	1.64	7.85	<0.001
AR(1)	0.82	0.10	7.95	<0.001
MA(1)	–0.68	0.13	–5.19	<0.001
SAR(1)	–0.34	0.08	–4.34	<0.001
linear trend	0.02	0.01	3.37	<0.001
week 2 of the year	–1.09	1.85	–0.59	0.559
week 3 of the year	–2.46	1.89	–1.30	0.194
week 4 of the year	3.29	2.11	1.56	0.121
week 5 of the year	–4.18	1.96	–2.14	0.034
week 6 of the year	–3.52	1.96	–1.79	0.075
week 7 of the year	–7.18	1.98	–3.63	<0.001
week 8 of the year	–3.48	1.99	–1.75	0.082
week 9 of the year	0.60	2.29	0.26	0.793
week 10 of the year	–0.62	2.13	–0.29	0.770
week 11 of the year	–3.10	1.91	–1.62	0.108
week 12 of the year	–2.07	2.04	–1.02	0.310
week 13 of the year	–3.28	2.05	–1.60	0.111
week 14 of the year	–1.26	2.04	–0.61	0.540
week 15 of the year	–8.55	2.05	–4.17	<0.001
week 16 of the year	–1.97	2.06	–0.95	0.341
week 17 of the year	–1.03	2.23	–0.46	0.646
week 18 of the year	–4.89	2.06	–2.37	0.019
week 19 of the year	–7.56	2.06	–3.68	<0.001
week 20 of the year	–4.43	2.07	–2.14	0.034
week 21 of the year	–2.07	2.07	–1.00	0.318
week 22 of the year	–1.96	2.08	–0.94	0.346
week 23 of the year	–6.22	2.05	–3.03	0.003
week 24 of the year	–5.28	2.08	–2.53	0.012
week 25 of the year	–4.65	2.07	–2.25	0.026
week 26 of the year	0.87	2.06	0.42	0.673
week 27 of the year	–5.23	2.05	–2.55	0.012
week 28 of the year	–2.96	2.05	–1.44	0.151
week 29 of the year	–0.77	2.05	–0.38	0.708
week 30 of the year	–1.15	2.05	–0.56	0.577
week 31 of the year	–3.79	2.34	–1.62	0.108
week 32 of the year	–0.19	2.05	–0.09	0.925

Non-residential burglary in Minneapolis, MN	estimate	SE	statistic	p value
week 33 of the year	–1.36	2.05	–0.66	0.509
week 34 of the year	3.56	2.06	1.73	0.086
week 35 of the year	1.03	2.05	0.50	0.616
week 36 of the year	1.68	2.34	0.72	0.475
week 37 of the year	–5.01	2.05	–2.44	0.016
week 38 of the year	–3.75	2.04	–1.84	0.068
week 39 of the year	–3.12	2.04	–1.53	0.128
week 40 of the year	0.28	2.04	0.14	0.889
week 41 of the year	–1.96	2.03	–0.96	0.336
week 42 of the year	1.03	2.03	0.51	0.613
week 43 of the year	0.29	2.02	0.14	0.885
week 44 of the year	1.35	2.02	0.67	0.506
week 45 of the year	0.55	2.30	0.24	0.811
week 46 of the year	1.87	2.00	0.94	0.350
week 47 of the year	2.35	1.99	1.18	0.238
week 48 of the year	–0.17	1.97	–0.09	0.930
week 49 of the year	–3.77	1.95	–1.93	0.055
week 50 of the year	–3.51	1.92	–1.83	0.069
week 51 of the year	–3.81	1.89	–2.02	0.045
week 52 of the year	–3.62	1.85	–1.96	0.052
holiday in week	–1.81	1.13	–1.60	0.112

Non-residential burglary in Montgomery County, MD	estimate	SE	statistic	p value
intercept	6.01	1.37	4.39	<0.001
SAR(1)	–0.53	0.08	–6.60	<0.001
linear trend	0.01	0.00	1.14	0.255
week 2 of the year	–0.13	1.78	–0.07	0.941
week 3 of the year	–0.73	1.78	–0.41	0.683
week 4 of the year	–6.90	2.20	–3.13	0.002
week 5 of the year	8.41	1.84	4.57	<0.001
week 6 of the year	0.87	1.78	0.49	0.626
week 7 of the year	–1.84	1.78	–1.03	0.303
week 8 of the year	0.27	1.78	0.15	0.881
week 9 of the year	–1.97	2.40	–0.82	0.414
week 10 of the year	–6.56	2.40	–2.73	0.007
week 11 of the year	–2.98	1.78	–1.67	0.097
week 12 of the year	–3.08	1.96	–1.57	0.118
week 13 of the year	–1.44	2.02	–0.71	0.476
week 14 of the year	0.33	1.97	0.17	0.866
week 15 of the year	–1.15	1.97	–0.59	0.559
week 16 of the year	0.27	1.97	0.14	0.889
week 17 of the year	–4.37	2.54	–1.72	0.088
week 18 of the year	2.45	1.97	1.24	0.216
week 19 of the year	0.98	1.97	0.50	0.621
week 20 of the year	–1.43	1.97	–0.73	0.468
week 21 of the year	–1.45	1.97	–0.74	0.462
week 22 of the year	–4.69	2.09	–2.24	0.027
week 23 of the year	–1.48	1.97	–0.75	0.454
week 24 of the year	–1.95	2.02	–0.96	0.337
week 25 of the year	1.17	2.02	0.58	0.564
week 26 of the year	–1.18	1.97	–0.60	0.550

Non-residential burglary in Montgomery County, MD	estimate	SE	statistic	p value
week 27 of the year	0.79	1.97	0.40	0.690
week 28 of the year	–3.50	1.97	–1.78	0.078
week 29 of the year	3.52	1.97	1.79	0.075
week 30 of the year	–1.36	1.97	–0.69	0.490
week 31 of the year	–4.60	2.54	–1.81	0.073
week 32 of the year	–1.64	1.97	–0.83	0.406
week 33 of the year	3.07	1.97	1.56	0.121
week 34 of the year	3.32	1.97	1.68	0.095
week 35 of the year	–1.12	1.78	–0.63	0.532
week 36 of the year	–3.58	2.41	–1.49	0.140
week 37 of the year	0.98	1.78	0.55	0.585
week 38 of the year	1.64	1.79	0.92	0.362
week 39 of the year	2.85	1.78	1.60	0.113
week 40 of the year	2.04	1.78	1.15	0.254
week 41 of the year	–0.06	1.78	–0.03	0.975
week 42 of the year	2.95	1.78	1.66	0.100
week 43 of the year	–0.68	1.78	–0.38	0.703
week 44 of the year	–0.48	1.78	–0.27	0.790
week 45 of the year	–7.08	2.40	–2.95	0.004
week 46 of the year	2.43	1.78	1.36	0.175
week 47 of the year	–0.61	1.79	–0.34	0.734
week 48 of the year	0.28	1.79	0.16	0.874
week 49 of the year	0.89	1.78	0.50	0.617
week 50 of the year	2.17	1.79	1.21	0.227
week 51 of the year	2.91	1.78	1.63	0.105
week 52 of the year	2.99	1.78	1.68	0.095
holiday in week	5.80	1.61	3.61	<0.001

Non-residential burglary in Philadelphia, PA	estimate	SE	statistic	p value
intercept	25.65	2.68	9.56	<0.001
AR(1)	0.29	0.07	3.98	<0.001
AR(2)	0.07	0.08	0.83	0.406
AR(3)	–0.18	0.09	–1.96	0.051
AR(4)	0.16	0.10	1.50	0.135
AR(5)	–0.04	0.10	–0.43	0.667
SAR(1)	–0.37	0.09	–4.14	<0.001
linear trend	0.00	0.01	–0.40	0.691
week 2 of the year	–1.13	3.00	–0.38	0.707
week 3 of the year	–0.83	3.27	–0.25	0.801
week 4 of the year	–0.97	4.15	–0.23	0.816
week 5 of the year	0.77	3.37	0.23	0.820
week 6 of the year	1.90	3.52	0.54	0.589
week 7 of the year	3.12	3.49	0.89	0.373
week 8 of the year	11.30	3.68	3.07	0.003
week 9 of the year	2.79	4.05	0.69	0.493
week 10 of the year	–2.24	3.77	–0.59	0.553
week 11 of the year	–1.10	3.28	–0.34	0.738
week 12 of the year	4.24	3.60	1.18	0.240
week 13 of the year	–2.27	3.50	–0.65	0.518
week 14 of the year	–6.31	3.51	–1.80	0.074
week 15 of the year	1.77	3.52	0.50	0.617

Non-residential burglary in Philadelphia, PA	estimate	SE	statistic	p value
week 16 of the year	–1.77	3.52	–0.50	0.615
week 17 of the year	–2.34	3.91	–0.60	0.551
week 18 of the year	–1.17	3.50	–0.33	0.738
week 19 of the year	–7.37	3.51	–2.10	0.037
week 20 of the year	1.02	3.50	0.29	0.770
week 21 of the year	0.90	3.54	0.26	0.799
week 22 of the year	–3.16	3.55	–0.89	0.375
week 23 of the year	–6.21	3.50	–1.77	0.078
week 24 of the year	–4.18	3.56	–1.17	0.242
week 25 of the year	–6.56	3.53	–1.86	0.065
week 26 of the year	4.59	3.50	1.31	0.191
week 27 of the year	2.40	3.50	0.69	0.493
week 28 of the year	–2.93	3.49	–0.84	0.403
week 29 of the year	–0.15	3.49	–0.04	0.965
week 30 of the year	1.37	3.49	0.39	0.696
week 31 of the year	3.27	4.11	0.80	0.428
week 32 of the year	0.11	3.49	0.03	0.975
week 33 of the year	0.35	3.49	0.10	0.920
week 34 of the year	5.80	3.49	1.66	0.099
week 35 of the year	0.31	3.49	0.09	0.929
week 36 of the year	–0.66	4.11	–0.16	0.872
week 37 of the year	2.00	3.49	0.57	0.566
week 38 of the year	9.01	3.49	2.58	0.011
week 39 of the year	0.86	3.49	0.25	0.806
week 40 of the year	6.04	3.49	1.73	0.086
week 41 of the year	5.75	3.48	1.65	0.101
week 42 of the year	5.34	3.50	1.53	0.129
week 43 of the year	6.81	3.47	1.96	0.052
week 44 of the year	4.01	3.50	1.14	0.254
week 45 of the year	1.82	4.09	0.44	0.658
week 46 of the year	–2.09	3.53	–0.59	0.555
week 47 of the year	6.00	3.44	1.74	0.083
week 48 of the year	3.66	3.50	1.04	0.298
week 49 of the year	0.05	3.35	0.01	0.989
week 50 of the year	–1.13	3.65	–0.31	0.756
week 51 of the year	1.86	3.27	0.57	0.571
week 52 of the year	3.63	3.00	1.21	0.227
holiday in week	0.53	2.10	0.25	0.802

Non-residential burglary in Phoenix, AZ	estimate	SE	statistic	p value
intercept	53.90	3.28	16.43	<0.001
AR(1)	0.32	0.07	4.75	<0.001
AR(2)	0.26	0.07	3.80	<0.001
SAR(1)	–0.49	0.07	–6.70	<0.001
linear trend	0.00	0.01	–0.30	0.765
week 2 of the year	–2.30	2.93	–0.78	0.434
week 3 of the year	–5.30	3.03	–1.75	0.082
week 4 of the year	1.21	3.71	0.33	0.746
week 5 of the year	2.20	3.53	0.62	0.533
week 6 of the year	–2.23	3.63	–0.61	0.540
week 7 of the year	–0.44	3.69	–0.12	0.905

Non-residential burglary in Phoenix, AZ	estimate	SE	statistic	p value
week 8 of the year	–0.09	3.72	–0.02	0.982
week 9 of the year	–3.82	4.17	–0.92	0.361
week 10 of the year	–4.88	3.95	–1.23	0.219
week 11 of the year	1.64	3.61	0.45	0.651
week 12 of the year	–7.22	3.81	–1.90	0.060
week 13 of the year	–3.31	3.82	–0.87	0.387
week 14 of the year	0.77	3.84	0.20	0.841
week 15 of the year	–8.25	3.87	–2.13	0.035
week 16 of the year	–4.78	3.89	–1.23	0.221
week 17 of the year	–7.51	4.17	–1.80	0.074
week 18 of the year	–3.58	3.89	–0.92	0.359
week 19 of the year	–10.98	3.89	–2.82	0.005
week 20 of the year	–1.69	3.89	–0.44	0.664
week 21 of the year	3.54	3.91	0.91	0.366
week 22 of the year	4.03	3.93	1.03	0.306
week 23 of the year	–7.57	3.88	–1.95	0.053
week 24 of the year	–3.05	3.93	–0.78	0.439
week 25 of the year	3.35	3.90	0.86	0.392
week 26 of the year	9.32	3.88	2.40	0.017
week 27 of the year	–4.08	3.88	–1.05	0.294
week 28 of the year	–2.66	3.88	–0.69	0.494
week 29 of the year	1.94	3.87	0.50	0.617
week 30 of the year	3.75	3.87	0.97	0.334
week 31 of the year	–2.41	4.36	–0.55	0.581
week 32 of the year	–0.14	3.87	–0.04	0.972
week 33 of the year	5.78	3.88	1.49	0.138
week 34 of the year	3.68	3.87	0.95	0.343
week 35 of the year	0.48	3.87	0.12	0.901
week 36 of the year	–3.14	4.32	–0.73	0.468
week 37 of the year	3.14	3.86	0.81	0.417
week 38 of the year	–3.22	3.86	–0.83	0.406
week 39 of the year	–0.72	3.86	–0.19	0.853
week 40 of the year	9.77	3.85	2.54	0.012
week 41 of the year	–6.59	3.85	–1.71	0.089
week 42 of the year	–7.01	3.84	–1.83	0.070
week 43 of the year	2.78	3.83	0.73	0.468
week 44 of the year	–0.80	3.81	–0.21	0.833
week 45 of the year	–11.30	4.25	–2.66	0.009
week 46 of the year	–1.17	3.76	–0.31	0.755
week 47 of the year	–6.96	3.71	–1.88	0.062
week 48 of the year	4.60	3.63	1.27	0.207
week 49 of the year	–3.87	3.51	–1.10	0.271
week 50 of the year	4.60	3.38	1.36	0.176
week 51 of the year	1.72	3.01	0.57	0.570
week 52 of the year	–1.88	2.93	–0.64	0.522
holiday in week	5.90	1.93	3.06	0.003

Non-residential burglary in Sacramento, CA	estimate	SE	statistic	p value
intercept	12.96	1.71	7.58	<0.001
AR(1)	0.76	0.11	6.82	<0.001
MA(1)	–0.61	0.13	–4.71	<0.001

Non-residential burglary in Sacramento, CA	estimate	SE	statistic	p value
SAR(1)	–0.41	0.08	–5.35	<0.001
linear trend	0.04	0.01	6.25	<0.001
week 2 of the year	0.14	1.96	0.07	0.943
week 3 of the year	1.34	2.00	0.67	0.506
week 4 of the year	–0.78	2.27	–0.34	0.732
week 5 of the year	0.68	2.09	0.32	0.747
week 6 of the year	2.31	2.09	1.10	0.272
week 7 of the year	2.80	2.11	1.32	0.187
week 8 of the year	4.26	2.12	2.01	0.046
week 9 of the year	–1.80	2.45	–0.73	0.465
week 10 of the year	–0.57	2.28	–0.25	0.803
week 11 of the year	1.46	2.03	0.72	0.471
week 12 of the year	2.49	2.16	1.15	0.253
week 13 of the year	1.72	2.17	0.79	0.429
week 14 of the year	5.40	2.16	2.50	0.013
week 15 of the year	5.81	2.16	2.69	0.008
week 16 of the year	0.84	2.18	0.39	0.700
week 17 of the year	–3.89	2.38	–1.63	0.104
week 18 of the year	2.55	2.16	1.18	0.241
week 19 of the year	0.72	2.17	0.33	0.742
week 20 of the year	–0.59	2.17	–0.27	0.786
week 21 of the year	5.51	2.18	2.53	0.012
week 22 of the year	5.84	2.19	2.66	0.009
week 23 of the year	1.75	2.16	0.81	0.420
week 24 of the year	2.32	2.19	1.06	0.292
week 25 of the year	2.15	2.18	0.99	0.324
week 26 of the year	1.29	2.16	0.60	0.551
week 27 of the year	1.65	2.16	0.76	0.447
week 28 of the year	2.18	2.16	1.01	0.315
week 29 of the year	–2.59	2.17	–1.19	0.234
week 30 of the year	–0.81	2.16	–0.38	0.707
week 31 of the year	–1.34	2.50	–0.53	0.594
week 32 of the year	–0.03	2.17	–0.02	0.988
week 33 of the year	0.31	2.16	0.14	0.886
week 34 of the year	1.60	2.16	0.74	0.460
week 35 of the year	–2.80	2.16	–1.30	0.196
week 36 of the year	3.28	2.49	1.32	0.190
week 37 of the year	1.34	2.16	0.62	0.536
week 38 of the year	5.75	2.15	2.67	0.008
week 39 of the year	4.48	2.16	2.07	0.040
week 40 of the year	–1.12	2.15	–0.52	0.602
week 41 of the year	–1.90	2.15	–0.88	0.378
week 42 of the year	1.98	2.15	0.92	0.358
week 43 of the year	3.22	2.14	1.50	0.135
week 44 of the year	3.18	2.14	1.49	0.139
week 45 of the year	–4.32	2.47	–1.75	0.082
week 46 of the year	4.52	2.12	2.13	0.035
week 47 of the year	–3.40	2.11	–1.61	0.108
week 48 of the year	–1.17	2.10	–0.56	0.577
week 49 of the year	–1.06	2.07	–0.51	0.611
week 50 of the year	0.16	2.05	0.08	0.938
week 51 of the year	–2.98	2.00	–1.49	0.139

Non-residential burglary in Sacramento, CA	estimate	SE	statistic	p value
week 52 of the year	3.23	1.96	1.65	0.101
holiday in week	0.93	1.25	0.74	0.458

Non-residential burglary in San Francisco, CA	estimate	SE	statistic	p value
AR(1)	0.98	0.02	59.96	<0.001
MA(1)	–0.64	0.06	–11.38	<0.001
SAR(1)	–0.40	0.07	–5.53	<0.001
linear trend	0.34	0.07	5.22	<0.001
week 2 of the year	5.56	3.46	1.60	0.111
week 3 of the year	–5.76	3.63	–1.59	0.114
week 4 of the year	0.85	4.13	0.21	0.836
week 5 of the year	–2.45	3.89	–0.63	0.530
week 6 of the year	–1.54	3.99	–0.39	0.700
week 7 of the year	–4.60	4.10	–1.12	0.263
week 8 of the year	–0.74	4.19	–0.18	0.860
week 9 of the year	–6.33	4.81	–1.32	0.190
week 10 of the year	8.57	4.64	1.85	0.067
week 11 of the year	7.44	4.29	1.73	0.085
week 12 of the year	13.84	4.57	3.02	0.003
week 13 of the year	12.45	4.65	2.68	0.008
week 14 of the year	18.77	4.71	3.99	<0.001
week 15 of the year	5.97	4.77	1.25	0.213
week 16 of the year	9.99	4.85	2.06	0.041
week 17 of the year	14.24	5.17	2.76	0.007
week 18 of the year	11.70	4.93	2.37	0.019
week 19 of the year	13.11	4.98	2.63	0.009
week 20 of the year	14.84	5.02	2.96	0.004
week 21 of the year	7.56	5.07	1.49	0.138
week 22 of the year	11.13	5.12	2.18	0.031
week 23 of the year	13.12	5.10	2.57	0.011
week 24 of the year	8.21	5.16	1.59	0.113
week 25 of the year	5.92	5.15	1.15	0.252
week 26 of the year	17.50	5.14	3.40	<0.001
week 27 of the year	7.76	5.15	1.51	0.134
week 28 of the year	6.26	5.14	1.22	0.225
week 29 of the year	12.14	5.14	2.36	0.019
week 30 of the year	4.23	5.13	0.82	0.411
week 31 of the year	9.46	5.55	1.70	0.090
week 32 of the year	–0.53	5.09	–0.10	0.918
week 33 of the year	4.42	5.07	0.87	0.384
week 34 of the year	3.67	5.03	0.73	0.467
week 35 of the year	10.16	5.00	2.03	0.044
week 36 of the year	–0.38	5.42	–0.07	0.944
week 37 of the year	2.92	4.91	0.60	0.553
week 38 of the year	8.33	4.86	1.71	0.089
week 39 of the year	15.23	4.81	3.17	0.002
week 40 of the year	13.18	4.75	2.78	0.006
week 41 of the year	8.61	4.68	1.84	0.068
week 42 of the year	8.33	4.60	1.81	0.072
week 43 of the year	13.06	4.53	2.89	0.004
week 44 of the year	7.57	4.44	1.71	0.090

Non-residential burglary in San Francisco, CA	estimate	SE	statistic	p value
week 45 of the year	5.26	4.87	1.08	0.282
week 46 of the year	–0.53	4.25	–0.12	0.901
week 47 of the year	4.34	4.14	1.05	0.296
week 48 of the year	6.98	4.03	1.73	0.085
week 49 of the year	–8.06	3.90	–2.07	0.040
week 50 of the year	3.73	3.77	0.99	0.323
week 51 of the year	1.52	3.63	0.42	0.676
week 52 of the year	1.94	3.47	0.56	0.577
holiday in week	–0.72	2.21	–0.33	0.744

Theft of vehicle

Theft of vehicle in Atlanta, GA	estimate	SE	statistic	*p* value
intercept	76.26	3.26	23.42	<0.001
SAR(1)	–0.42	0.08	–5.20	<0.001
linear trend	–0.09	0.01	–10.52	<0.001
week 2 of the year	1.45	4.36	0.33	0.740
week 3 of the year	2.45	4.36	0.56	0.574
week 4 of the year	–0.64	4.92	–0.13	0.896
week 5 of the year	0.28	4.40	0.06	0.950
week 6 of the year	1.92	4.37	0.44	0.660
week 7 of the year	2.90	4.37	0.66	0.508
week 8 of the year	8.27	4.36	1.90	0.060
week 9 of the year	4.70	5.23	0.90	0.370
week 10 of the year	–12.03	4.55	–2.64	0.009
week 11 of the year	–7.80	4.38	–1.78	0.077
week 12 of the year	2.98	4.94	0.60	0.547
week 13 of the year	–9.59	4.42	–2.17	0.032
week 14 of the year	–4.80	4.39	–1.09	0.276
week 15 of the year	–7.64	4.37	–1.75	0.083
week 16 of the year	–12.21	4.41	–2.77	0.006
week 17 of the year	–0.74	4.93	–0.15	0.881
week 18 of the year	–17.25	4.37	–3.94	<0.001
week 19 of the year	–18.92	4.38	–4.32	<0.001
week 20 of the year	–19.88	4.38	–4.54	<0.001
week 21 of the year	–14.54	4.41	–3.29	0.001
week 22 of the year	–4.35	4.45	–0.98	0.330
week 23 of the year	–9.31	4.38	–2.13	0.035
week 24 of the year	–9.99	4.46	–2.24	0.027
week 25 of the year	–1.39	4.43	–0.31	0.754
week 26 of the year	–6.02	4.39	–1.37	0.172
week 27 of the year	0.48	4.37	0.11	0.912
week 28 of the year	–6.88	4.41	–1.56	0.120
week 29 of the year	3.86	4.38	0.88	0.379
week 30 of the year	–11.20	4.37	–2.56	0.011
week 31 of the year	–1.37	5.23	–0.26	0.793
week 32 of the year	–10.16	4.38	–2.32	0.022
week 33 of the year	0.71	4.36	0.16	0.871
week 34 of the year	1.15	4.38	0.26	0.794
week 35 of the year	–6.02	4.36	–1.38	0.169
week 36 of the year	–1.08	5.24	–0.21	0.837
week 37 of the year	5.47	4.37	1.25	0.213
week 38 of the year	–3.99	4.38	–0.91	0.364
week 39 of the year	15.93	4.38	3.64	<0.001
week 40 of the year	4.11	4.38	0.94	0.349
week 41 of the year	–0.74	4.37	–0.17	0.865
week 42 of the year	–1.54	4.37	–0.35	0.724
week 43 of the year	–5.52	4.36	–1.26	0.208
week 44 of the year	–10.19	4.39	–2.32	0.022
week 45 of the year	2.04	5.24	0.39	0.697
week 46 of the year	1.13	4.38	0.26	0.797
week 47 of the year	3.20	4.38	0.73	0.467
week 48 of the year	–0.56	4.36	–0.13	0.898

Theft of vehicle in Atlanta, GA	estimate	SE	statistic	*p* value
week 49 of the year	0.14	4.38	0.03	0.974
week 50 of the year	6.07	4.37	1.39	0.166
week 51 of the year	–2.78	4.36	–0.64	0.525
week 52 of the year	–3.42	4.36	–0.78	0.434
holiday in week	–4.68	2.87	–1.63	0.105

Theft of vehicle in Austin, TX	estimate	SE	statistic	*p* value
intercept	34.42	4.11	8.38	<0.001
AR(1)	0.96	0.03	32.21	<0.001
MA(1)	–0.80	0.06	–13.29	<0.001
SAR(1)	–0.34	0.08	–4.01	<0.001
linear trend	0.09	0.03	3.56	<0.001
week 2 of the year	–0.12	3.05	–0.04	0.969
week 3 of the year	–4.90	3.08	–1.59	0.114
week 4 of the year	2.65	3.47	0.76	0.447
week 5 of the year	0.81	3.17	0.25	0.799
week 6 of the year	–2.41	3.18	–0.76	0.450
week 7 of the year	3.26	3.20	1.02	0.310
week 8 of the year	5.67	3.23	1.75	0.081
week 9 of the year	–1.51	3.79	–0.40	0.691
week 10 of the year	2.58	3.54	0.73	0.467
week 11 of the year	6.04	3.14	1.92	0.056
week 12 of the year	–3.69	3.37	–1.09	0.275
week 13 of the year	–1.15	3.40	–0.34	0.735
week 14 of the year	–0.88	3.39	–0.26	0.795
week 15 of the year	–6.18	3.40	–1.82	0.071
week 16 of the year	3.81	3.44	1.11	0.269
week 17 of the year	3.05	3.72	0.82	0.413
week 18 of the year	2.50	3.43	0.73	0.467
week 19 of the year	5.99	3.44	1.74	0.084
week 20 of the year	3.34	3.45	0.97	0.334
week 21 of the year	–1.50	3.47	–0.43	0.666
week 22 of the year	–0.46	3.50	–0.13	0.895
week 23 of the year	0.32	3.46	0.09	0.927
week 24 of the year	–4.33	3.50	–1.24	0.218
week 25 of the year	1.88	3.48	0.54	0.590
week 26 of the year	–4.85	3.46	–1.40	0.163
week 27 of the year	–1.02	3.46	–0.29	0.769
week 28 of the year	–1.67	3.46	–0.48	0.631
week 29 of the year	–0.89	3.46	–0.26	0.797
week 30 of the year	–5.81	3.45	–1.69	0.094
week 31 of the year	3.23	3.93	0.82	0.412
week 32 of the year	10.26	3.43	2.99	0.003
week 33 of the year	8.47	3.43	2.47	0.015
week 34 of the year	4.60	3.42	1.35	0.180
week 35 of the year	0.39	3.41	0.11	0.910
week 36 of the year	2.71	3.90	0.69	0.489
week 37 of the year	4.89	3.39	1.45	0.150
week 38 of the year	3.25	3.37	0.96	0.336
week 39 of the year	–1.90	3.36	–0.56	0.573
week 40 of the year	4.17	3.34	1.25	0.214

Theft of vehicle in Austin, TX	estimate	SE	statistic	p value
week 41 of the year	–0.67	3.33	–0.20	0.840
week 42 of the year	2.47	3.31	0.75	0.456
week 43 of the year	–4.11	3.29	–1.25	0.214
week 44 of the year	–0.61	3.28	–0.19	0.852
week 45 of the year	6.79	3.79	1.79	0.076
week 46 of the year	3.40	3.23	1.05	0.293
week 47 of the year	5.27	3.20	1.65	0.102
week 48 of the year	3.80	3.18	1.20	0.233
week 49 of the year	5.57	3.15	1.77	0.079
week 50 of the year	8.53	3.12	2.74	0.007
week 51 of the year	–1.96	3.08	–0.64	0.525
week 52 of the year	1.28	3.05	0.42	0.676
holiday in week	–2.03	1.91	–1.07	0.288

Theft of vehicle in Baltimore, MD	estimate	SE	statistic	p value
intercept	105.84	5.12	20.65	<0.001
AR(1)	0.31	0.07	4.66	<0.001
AR(2)	0.26	0.07	3.58	<0.001
SAR(1)	–0.26	0.09	–3.01	0.003
linear trend	–0.13	0.02	–5.74	<0.001
week 2 of the year	–5.91	4.69	–1.26	0.210
week 3 of the year	0.65	4.82	0.13	0.893
week 4 of the year	–11.87	5.81	–2.04	0.043
week 5 of the year	–11.11	5.64	–1.97	0.051
week 6 of the year	–12.01	5.80	–2.07	0.040
week 7 of the year	4.84	5.91	0.82	0.414
week 8 of the year	–3.49	5.96	–0.59	0.559
week 9 of the year	–1.70	6.53	–0.26	0.795
week 10 of the year	–2.97	6.20	–0.48	0.633
week 11 of the year	13.04	5.82	2.24	0.026
week 12 of the year	–11.13	6.04	–1.84	0.067
week 13 of the year	–11.61	6.18	–1.88	0.062
week 14 of the year	–10.06	6.15	–1.64	0.104
week 15 of the year	–13.14	6.19	–2.12	0.035
week 16 of the year	–19.18	6.22	–3.08	0.002
week 17 of the year	–34.40	6.56	–5.25	<0.001
week 18 of the year	–29.25	6.21	–4.71	<0.001
week 19 of the year	–26.05	6.21	–4.20	<0.001
week 20 of the year	–26.77	6.22	–4.30	<0.001
week 21 of the year	–13.82	6.23	–2.22	0.028
week 22 of the year	–25.65	6.25	–4.10	<0.001
week 23 of the year	–14.36	6.20	–2.32	0.022
week 24 of the year	–18.58	6.25	–2.97	0.003
week 25 of the year	–13.19	6.23	–2.12	0.036
week 26 of the year	–20.07	6.19	–3.24	0.001
week 27 of the year	–13.72	6.19	–2.22	0.028
week 28 of the year	–22.45	6.19	–3.63	<0.001
week 29 of the year	–19.11	6.19	–3.09	0.002
week 30 of the year	–7.82	6.19	–1.26	0.208
week 31 of the year	–20.45	6.77	–3.02	0.003
week 32 of the year	–12.52	6.18	–2.02	0.045

Theft of vehicle in Baltimore, MD	estimate	SE	statistic	p value
week 33 of the year	–0.20	6.18	–0.03	0.975
week 34 of the year	–4.47	6.18	–0.72	0.471
week 35 of the year	–9.06	6.18	–1.47	0.144
week 36 of the year	–12.57	6.76	–1.86	0.065
week 37 of the year	–15.59	6.17	–2.53	0.013
week 38 of the year	4.47	6.17	0.72	0.470
week 39 of the year	–3.37	6.17	–0.55	0.586
week 40 of the year	–1.78	6.16	–0.29	0.773
week 41 of the year	–14.34	6.15	–2.33	0.021
week 42 of the year	–9.63	6.13	–1.57	0.118
week 43 of the year	–0.81	6.12	–0.13	0.895
week 44 of the year	–11.83	6.09	–1.94	0.054
week 45 of the year	–20.39	6.65	–3.07	0.003
week 46 of the year	–9.16	6.00	–1.53	0.129
week 47 of the year	–1.42	5.92	–0.24	0.811
week 48 of the year	–8.03	5.81	–1.38	0.169
week 49 of the year	–4.19	5.61	–0.75	0.457
week 50 of the year	–11.83	5.41	–2.19	0.030
week 51 of the year	–6.44	4.83	–1.33	0.184
week 52 of the year	3.09	4.69	0.66	0.510
holiday in week	4.65	2.74	1.70	0.092

Theft of vehicle in Boston, MA	estimate	SE	statistic	p value
intercept	32.99	2.30	14.34	<0.001
MA(1)	0.16	0.07	2.18	0.031
SAR(1)	–0.49	0.08	–6.47	<0.001
linear trend	–0.04	0.01	–5.83	<0.001
week 2 of the year	–0.26	2.87	–0.09	0.929
week 3 of the year	–5.35	3.12	–1.71	0.089
week 4 of the year	–11.60	3.66	–3.17	0.002
week 5 of the year	–0.69	3.13	–0.22	0.826
week 6 of the year	–0.54	3.13	–0.17	0.864
week 7 of the year	–5.78	3.13	–1.85	0.067
week 8 of the year	–10.59	3.12	–3.39	<0.001
week 9 of the year	–4.95	3.63	–1.37	0.174
week 10 of the year	–6.27	3.26	–1.92	0.057
week 11 of the year	–7.31	2.88	–2.53	0.012
week 12 of the year	–5.89	3.26	–1.81	0.073
week 13 of the year	–6.26	2.91	–2.15	0.033
week 14 of the year	–9.89	2.88	–3.43	<0.001
week 15 of the year	–7.76	2.88	–2.69	0.008
week 16 of the year	–10.04	2.91	–3.45	<0.001
week 17 of the year	–9.29	3.26	–2.85	0.005
week 18 of the year	–9.24	2.88	–3.21	0.002
week 19 of the year	–15.09	2.88	–5.24	<0.001
week 20 of the year	–9.05	2.88	–3.14	0.002
week 21 of the year	–11.45	2.91	–3.94	<0.001
week 22 of the year	–7.03	2.94	–2.39	0.018
week 23 of the year	–5.24	2.88	–1.82	0.071
week 24 of the year	–9.05	2.94	–3.08	0.002
week 25 of the year	–7.59	2.91	–2.61	0.010

Theft of vehicle in Boston, MA	estimate	SE	statistic	*p* value
week 26 of the year	–5.31	2.88	–1.84	0.068
week 27 of the year	–3.79	2.88	–1.32	0.190
week 28 of the year	–3.95	2.88	–1.37	0.173
week 29 of the year	–2.11	2.88	–0.73	0.464
week 30 of the year	2.72	2.88	0.94	0.347
week 31 of the year	0.62	3.45	0.18	0.858
week 32 of the year	0.00	2.88	0.00	0.999
week 33 of the year	8.62	2.89	2.98	0.003
week 34 of the year	3.58	2.88	1.24	0.216
week 35 of the year	7.61	2.89	2.63	0.009
week 36 of the year	8.51	3.45	2.47	0.015
week 37 of the year	1.70	2.88	0.59	0.557
week 38 of the year	3.63	2.88	1.26	0.211
week 39 of the year	–0.03	2.88	–0.01	0.991
week 40 of the year	9.70	2.89	3.36	0.001
week 41 of the year	7.55	2.88	2.62	0.010
week 42 of the year	2.20	2.88	0.76	0.446
week 43 of the year	3.53	2.88	1.23	0.222
week 44 of the year	–1.34	2.88	–0.46	0.644
week 45 of the year	2.56	3.45	0.74	0.459
week 46 of the year	–1.25	2.88	–0.43	0.665
week 47 of the year	3.38	2.88	1.17	0.243
week 48 of the year	1.11	2.89	0.38	0.701
week 49 of the year	–7.45	2.89	–2.58	0.011
week 50 of the year	–7.02	2.89	–2.43	0.016
week 51 of the year	–2.16	3.12	–0.69	0.490
week 52 of the year	–0.18	2.87	–0.06	0.949
holiday in week	0.15	1.90	0.08	0.939

Theft of vehicle in Chicago, IL	estimate	SE	statistic	*p* value
intercept	498.06	19.25	25.87	<0.001
AR(1)	0.42	0.07	5.89	<0.001
AR(2)	0.13	0.07	1.79	0.075
SAR(1)	–0.30	0.08	–3.70	<0.001
linear trend	–0.65	0.08	–7.96	<0.001
week 2 of the year	–7.95	16.76	–0.47	0.636
week 3 of the year	–11.99	19.02	–0.63	0.530
week 4 of the year	–35.92	22.21	–1.62	0.108
week 5 of the year	8.52	21.86	0.39	0.697
week 6 of the year	46.70	22.38	2.09	0.039
week 7 of the year	0.35	22.68	0.02	0.988
week 8 of the year	23.03	22.83	1.01	0.315
week 9 of the year	–17.46	24.74	–0.71	0.482
week 10 of the year	15.88	23.53	0.68	0.501
week 11 of the year	–29.37	21.94	–1.34	0.183
week 12 of the year	–18.44	22.83	–0.81	0.420
week 13 of the year	–27.59	23.09	–1.19	0.234
week 14 of the year	–13.97	23.26	–0.60	0.549
week 15 of the year	–78.32	23.37	–3.35	0.001
week 16 of the year	–72.51	23.51	–3.08	0.002
week 17 of the year	–98.95	24.72	–4.00	<0.001

Theft of vehicle in Chicago, IL	estimate	SE	statistic	p value
week 18 of the year	–83.10	23.46	–3.54	<0.001
week 19 of the year	–111.97	23.44	–4.78	<0.001
week 20 of the year	–88.85	23.45	–3.79	<0.001
week 21 of the year	–109.44	23.54	–4.65	<0.001
week 22 of the year	–99.04	23.60	–4.20	<0.001
week 23 of the year	–87.45	23.42	–3.73	<0.001
week 24 of the year	–48.27	23.59	–2.05	0.042
week 25 of the year	–82.63	23.52	–3.51	<0.001
week 26 of the year	–68.21	23.39	–2.92	0.004
week 27 of the year	–82.56	23.39	–3.53	<0.001
week 28 of the year	–73.08	23.38	–3.13	0.002
week 29 of the year	–40.56	23.39	–1.73	0.085
week 30 of the year	–81.17	23.40	–3.47	<0.001
week 31 of the year	–49.15	25.46	–1.93	0.055
week 32 of the year	–32.66	23.35	–1.40	0.164
week 33 of the year	–19.83	23.35	–0.85	0.397
week 34 of the year	–16.70	23.34	–0.72	0.475
week 35 of the year	5.12	23.34	0.22	0.827
week 36 of the year	–19.16	25.43	–0.75	0.452
week 37 of the year	–1.75	23.35	–0.07	0.940
week 38 of the year	–6.36	23.33	–0.27	0.786
week 39 of the year	8.51	23.32	0.37	0.716
week 40 of the year	0.42	23.31	0.02	0.986
week 41 of the year	–6.58	23.28	–0.28	0.778
week 42 of the year	1.64	23.26	0.07	0.944
week 43 of the year	–12.41	23.22	–0.53	0.594
week 44 of the year	5.28	23.16	0.23	0.820
week 45 of the year	–31.01	25.20	–1.23	0.220
week 46 of the year	–33.03	22.93	–1.44	0.152
week 47 of the year	10.66	22.71	0.47	0.639
week 48 of the year	–15.35	22.36	–0.69	0.493
week 49 of the year	–0.33	21.75	–0.02	0.988
week 50 of the year	–53.30	20.80	–2.56	0.011
week 51 of the year	–32.56	19.03	–1.71	0.089
week 52 of the year	–16.76	16.74	–1.00	0.318
holiday in week	7.72	10.08	0.77	0.445

Theft of vehicle in Los Angeles, CA	estimate	SE	statistic	p value
intercept	424.33	11.56	36.69	<0.001
AR(1)	0.41	0.07	6.21	<0.001
AR(2)	–0.01	0.07	–0.11	0.915
AR(3)	0.32	0.07	4.90	<0.001
SAR(1)	–0.38	0.08	–4.67	<0.001
linear trend	–0.45	0.06	–7.15	<0.001
week 2 of the year	–29.37	8.71	–3.37	<0.001
week 3 of the year	–0.31	9.98	–0.03	0.975
week 4 of the year	–19.31	10.01	–1.93	0.056
week 5 of the year	–14.92	10.14	–1.47	0.143
week 6 of the year	–13.75	10.80	–1.27	0.205
week 7 of the year	–7.51	10.79	–0.70	0.487
week 8 of the year	8.69	11.08	0.78	0.434

Theft of vehicle in Los Angeles, CA	estimate	SE	statistic	p value
week 9 of the year	–26.03	12.43	–2.09	0.038
week 10 of the year	–42.54	11.93	–3.56	<0.001
week 11 of the year	–1.73	11.18	–0.15	0.877
week 12 of the year	–2.64	11.75	–0.22	0.822
week 13 of the year	–29.82	11.97	–2.49	0.014
week 14 of the year	–31.18	12.07	–2.58	0.011
week 15 of the year	–42.31	12.14	–3.48	<0.001
week 16 of the year	–40.97	12.31	–3.33	0.001
week 17 of the year	–66.81	13.00	–5.14	<0.001
week 18 of the year	–49.57	12.40	–4.00	<0.001
week 19 of the year	–53.30	12.45	–4.28	<0.001
week 20 of the year	–42.44	12.48	–3.40	<0.001
week 21 of the year	–31.71	12.55	–2.53	0.013
week 22 of the year	–40.59	12.61	–3.22	0.002
week 23 of the year	–33.07	12.54	–2.64	0.009
week 24 of the year	–40.91	12.63	–3.24	0.001
week 25 of the year	–48.63	12.60	–3.86	<0.001
week 26 of the year	–43.35	12.55	–3.45	<0.001
week 27 of the year	–36.81	12.54	–2.93	0.004
week 28 of the year	–39.12	12.54	–3.12	0.002
week 29 of the year	–40.90	12.53	–3.27	0.001
week 30 of the year	–13.44	12.52	–1.07	0.285
week 31 of the year	–41.46	13.53	–3.06	0.003
week 32 of the year	–49.88	12.48	–4.00	<0.001
week 33 of the year	–42.70	12.45	–3.43	<0.001
week 34 of the year	–49.78	12.43	–4.00	<0.001
week 35 of the year	–32.05	12.39	–2.59	0.011
week 36 of the year	–59.20	13.40	–4.42	<0.001
week 37 of the year	–36.06	12.30	–2.93	0.004
week 38 of the year	–32.14	12.25	–2.62	0.010
week 39 of the year	–41.53	12.18	–3.41	<0.001
week 40 of the year	–45.82	12.09	–3.79	<0.001
week 41 of the year	–34.30	12.01	–2.86	0.005
week 42 of the year	–37.46	11.90	–3.15	0.002
week 43 of the year	–33.75	11.76	–2.87	0.005
week 44 of the year	–34.29	11.61	–2.95	0.004
week 45 of the year	–72.89	12.62	–5.78	<0.001
week 46 of the year	–37.89	11.14	–3.40	<0.001
week 47 of the year	–24.99	10.86	–2.30	0.023
week 48 of the year	–26.01	10.83	–2.40	0.018
week 49 of the year	–38.48	10.10	–3.81	<0.001
week 50 of the year	–38.82	9.18	–4.23	<0.001
week 51 of the year	–14.27	9.97	–1.43	0.155
week 52 of the year	–23.34	8.72	–2.68	0.008
holiday in week	10.77	5.18	2.08	0.039

Theft of vehicle in Louisville, KY	estimate	SE	statistic	p value
intercept	86.59	3.72	23.31	<0.001
AR(1)	0.16	0.07	2.33	0.021
AR(2)	0.08	0.07	1.22	0.224
AR(3)	0.25	0.07	3.61	<0.001

Theft of vehicle in Louisville, KY	estimate	SE	statistic	p value
AR(4)	–0.08	0.07	–1.09	0.277
SAR(1)	–0.51	0.07	–7.17	<0.001
linear trend	–0.06	0.01	–3.91	<0.001
week 2 of the year	–6.10	4.16	–1.47	0.145
week 3 of the year	–6.99	4.26	–1.64	0.103
week 4 of the year	–8.87	4.49	–1.97	0.050
week 5 of the year	–0.27	4.59	–0.06	0.953
week 6 of the year	–14.22	4.51	–3.15	0.002
week 7 of the year	–3.45	4.45	–0.78	0.439
week 8 of the year	–3.36	4.61	–0.73	0.468
week 9 of the year	–17.46	5.35	–3.26	0.001
week 10 of the year	–3.19	4.90	–0.65	0.516
week 11 of the year	0.27	4.36	0.06	0.951
week 12 of the year	–2.47	4.66	–0.53	0.596
week 13 of the year	–11.18	4.64	–2.41	0.017
week 14 of the year	–10.19	4.64	–2.20	0.030
week 15 of the year	–7.24	4.64	–1.56	0.120
week 16 of the year	–7.30	4.69	–1.56	0.122
week 17 of the year	–18.26	5.13	–3.56	<0.001
week 18 of the year	–11.09	4.65	–2.38	0.018
week 19 of the year	–17.48	4.65	–3.76	<0.001
week 20 of the year	–12.29	4.65	–2.64	0.009
week 21 of the year	–10.83	4.68	–2.31	0.022
week 22 of the year	–11.05	4.74	–2.33	0.021
week 23 of the year	–6.09	4.65	–1.31	0.192
week 24 of the year	–7.00	4.72	–1.48	0.140
week 25 of the year	–5.78	4.68	–1.24	0.218
week 26 of the year	–7.02	4.65	–1.51	0.133
week 27 of the year	–6.96	4.64	–1.50	0.136
week 28 of the year	–5.68	4.64	–1.23	0.222
week 29 of the year	–7.44	4.64	–1.60	0.111
week 30 of the year	–2.40	4.65	–0.52	0.606
week 31 of the year	2.82	5.38	0.52	0.601
week 32 of the year	4.02	4.64	0.87	0.387
week 33 of the year	3.67	4.64	0.79	0.430
week 34 of the year	–3.16	4.64	–0.68	0.496
week 35 of the year	11.40	4.63	2.46	0.015
week 36 of the year	–3.22	5.38	–0.60	0.550
week 37 of the year	0.51	4.63	0.11	0.913
week 38 of the year	5.47	4.63	1.18	0.239
week 39 of the year	12.04	4.63	2.60	0.010
week 40 of the year	13.09	4.63	2.83	0.005
week 41 of the year	–0.39	4.64	–0.08	0.933
week 42 of the year	2.22	4.62	0.48	0.632
week 43 of the year	–2.84	4.63	–0.61	0.541
week 44 of the year	–0.09	4.59	–0.02	0.984
week 45 of the year	–4.29	5.34	–0.80	0.423
week 46 of the year	–2.27	4.62	–0.49	0.624
week 47 of the year	–8.03	4.47	–1.80	0.074
week 48 of the year	7.74	4.51	1.72	0.088
week 49 of the year	2.79	4.56	0.61	0.541
week 50 of the year	–2.98	3.94	–0.76	0.451

Theft of vehicle in Louisville, KY	estimate	SE	statistic	p value
week 51 of the year	5.51	4.26	1.29	0.197
week 52 of the year	–5.95	4.15	–1.43	0.154
holiday in week	3.57	2.72	1.31	0.191

Theft of vehicle in Memphis, TN	estimate	SE	statistic	p value
intercept	65.17	7.31	8.91	<0.001
AR(1)	0.17	0.54	0.31	0.756
AR(2)	0.36	0.23	1.59	0.114
AR(3)	0.23	0.19	1.19	0.237
MA(1)	0.18	0.55	0.32	0.746
SAR(1)	–0.42	0.07	–5.62	<0.001
linear trend	0.10	0.04	2.35	0.020
week 2 of the year	–4.53	4.58	–0.99	0.324
week 3 of the year	–3.89	4.66	–0.83	0.406
week 4 of the year	6.65	5.53	1.20	0.231
week 5 of the year	10.75	5.52	1.95	0.053
week 6 of the year	6.04	5.67	1.06	0.289
week 7 of the year	19.04	5.93	3.21	0.002
week 8 of the year	11.81	6.11	1.93	0.055
week 9 of the year	10.18	6.97	1.46	0.146
week 10 of the year	28.52	6.87	4.15	<0.001
week 11 of the year	4.03	6.36	0.63	0.527
week 12 of the year	25.43	6.76	3.76	<0.001
week 13 of the year	–1.01	6.77	–0.15	0.882
week 14 of the year	6.48	6.83	0.95	0.344
week 15 of the year	7.94	6.94	1.15	0.254
week 16 of the year	2.10	7.04	0.30	0.766
week 17 of the year	–3.88	7.44	–0.52	0.603
week 18 of the year	–6.91	7.13	–0.97	0.334
week 19 of the year	–12.20	7.18	–1.70	0.091
week 20 of the year	–3.32	7.21	–0.46	0.646
week 21 of the year	–2.66	7.26	–0.37	0.715
week 22 of the year	–6.18	7.31	–0.85	0.399
week 23 of the year	–7.49	7.27	–1.03	0.305
week 24 of the year	–3.24	7.34	–0.44	0.659
week 25 of the year	5.80	7.32	0.79	0.430
week 26 of the year	1.59	7.29	0.22	0.827
week 27 of the year	–8.49	7.29	–1.16	0.246
week 28 of the year	–0.22	7.29	–0.03	0.976
week 29 of the year	–2.70	7.28	–0.37	0.711
week 30 of the year	0.47	7.27	0.07	0.948
week 31 of the year	8.72	7.87	1.11	0.270
week 32 of the year	–4.93	7.23	–0.68	0.497
week 33 of the year	–8.00	7.21	–1.11	0.269
week 34 of the year	–1.23	7.19	–0.17	0.864
week 35 of the year	–4.32	7.16	–0.60	0.547
week 36 of the year	4.97	7.76	0.64	0.523
week 37 of the year	8.00	7.07	1.13	0.260
week 38 of the year	9.54	7.03	1.36	0.177
week 39 of the year	16.96	6.96	2.44	0.016
week 40 of the year	7.93	6.89	1.15	0.252

Theft of vehicle in Memphis, TN	estimate	SE	statistic	p value
week 41 of the year	6.10	6.81	0.90	0.371
week 42 of the year	8.57	6.71	1.28	0.203
week 43 of the year	0.42	6.61	0.06	0.950
week 44 of the year	0.92	6.48	0.14	0.887
week 45 of the year	4.13	7.04	0.59	0.558
week 46 of the year	1.79	6.17	0.29	0.772
week 47 of the year	12.96	5.96	2.18	0.031
week 48 of the year	6.46	5.70	1.13	0.258
week 49 of the year	8.51	5.50	1.55	0.124
week 50 of the year	2.03	4.97	0.41	0.684
week 51 of the year	1.27	4.67	0.27	0.786
week 52 of the year	–1.06	4.58	–0.23	0.817
holiday in week	–6.10	3.05	–2.00	0.047

Theft of vehicle in Minneapolis, MN	estimate	SE	statistic	p value
intercept	24.86	6.56	3.79	<0.001
AR(1)	0.97	0.03	33.14	<0.001
MA(1)	–0.86	0.06	–14.21	<0.001
linear trend	0.14	0.04	3.99	<0.001
week 2 of the year	7.35	6.41	1.15	0.253
week 3 of the year	13.19	6.44	2.05	0.042
week 4 of the year	8.80	6.87	1.28	0.202
week 5 of the year	0.97	6.54	0.15	0.882
week 6 of the year	8.48	6.53	1.30	0.196
week 7 of the year	12.33	6.55	1.88	0.062
week 8 of the year	4.92	6.58	0.75	0.455
week 9 of the year	11.13	7.28	1.53	0.128
week 10 of the year	18.41	6.80	2.71	0.008
week 11 of the year	15.17	6.34	2.39	0.018
week 12 of the year	22.24	6.62	3.36	<0.001
week 13 of the year	12.78	6.79	1.88	0.062
week 14 of the year	8.75	6.76	1.30	0.197
week 15 of the year	15.81	6.77	2.34	0.021
week 16 of the year	7.97	6.82	1.17	0.244
week 17 of the year	5.97	7.17	0.83	0.407
week 18 of the year	1.52	6.79	0.22	0.823
week 19 of the year	1.59	6.80	0.23	0.816
week 20 of the year	–0.84	6.80	–0.12	0.902
week 21 of the year	–1.68	6.85	–0.24	0.807
week 22 of the year	–4.35	6.86	–0.64	0.526
week 23 of the year	–1.62	6.81	–0.24	0.813
week 24 of the year	–1.70	6.86	–0.25	0.804
week 25 of the year	3.13	6.86	0.46	0.649
week 26 of the year	–3.14	6.81	–0.46	0.646
week 27 of the year	–3.81	6.81	–0.56	0.577
week 28 of the year	2.27	6.81	0.33	0.739
week 29 of the year	–2.65	6.81	–0.39	0.698
week 30 of the year	–4.31	6.80	–0.63	0.527
week 31 of the year	3.63	7.46	0.49	0.628
week 32 of the year	–3.65	6.79	–0.54	0.592
week 33 of the year	3.44	6.78	0.51	0.613

Theft of vehicle in Minneapolis, MN	estimate	SE	statistic	p value
week 34 of the year	6.02	6.77	0.89	0.375
week 35 of the year	2.11	6.76	0.31	0.756
week 36 of the year	3.30	7.42	0.44	0.657
week 37 of the year	–1.72	6.74	–0.25	0.799
week 38 of the year	7.87	6.73	1.17	0.244
week 39 of the year	3.96	6.71	0.59	0.556
week 40 of the year	4.05	6.70	0.60	0.546
week 41 of the year	1.39	6.68	0.21	0.836
week 42 of the year	2.73	6.66	0.41	0.683
week 43 of the year	12.07	6.64	1.82	0.071
week 44 of the year	6.16	6.62	0.93	0.354
week 45 of the year	14.61	7.29	2.00	0.047
week 46 of the year	5.09	6.58	0.77	0.440
week 47 of the year	4.94	6.55	0.75	0.452
week 48 of the year	–5.72	6.53	–0.88	0.382
week 49 of the year	2.12	6.50	0.33	0.744
week 50 of the year	–2.03	6.47	–0.31	0.754
week 51 of the year	0.06	6.44	0.01	0.993
week 52 of the year	–2.60	6.41	–0.40	0.686
holiday in week	–2.36	3.08	–0.77	0.445

Theft of vehicle in Montgomery County, MD	estimate	SE	statistic	p value
intercept	22.58	1.64	13.78	<0.001
SAR(1)	–0.39	0.09	–4.39	<0.001
linear trend	–0.01	0.01	–2.52	0.013
week 2 of the year	–3.65	2.15	–1.70	0.092
week 3 of the year	–4.67	2.15	–2.17	0.031
week 4 of the year	–1.91	2.57	–0.74	0.459
week 5 of the year	–4.99	2.19	–2.28	0.024
week 6 of the year	–7.75	2.15	–3.60	<0.001
week 7 of the year	–5.70	2.15	–2.65	0.009
week 8 of the year	–7.02	2.15	–3.26	0.001
week 9 of the year	–4.72	2.80	–1.68	0.094
week 10 of the year	–5.63	2.80	–2.01	0.047
week 11 of the year	–6.78	2.15	–3.15	0.002
week 12 of the year	–4.34	2.33	–1.86	0.065
week 13 of the year	–3.76	2.42	–1.55	0.122
week 14 of the year	–2.56	2.36	–1.09	0.280
week 15 of the year	–4.19	2.36	–1.77	0.078
week 16 of the year	–5.99	2.36	–2.54	0.012
week 17 of the year	–5.05	2.96	–1.71	0.090
week 18 of the year	–6.62	2.36	–2.81	0.006
week 19 of the year	–7.84	2.36	–3.32	0.001
week 20 of the year	–3.01	2.36	–1.28	0.204
week 21 of the year	–6.23	2.36	–2.64	0.009
week 22 of the year	–2.45	2.48	–0.99	0.324
week 23 of the year	–5.55	2.36	–2.35	0.020
week 24 of the year	–6.85	2.42	–2.83	0.005
week 25 of the year	–2.84	2.42	–1.17	0.243
week 26 of the year	–4.98	2.36	–2.11	0.037
week 27 of the year	–4.84	2.37	–2.04	0.043

Theft of vehicle in Montgomery County, MD	estimate	SE	statistic	p value
week 28 of the year	–6.32	2.36	–2.68	0.008
week 29 of the year	–4.26	2.36	–1.80	0.073
week 30 of the year	–6.23	2.36	–2.64	0.009
week 31 of the year	–1.15	2.96	–0.39	0.698
week 32 of the year	–8.45	2.36	–3.58	<0.001
week 33 of the year	–4.25	2.36	–1.80	0.074
week 34 of the year	0.56	2.36	0.24	0.812
week 35 of the year	–3.44	2.15	–1.60	0.112
week 36 of the year	–0.28	2.81	–0.10	0.919
week 37 of the year	–8.43	2.16	–3.90	<0.001
week 38 of the year	–0.99	2.15	–0.46	0.646
week 39 of the year	–2.84	2.15	–1.32	0.189
week 40 of the year	0.06	2.15	0.03	0.979
week 41 of the year	–3.27	2.15	–1.52	0.131
week 42 of the year	–1.44	2.16	–0.67	0.505
week 43 of the year	–0.86	2.15	–0.40	0.690
week 44 of the year	–3.18	2.15	–1.48	0.141
week 45 of the year	–8.51	2.80	–3.04	0.003
week 46 of the year	0.32	2.16	0.15	0.884
week 47 of the year	–2.94	2.15	–1.37	0.174
week 48 of the year	–2.67	2.15	–1.24	0.217
week 49 of the year	–2.09	2.15	–0.97	0.333
week 50 of the year	–3.56	2.15	–1.66	0.100
week 51 of the year	–1.27	2.15	–0.59	0.555
week 52 of the year	0.88	2.15	0.41	0.683
holiday in week	–0.54	1.79	–0.30	0.765

Theft of vehicle in Nashville, TN	estimate	SE	statistic	p value
AR(1)	0.98	0.02	59.02	<0.001
MA(1)	–0.51	0.06	–8.00	<0.001
linear trend	0.59	0.24	2.50	0.013
week 2 of the year	–4.36	9.56	–0.46	0.649
week 3 of the year	–9.63	10.39	–0.93	0.355
week 4 of the year	–16.39	11.66	–1.41	0.162
week 5 of the year	–0.19	11.86	–0.02	0.987
week 6 of the year	–5.25	12.42	–0.42	0.673
week 7 of the year	–18.80	12.97	–1.45	0.149
week 8 of the year	3.90	13.49	0.29	0.773
week 9 of the year	–4.63	14.69	–0.31	0.753
week 10 of the year	–30.12	14.79	–2.04	0.043
week 11 of the year	–33.16	14.52	–2.28	0.024
week 12 of the year	–27.36	15.11	–1.81	0.072
week 13 of the year	–29.08	15.60	–1.86	0.064
week 14 of the year	–30.88	15.89	–1.94	0.054
week 15 of the year	–24.93	16.19	–1.54	0.126
week 16 of the year	–46.48	16.51	–2.81	0.006
week 17 of the year	–40.04	17.09	–2.34	0.020
week 18 of the year	–33.40	16.94	–1.97	0.050
week 19 of the year	–44.26	17.13	–2.58	0.011
week 20 of the year	–42.36	17.30	–2.45	0.015
week 21 of the year	–41.47	17.49	–2.37	0.019

Theft of vehicle in Nashville, TN	estimate	SE	statistic	*p* value
week 22 of the year	–40.10	17.62	–2.28	0.024
week 23 of the year	–45.75	17.67	–2.59	0.011
week 24 of the year	–44.65	17.79	–2.51	0.013
week 25 of the year	–38.55	17.84	–2.16	0.032
week 26 of the year	–42.48	17.82	–2.38	0.018
week 27 of the year	–33.42	17.83	–1.87	0.063
week 28 of the year	–33.36	17.82	–1.87	0.063
week 29 of the year	–35.31	17.78	–1.99	0.049
week 30 of the year	–14.02	17.72	–0.79	0.430
week 31 of the year	–20.20	18.27	–1.11	0.270
week 32 of the year	–13.22	17.54	–0.75	0.452
week 33 of the year	–9.96	17.42	–0.57	0.568
week 34 of the year	–3.70	17.27	–0.21	0.830
week 35 of the year	0.53	17.10	0.03	0.975
week 36 of the year	–13.19	17.56	–0.75	0.454
week 37 of the year	–10.51	16.70	–0.63	0.530
week 38 of the year	–7.79	16.46	–0.47	0.637
week 39 of the year	–21.84	16.19	–1.35	0.179
week 40 of the year	–7.64	15.90	–0.48	0.631
week 41 of the year	–20.46	15.59	–1.31	0.191
week 42 of the year	–33.77	15.24	–2.22	0.028
week 43 of the year	–15.35	14.87	–1.03	0.304
week 44 of the year	–1.18	14.46	–0.08	0.935
week 45 of the year	–19.49	14.78	–1.32	0.189
week 46 of the year	–16.39	13.54	–1.21	0.228
week 47 of the year	–27.26	13.02	–2.09	0.038
week 48 of the year	–9.38	12.45	–0.75	0.453
week 49 of the year	0.25	11.83	0.02	0.983
week 50 of the year	–11.14	11.15	–1.00	0.320
week 51 of the year	7.22	10.40	0.69	0.489
week 52 of the year	–11.43	9.57	–1.19	0.234
holiday in week	3.96	4.62	0.86	0.393

Theft of vehicle in Philadelphia, PA	estimate	SE	statistic	*p* value
intercept	39.78	2.60	15.32	<0.001
AR(1)	0.23	0.00	127.63	<0.001
SAR(1)	–0.38	0.00	–167.83	<0.001
linear trend	0.05	0.01	6.40	<0.001
week 2 of the year	0.72	2.99	0.24	0.809
week 3 of the year	1.15	3.30	0.35	0.728
week 4 of the year	2.20	3.70	0.60	0.552
week 5 of the year	3.11	3.42	0.91	0.365
week 6 of the year	3.27	3.40	0.96	0.338
week 7 of the year	–8.13	3.41	–2.39	0.018
week 8 of the year	–6.25	3.40	–1.84	0.068
week 9 of the year	–8.51	3.84	–2.22	0.028
week 10 of the year	–0.24	3.58	–0.07	0.946
week 11 of the year	–0.09	3.21	–0.03	0.978
week 12 of the year	0.95	3.40	0.28	0.780
week 13 of the year	2.09	3.41	0.61	0.542
week 14 of the year	–5.72	3.42	–1.67	0.097

Theft of vehicle in Philadelphia, PA	estimate	SE	statistic	p value
week 15 of the year	–9.09	3.41	–2.66	0.009
week 16 of the year	–7.73	3.43	–2.25	0.026
week 17 of the year	–2.96	3.73	–0.79	0.429
week 18 of the year	–15.15	3.41	–4.44	<0.001
week 19 of the year	–7.89	3.42	–2.31	0.022
week 20 of the year	–11.89	3.41	–3.49	<0.001
week 21 of the year	–4.34	3.43	–1.26	0.208
week 22 of the year	–6.76	3.46	–1.95	0.053
week 23 of the year	–9.68	3.41	–2.84	0.005
week 24 of the year	–8.46	3.45	–2.45	0.015
week 25 of the year	–4.37	3.44	–1.27	0.205
week 26 of the year	–4.13	3.43	–1.20	0.230
week 27 of the year	–2.11	3.41	–0.62	0.538
week 28 of the year	0.39	3.39	0.12	0.908
week 29 of the year	–0.84	3.40	–0.25	0.806
week 30 of the year	1.09	3.40	0.32	0.749
week 31 of the year	–0.39	3.89	–0.10	0.921
week 32 of the year	–0.83	3.40	–0.24	0.808
week 33 of the year	–4.93	3.40	–1.45	0.149
week 34 of the year	2.91	3.40	0.86	0.393
week 35 of the year	–0.06	3.40	–0.02	0.986
week 36 of the year	2.16	3.88	0.56	0.579
week 37 of the year	4.52	3.40	1.33	0.186
week 38 of the year	4.82	3.40	1.42	0.159
week 39 of the year	5.21	3.40	1.53	0.128
week 40 of the year	4.55	3.41	1.33	0.185
week 41 of the year	3.99	3.41	1.17	0.243
week 42 of the year	–1.46	3.40	–0.43	0.668
week 43 of the year	3.85	3.40	1.13	0.259
week 44 of the year	1.26	3.40	0.37	0.711
week 45 of the year	1.62	3.89	0.42	0.677
week 46 of the year	–1.15	3.40	–0.34	0.736
week 47 of the year	–1.21	3.40	–0.36	0.722
week 48 of the year	–1.85	3.40	–0.54	0.587
week 49 of the year	6.52	3.39	1.92	0.057
week 50 of the year	6.33	3.38	1.87	0.063
week 51 of the year	5.80	3.30	1.75	0.081
week 52 of the year	–3.25	2.98	–1.09	0.276
holiday in week	–0.99	1.88	–0.53	0.598

Theft of vehicle in Phoenix, AZ	estimate	SE	statistic	p value
intercept	148.31	4.54	32.70	<0.001
SAR(1)	–0.41	0.07	–5.64	<0.001
linear trend	–0.04	0.01	–3.15	0.002
week 2 of the year	–31.32	6.08	–5.15	<0.001
week 3 of the year	–13.77	6.08	–2.26	0.025
week 4 of the year	–13.84	6.79	–2.04	0.043
week 5 of the year	–3.71	6.13	–0.60	0.546
week 6 of the year	–10.61	6.09	–1.74	0.084
week 7 of the year	–5.34	6.08	–0.88	0.381
week 8 of the year	–12.18	6.08	–2.00	0.047

Theft of vehicle in Phoenix, AZ	estimate	SE	statistic	p value
week 9 of the year	–20.92	7.18	–2.91	0.004
week 10 of the year	–10.15	6.56	–1.55	0.124
week 11 of the year	–16.11	5.73	–2.81	0.006
week 12 of the year	–4.64	6.19	–0.75	0.454
week 13 of the year	–11.58	6.16	–1.88	0.062
week 14 of the year	–21.06	6.11	–3.45	<0.001
week 15 of the year	–27.82	6.11	–4.55	<0.001
week 16 of the year	–20.69	6.15	–3.37	<0.001
week 17 of the year	–28.27	6.80	–4.16	<0.001
week 18 of the year	–28.08	6.09	–4.61	<0.001
week 19 of the year	–31.99	6.11	–5.23	<0.001
week 20 of the year	–36.22	6.09	–5.95	<0.001
week 21 of the year	–14.03	6.14	–2.29	0.024
week 22 of the year	–16.22	6.19	–2.62	0.010
week 23 of the year	–23.51	6.09	–3.86	<0.001
week 24 of the year	–12.79	6.19	–2.07	0.040
week 25 of the year	–13.84	6.14	–2.25	0.026
week 26 of the year	–19.40	6.09	–3.19	0.002
week 27 of the year	–5.67	6.08	–0.93	0.353
week 28 of the year	–19.19	6.09	–3.15	0.002
week 29 of the year	–16.70	6.09	–2.74	0.007
week 30 of the year	–21.56	6.08	–3.54	<0.001
week 31 of the year	–4.56	7.18	–0.64	0.526
week 32 of the year	–9.77	6.08	–1.61	0.110
week 33 of the year	–8.98	6.08	–1.48	0.142
week 34 of the year	–4.12	6.08	–0.68	0.499
week 35 of the year	–15.10	6.08	–2.48	0.014
week 36 of the year	–13.20	7.18	–1.84	0.068
week 37 of the year	–3.02	6.09	–0.50	0.620
week 38 of the year	–3.95	6.08	–0.65	0.517
week 39 of the year	–14.03	6.08	–2.31	0.022
week 40 of the year	–1.95	6.08	–0.32	0.749
week 41 of the year	–7.82	6.09	–1.28	0.201
week 42 of the year	–15.64	6.08	–2.57	0.011
week 43 of the year	–0.39	6.08	–0.06	0.949
week 44 of the year	2.14	6.08	0.35	0.726
week 45 of the year	–5.70	7.18	–0.79	0.429
week 46 of the year	–6.84	6.08	–1.12	0.262
week 47 of the year	–16.70	6.08	–2.75	0.007
week 48 of the year	–4.22	6.08	–0.69	0.489
week 49 of the year	–9.92	6.08	–1.63	0.105
week 50 of the year	–4.75	6.08	–0.78	0.436
week 51 of the year	3.26	6.09	0.53	0.594
week 52 of the year	–15.10	6.08	–2.48	0.014
holiday in week	3.92	3.82	1.03	0.307

Theft of vehicle in Sacramento, CA	estimate	SE	statistic	p value
intercept	79.54	4.24	18.74	<0.001
AR(1)	0.71	0.10	7.33	<0.001
MA(1)	–0.36	0.12	–3.00	0.003
SAR(1)	–0.37	0.08	–4.59	<0.001

Theft of vehicle in Sacramento, CA	estimate	SE	statistic	p value
linear trend	–0.03	0.02	–1.40	0.164
week 2 of the year	–15.07	3.90	–3.87	<0.001
week 3 of the year	–9.30	4.29	–2.17	0.032
week 4 of the year	–6.71	4.94	–1.36	0.176
week 5 of the year	6.05	4.77	1.27	0.207
week 6 of the year	–1.63	4.83	–0.34	0.737
week 7 of the year	–0.69	4.90	–0.14	0.889
week 8 of the year	1.59	4.94	0.32	0.748
week 9 of the year	–13.51	5.51	–2.45	0.015
week 10 of the year	–4.92	5.20	–0.95	0.345
week 11 of the year	–0.37	4.78	–0.08	0.938
week 12 of the year	1.06	5.02	0.21	0.833
week 13 of the year	–8.23	5.07	–1.62	0.107
week 14 of the year	–4.10	5.11	–0.80	0.423
week 15 of the year	–1.30	5.13	–0.25	0.800
week 16 of the year	–6.13	5.18	–1.18	0.238
week 17 of the year	–11.70	5.50	–2.13	0.035
week 18 of the year	–5.88	5.15	–1.14	0.255
week 19 of the year	–9.08	5.15	–1.76	0.080
week 20 of the year	–8.80	5.13	–1.71	0.089
week 21 of the year	–11.78	5.16	–2.28	0.024
week 22 of the year	–10.26	5.18	–1.98	0.049
week 23 of the year	–4.75	5.13	–0.93	0.356
week 24 of the year	–8.72	5.18	–1.68	0.094
week 25 of the year	–4.90	5.16	–0.95	0.343
week 26 of the year	–9.21	5.12	–1.80	0.074
week 27 of the year	–10.15	5.12	–1.98	0.049
week 28 of the year	–17.20	5.12	–3.36	<0.001
week 29 of the year	–15.09	5.12	–2.95	0.004
week 30 of the year	–2.12	5.12	–0.41	0.679
week 31 of the year	–7.33	5.69	–1.29	0.199
week 32 of the year	–12.88	5.11	–2.52	0.013
week 33 of the year	–14.98	5.11	–2.93	0.004
week 34 of the year	–12.54	5.11	–2.46	0.015
week 35 of the year	–5.99	5.10	–1.17	0.243
week 36 of the year	–9.33	5.68	–1.64	0.102
week 37 of the year	–11.59	5.10	–2.27	0.024
week 38 of the year	–23.54	5.10	–4.61	<0.001
week 39 of the year	–4.67	5.09	–0.92	0.360
week 40 of the year	–9.70	5.09	–1.91	0.059
week 41 of the year	–3.51	5.08	–0.69	0.490
week 42 of the year	–7.70	5.07	–1.52	0.131
week 43 of the year	–12.12	5.05	–2.40	0.018
week 44 of the year	–7.67	5.03	–1.52	0.130
week 45 of the year	–19.75	5.58	–3.54	<0.001
week 46 of the year	–5.91	4.97	–1.19	0.236
week 47 of the year	–0.78	4.91	–0.16	0.874
week 48 of the year	–8.04	4.83	–1.67	0.098
week 49 of the year	–2.62	4.71	–0.56	0.579
week 50 of the year	–15.69	4.54	–3.46	<0.001
week 51 of the year	–12.69	4.29	–2.96	0.004
week 52 of the year	–10.69	3.90	–2.74	0.007

Theft of vehicle in Sacramento, CA	estimate	SE	statistic	p value
holiday in week	1.50	2.47	0.61	0.544

Theft of vehicle in San Francisco, CA	estimate	SE	statistic	p value
intercept	125.09	6.68	18.73	<0.001
AR(1)	0.89	0.05	16.22	<0.001
MA(1)	–0.57	0.10	–5.66	<0.001
SAR(1)	–0.39	0.08	–5.15	<0.001
linear trend	–0.19	0.04	–4.91	<0.001
week 2 of the year	–0.53	5.00	–0.11	0.916
week 3 of the year	4.90	5.28	0.93	0.355
week 4 of the year	–7.56	6.05	–1.25	0.213
week 5 of the year	0.05	5.74	0.01	0.993
week 6 of the year	4.08	5.87	0.70	0.488
week 7 of the year	5.01	6.01	0.83	0.405
week 8 of the year	–2.08	6.13	–0.34	0.734
week 9 of the year	–2.73	6.97	–0.39	0.696
week 10 of the year	–11.51	6.68	–1.72	0.087
week 11 of the year	–8.84	6.17	–1.43	0.154
week 12 of the year	–8.76	6.54	–1.34	0.182
week 13 of the year	–0.30	6.62	–0.04	0.964
week 14 of the year	4.01	6.67	0.60	0.548
week 15 of the year	–4.92	6.73	–0.73	0.466
week 16 of the year	–3.40	6.80	–0.50	0.618
week 17 of the year	–2.60	7.25	–0.36	0.720
week 18 of the year	7.48	6.85	1.09	0.277
week 19 of the year	–7.80	6.89	–1.13	0.259
week 20 of the year	4.49	6.91	0.65	0.516
week 21 of the year	–18.12	6.97	–2.60	0.010
week 22 of the year	–9.84	7.01	–1.40	0.162
week 23 of the year	5.90	6.96	0.85	0.397
week 24 of the year	3.02	7.02	0.43	0.668
week 25 of the year	–8.37	7.00	–1.20	0.233
week 26 of the year	0.03	6.97	0.00	0.996
week 27 of the year	–11.60	6.96	–1.67	0.098
week 28 of the year	0.30	6.96	0.04	0.966
week 29 of the year	–0.20	6.96	–0.03	0.978
week 30 of the year	–5.15	6.95	–0.74	0.460
week 31 of the year	–17.85	7.63	–2.34	0.021
week 32 of the year	–9.27	6.92	–1.34	0.182
week 33 of the year	1.66	6.90	0.24	0.811
week 34 of the year	8.14	6.89	1.18	0.239
week 35 of the year	5.19	6.86	0.76	0.451
week 36 of the year	–4.83	7.54	–0.64	0.523
week 37 of the year	–4.26	6.79	–0.63	0.531
week 38 of the year	7.00	6.76	1.04	0.302
week 39 of the year	8.55	6.70	1.28	0.204
week 40 of the year	1.58	6.65	0.24	0.813
week 41 of the year	1.59	6.60	0.24	0.810
week 42 of the year	–5.04	6.52	–0.77	0.441
week 43 of the year	–1.68	6.45	–0.26	0.794
week 44 of the year	–3.71	6.38	–0.58	0.561

Theft of vehicle in San Francisco, CA	estimate	SE	statistic	*p* value
week 45 of the year	–9.77	7.04	–1.39	0.167
week 46 of the year	–6.73	6.16	–1.09	0.277
week 47 of the year	13.21	6.03	2.19	0.030
week 48 of the year	3.80	5.89	0.65	0.519
week 49 of the year	23.09	5.71	4.04	<0.001
week 50 of the year	15.06	5.56	2.71	0.008
week 51 of the year	13.75	5.28	2.61	0.010
week 52 of the year	8.48	5.02	1.69	0.093
holiday in week	3.61	3.18	1.13	0.259

Theft of vehicle in Tucson, AZ	estimate	SE	statistic	*p* value
intercept	29.47	4.35	6.78	<0.001
AR(1)	0.91	0.04	21.30	<0.001
MA(1)	–0.59	0.08	–7.08	<0.001
SAR(1)	–0.33	0.07	–4.37	<0.001
linear trend	0.05	0.03	2.09	0.038
week 2 of the year	6.73	3.00	2.24	0.026
week 3 of the year	2.60	3.16	0.82	0.411
week 4 of the year	1.17	3.58	0.33	0.745
week 5 of the year	11.89	3.42	3.47	<0.001
week 6 of the year	3.98	3.50	1.14	0.258
week 7 of the year	10.37	3.59	2.89	0.004
week 8 of the year	13.07	3.66	3.57	<0.001
week 9 of the year	2.75	4.15	0.66	0.508
week 10 of the year	3.24	4.00	0.81	0.418
week 11 of the year	1.60	3.71	0.43	0.667
week 12 of the year	6.30	3.93	1.60	0.111
week 13 of the year	4.52	3.99	1.13	0.259
week 14 of the year	10.39	4.02	2.58	0.011
week 15 of the year	6.58	4.06	1.62	0.107
week 16 of the year	7.39	4.11	1.80	0.074
week 17 of the year	2.50	4.37	0.57	0.568
week 18 of the year	6.58	4.15	1.59	0.115
week 19 of the year	1.55	4.17	0.37	0.711
week 20 of the year	5.44	4.19	1.30	0.197
week 21 of the year	8.24	4.22	1.95	0.053
week 22 of the year	9.49	4.26	2.23	0.027
week 23 of the year	4.47	4.22	1.06	0.292
week 24 of the year	6.91	4.27	1.62	0.108
week 25 of the year	9.14	4.26	2.14	0.034
week 26 of the year	13.76	4.24	3.24	0.001
week 27 of the year	4.43	4.23	1.05	0.297
week 28 of the year	8.39	4.23	1.98	0.049
week 29 of the year	11.48	4.22	2.72	0.007
week 30 of the year	10.13	4.22	2.40	0.017
week 31 of the year	3.60	4.61	0.78	0.436
week 32 of the year	10.30	4.20	2.45	0.015
week 33 of the year	9.47	4.18	2.27	0.025
week 34 of the year	9.49	4.16	2.28	0.024
week 35 of the year	9.63	4.15	2.32	0.021
week 36 of the year	3.15	4.54	0.69	0.488

Theft of vehicle in Tucson, AZ	estimate	SE	statistic	p value
week 37 of the year	6.36	4.10	1.55	0.123
week 38 of the year	12.66	4.07	3.11	0.002
week 39 of the year	7.80	4.04	1.93	0.055
week 40 of the year	8.38	4.00	2.10	0.038
week 41 of the year	9.18	3.96	2.32	0.022
week 42 of the year	6.60	3.92	1.69	0.094
week 43 of the year	12.59	3.86	3.26	0.001
week 44 of the year	10.73	3.81	2.82	0.005
week 45 of the year	9.52	4.18	2.27	0.024
week 46 of the year	13.78	3.67	3.75	<0.001
week 47 of the year	17.89	3.60	4.98	<0.001
week 48 of the year	17.31	3.50	4.94	<0.001
week 49 of the year	10.63	3.41	3.12	0.002
week 50 of the year	10.40	3.29	3.16	0.002
week 51 of the year	8.72	3.15	2.77	0.006
week 52 of the year	9.75	3.01	3.24	0.001
holiday in week	1.77	1.84	0.96	0.339

Theft of vehicle in Washington, DC	estimate	SE	statistic	p value
intercept	50.20	2.44	20.62	<0.001
AR(1)	0.36	0.33	1.07	0.284
MA(1)	–0.34	0.33	–1.04	0.300
MA(2)	0.18	0.08	2.19	0.030
SAR(1)	–0.55	0.07	–7.93	<0.001
linear trend	–0.05	0.01	–6.22	<0.001
week 2 of the year	–1.13	3.10	–0.36	0.717
week 3 of the year	2.01	2.84	0.71	0.481
week 4 of the year	–4.12	3.46	–1.19	0.236
week 5 of the year	–6.29	3.14	–2.00	0.047
week 6 of the year	–2.74	3.14	–0.87	0.385
week 7 of the year	4.94	3.15	1.57	0.118
week 8 of the year	–2.95	3.15	–0.94	0.350
week 9 of the year	–5.98	3.75	–1.60	0.112
week 10 of the year	–8.58	3.43	–2.50	0.014
week 11 of the year	–8.71	2.96	–2.94	0.004
week 12 of the year	–5.58	3.22	–1.73	0.085
week 13 of the year	–11.14	3.18	–3.50	<0.001
week 14 of the year	–11.12	3.16	–3.52	<0.001
week 15 of the year	–4.69	3.16	–1.48	0.140
week 16 of the year	–11.24	3.19	–3.52	<0.001
week 17 of the year	–6.87	3.56	–1.93	0.056
week 18 of the year	–11.90	3.17	–3.76	<0.001
week 19 of the year	–5.26	3.17	–1.66	0.099
week 20 of the year	–6.93	3.16	–2.19	0.030
week 21 of the year	–9.14	3.19	–2.87	0.005
week 22 of the year	–3.83	3.22	–1.19	0.236
week 23 of the year	–3.11	3.16	–0.98	0.327
week 24 of the year	–3.16	3.22	–0.98	0.328
week 25 of the year	–3.88	3.18	–1.22	0.225
week 26 of the year	0.64	3.16	0.20	0.841
week 27 of the year	4.24	3.16	1.34	0.182

Theft of vehicle in Washington, DC	estimate	SE	statistic	p value
week 28 of the year	2.12	3.16	0.67	0.503
week 29 of the year	1.89	3.16	0.60	0.549
week 30 of the year	7.49	3.16	2.37	0.019
week 31 of the year	1.86	3.76	0.49	0.621
week 32 of the year	3.78	3.16	1.20	0.233
week 33 of the year	8.28	3.16	2.62	0.010
week 34 of the year	13.24	3.16	4.20	<0.001
week 35 of the year	9.53	3.16	3.02	0.003
week 36 of the year	5.47	3.76	1.45	0.148
week 37 of the year	9.49	3.15	3.01	0.003
week 38 of the year	11.79	3.15	3.74	<0.001
week 39 of the year	15.03	3.16	4.76	<0.001
week 40 of the year	7.79	3.15	2.47	0.015
week 41 of the year	9.33	3.16	2.96	0.004
week 42 of the year	13.15	3.15	4.17	<0.001
week 43 of the year	18.79	3.17	5.92	<0.001
week 44 of the year	0.60	3.15	0.19	0.850
week 45 of the year	0.45	3.75	0.12	0.905
week 46 of the year	11.54	3.15	3.66	<0.001
week 47 of the year	8.44	3.15	2.68	0.008
week 48 of the year	5.56	3.14	1.77	0.079
week 49 of the year	0.98	3.11	0.31	0.754
week 50 of the year	6.65	3.04	2.19	0.030
week 51 of the year	6.93	2.85	2.43	0.016
week 52 of the year	0.93	3.10	0.30	0.764
holiday in week	1.78	2.05	0.87	0.385

Theft from vehicle

Theft from vehicle in Atlanta, GA	estimate	SE	statistic	*p* value
intercept	215.16	9.74	22.09	<0.001
AR(1)	0.30	0.07	4.49	<0.001
AR(2)	0.27	0.07	3.90	<0.001
SAR(1)	–0.41	0.08	–5.35	<0.001
linear trend	–0.05	0.04	–1.16	0.250
week 2 of the year	1.17	8.80	0.13	0.895
week 3 of the year	–1.77	8.94	–0.20	0.843
week 4 of the year	4.51	11.02	0.41	0.683
week 5 of the year	19.54	10.48	1.86	0.064
week 6 of the year	21.40	10.75	1.99	0.048
week 7 of the year	38.78	10.95	3.54	<0.001
week 8 of the year	–7.84	11.03	–0.71	0.479
week 9 of the year	–9.08	12.40	–0.73	0.465
week 10 of the year	–55.26	11.46	–4.82	<0.001
week 11 of the year	–42.24	11.38	–3.71	<0.001
week 12 of the year	–37.20	12.26	–3.03	0.003
week 13 of the year	–20.62	11.56	–1.78	0.076
week 14 of the year	–57.32	11.55	–4.96	<0.001
week 15 of the year	–51.94	11.57	–4.49	<0.001
week 16 of the year	–24.00	11.65	–2.06	0.041
week 17 of the year	–51.20	12.39	–4.13	<0.001
week 18 of the year	–78.15	11.55	–6.76	<0.001
week 19 of the year	–75.37	11.54	–6.53	<0.001
week 20 of the year	–68.06	11.54	–5.90	<0.001
week 21 of the year	–76.28	11.59	–6.58	<0.001
week 22 of the year	–58.06	11.65	–4.98	<0.001
week 23 of the year	–31.39	11.53	–2.72	0.007
week 24 of the year	–51.69	11.66	–4.43	<0.001
week 25 of the year	–40.44	11.58	–3.49	<0.001
week 26 of the year	–28.08	11.52	–2.44	0.016
week 27 of the year	–27.69	11.51	–2.40	0.017
week 28 of the year	–24.11	11.50	–2.10	0.038
week 29 of the year	–6.38	11.49	–0.56	0.579
week 30 of the year	–13.23	11.50	–1.15	0.252
week 31 of the year	–15.76	12.84	–1.23	0.222
week 32 of the year	–17.74	11.50	–1.54	0.125
week 33 of the year	–20.72	11.47	–1.81	0.073
week 34 of the year	–29.46	11.49	–2.56	0.011
week 35 of the year	–18.69	11.47	–1.63	0.105
week 36 of the year	–6.06	12.85	–0.47	0.638
week 37 of the year	3.20	11.48	0.28	0.781
week 38 of the year	–13.22	11.44	–1.15	0.250
week 39 of the year	–11.92	11.43	–1.04	0.299
week 40 of the year	–17.38	11.42	–1.52	0.130
week 41 of the year	–15.40	11.40	–1.35	0.179
week 42 of the year	15.49	11.38	1.36	0.175
week 43 of the year	–18.98	11.35	–1.67	0.096
week 44 of the year	–15.34	11.29	–1.36	0.176
week 45 of the year	0.53	12.59	0.04	0.966
week 46 of the year	5.20	11.12	0.47	0.641

Theft from vehicle in Atlanta, GA	estimate	SE	statistic	p value
week 47 of the year	10.75	10.97	0.98	0.328
week 48 of the year	4.81	10.76	0.45	0.655
week 49 of the year	–7.72	10.39	–0.74	0.458
week 50 of the year	4.21	10.04	0.42	0.675
week 51 of the year	3.49	8.91	0.39	0.696
week 52 of the year	8.95	8.78	1.02	0.309
holiday in week	–6.21	5.68	–1.09	0.276

Theft from vehicle in Austin, TX	estimate	SE	statistic	p value
intercept	174.97	13.06	13.40	<0.001
AR(1)	0.93	0.04	20.99	<0.001
MA(1)	–0.56	0.08	–6.68	<0.001
MA(2)	–0.12	0.07	–1.68	0.096
SAR(1)	–0.42	0.08	–5.59	<0.001
linear trend	0.42	0.08	5.29	<0.001
week 2 of the year	–14.07	9.36	–1.50	0.135
week 3 of the year	–15.42	10.41	–1.48	0.141
week 4 of the year	–40.92	11.76	–3.48	<0.001
week 5 of the year	–28.59	10.97	–2.61	0.010
week 6 of the year	–4.69	11.08	–0.42	0.673
week 7 of the year	–11.50	11.25	–1.02	0.309
week 8 of the year	–13.53	11.45	–1.18	0.239
week 9 of the year	–36.49	12.99	–2.81	0.006
week 10 of the year	–31.90	12.44	–2.56	0.011
week 11 of the year	–4.06	11.35	–0.36	0.721
week 12 of the year	–2.92	12.14	–0.24	0.810
week 13 of the year	–16.45	12.23	–1.35	0.181
week 14 of the year	–22.83	12.32	–1.85	0.066
week 15 of the year	–17.40	12.42	–1.40	0.163
week 16 of the year	–24.70	12.59	–1.96	0.052
week 17 of the year	–36.55	13.44	–2.72	0.007
week 18 of the year	–8.02	12.62	–0.64	0.526
week 19 of the year	–18.47	12.61	–1.46	0.145
week 20 of the year	16.29	12.65	1.29	0.200
week 21 of the year	–32.18	12.80	–2.52	0.013
week 22 of the year	–34.88	12.84	–2.72	0.007
week 23 of the year	–20.26	12.75	–1.59	0.114
week 24 of the year	–0.61	12.86	–0.05	0.962
week 25 of the year	–19.65	12.81	–1.53	0.127
week 26 of the year	–37.74	12.78	–2.95	0.004
week 27 of the year	–0.41	12.78	–0.03	0.975
week 28 of the year	–13.85	12.80	–1.08	0.281
week 29 of the year	–14.34	12.79	–1.12	0.264
week 30 of the year	–12.15	12.77	–0.95	0.343
week 31 of the year	–16.91	14.14	–1.20	0.234
week 32 of the year	4.66	12.71	0.37	0.715
week 33 of the year	–22.99	12.62	–1.82	0.070
week 34 of the year	–1.61	12.60	–0.13	0.899
week 35 of the year	–29.62	12.53	–2.36	0.019
week 36 of the year	–37.91	13.93	–2.72	0.007
week 37 of the year	–13.44	12.42	–1.08	0.281

Theft from vehicle in Austin, TX	estimate	SE	statistic	p value
week 38 of the year	6.89	12.35	0.56	0.578
week 39 of the year	–18.94	12.26	–1.54	0.125
week 40 of the year	–15.42	12.18	–1.27	0.208
week 41 of the year	–16.07	12.07	–1.33	0.185
week 42 of the year	–9.23	11.98	–0.77	0.442
week 43 of the year	–40.96	11.88	–3.45	<0.001
week 44 of the year	–28.02	11.80	–2.37	0.019
week 45 of the year	–8.45	13.10	–0.65	0.520
week 46 of the year	–6.51	11.44	–0.57	0.570
week 47 of the year	–19.90	11.31	–1.76	0.081
week 48 of the year	–25.83	11.10	–2.33	0.021
week 49 of the year	–15.54	10.90	–1.43	0.156
week 50 of the year	–23.35	10.67	–2.19	0.030
week 51 of the year	–12.54	10.40	–1.21	0.230
week 52 of the year	1.12	9.30	0.12	0.904
holiday in week	1.52	6.04	0.25	0.802

Theft from vehicle in Baltimore, MD	estimate	SE	statistic	p value
intercept	139.23	5.89	23.65	<0.001
MA(1)	0.32	0.06	5.04	<0.001
SAR(1)	–0.57	0.06	–8.77	<0.001
linear trend	–0.07	0.02	–3.52	<0.001
week 2 of the year	4.85	6.43	0.75	0.451
week 3 of the year	3.42	7.63	0.45	0.655
week 4 of the year	0.47	8.41	0.06	0.956
week 5 of the year	–15.02	7.68	–1.95	0.052
week 6 of the year	–8.43	7.63	–1.10	0.271
week 7 of the year	–23.25	7.64	–3.04	0.003
week 8 of the year	5.46	7.65	0.71	0.477
week 9 of the year	–43.62	8.67	–5.03	<0.001
week 10 of the year	–37.56	8.09	–4.64	<0.001
week 11 of the year	–20.77	7.18	–2.89	0.004
week 12 of the year	–22.51	7.67	–2.93	0.004
week 13 of the year	–25.06	7.66	–3.27	0.001
week 14 of the year	–36.99	7.67	–4.82	<0.001
week 15 of the year	–33.24	7.67	–4.33	<0.001
week 16 of the year	–36.99	7.72	–4.79	<0.001
week 17 of the year	–37.03	8.44	–4.39	<0.001
week 18 of the year	–45.22	7.67	–5.90	<0.001
week 19 of the year	–49.79	7.66	–6.50	<0.001
week 20 of the year	–36.15	7.66	–4.72	<0.001
week 21 of the year	–35.48	7.71	–4.61	<0.001
week 22 of the year	–34.40	7.78	–4.42	<0.001
week 23 of the year	–28.36	7.66	–3.70	<0.001
week 24 of the year	–31.16	7.77	–4.01	<0.001
week 25 of the year	–18.93	7.71	–2.46	0.015
week 26 of the year	–36.67	7.65	–4.79	<0.001
week 27 of the year	–23.94	7.65	–3.13	0.002
week 28 of the year	–14.65	7.65	–1.91	0.057
week 29 of the year	–18.46	7.65	–2.41	0.017
week 30 of the year	–6.48	7.65	–0.85	0.398

Theft from vehicle in Baltimore, MD	estimate	SE	statistic	*p* value
week 31 of the year	–18.85	8.82	–2.14	0.034
week 32 of the year	–16.18	7.64	–2.12	0.036
week 33 of the year	–7.27	7.64	–0.95	0.343
week 34 of the year	–8.59	7.65	–1.12	0.263
week 35 of the year	–1.63	7.64	–0.21	0.832
week 36 of the year	–27.02	8.82	–3.06	0.003
week 37 of the year	–7.38	7.64	–0.97	0.336
week 38 of the year	–12.28	7.64	–1.61	0.110
week 39 of the year	8.99	7.64	1.18	0.241
week 40 of the year	–7.68	7.64	–1.00	0.317
week 41 of the year	10.76	7.64	1.41	0.161
week 42 of the year	0.21	7.64	0.03	0.978
week 43 of the year	22.06	7.64	2.89	0.004
week 44 of the year	–14.57	7.64	–1.91	0.058
week 45 of the year	–12.82	8.83	–1.45	0.148
week 46 of the year	3.29	7.64	0.43	0.667
week 47 of the year	1.28	7.64	0.17	0.867
week 48 of the year	10.07	7.64	1.32	0.189
week 49 of the year	–17.62	7.63	–2.31	0.022
week 50 of the year	9.86	7.64	1.29	0.199
week 51 of the year	41.16	7.64	5.39	<0.001
week 52 of the year	18.11	6.43	2.82	0.005
holiday in week	5.95	4.40	1.35	0.179

Theft from vehicle in Boston, MA	estimate	SE	statistic	*p* value
intercept	61.97	3.06	20.25	<0.001
MA(1)	0.06	0.07	0.82	0.416
SAR(1)	–0.53	0.07	–7.30	<0.001
linear trend	–0.08	0.01	–9.53	<0.001
week 2 of the year	–12.43	4.04	–3.08	0.003
week 3 of the year	4.39	4.17	1.05	0.294
week 4 of the year	9.45	4.98	1.90	0.060
week 5 of the year	–4.39	4.18	–1.05	0.295
week 6 of the year	–7.03	4.18	–1.68	0.095
week 7 of the year	–9.55	4.17	–2.29	0.024
week 8 of the year	–5.32	4.17	–1.27	0.205
week 9 of the year	–10.94	4.97	–2.20	0.029
week 10 of the year	–5.17	4.42	–1.17	0.244
week 11 of the year	–9.30	3.85	–2.42	0.017
week 12 of the year	–9.92	4.45	–2.23	0.027
week 13 of the year	–0.41	3.92	–0.10	0.917
week 14 of the year	–21.89	3.84	–5.70	<0.001
week 15 of the year	–21.19	3.84	–5.51	<0.001
week 16 of the year	–10.49	3.89	–2.70	0.008
week 17 of the year	–5.59	4.43	–1.26	0.209
week 18 of the year	–15.02	3.85	–3.90	<0.001
week 19 of the year	–17.36	3.84	–4.52	<0.001
week 20 of the year	–19.63	3.84	–5.11	<0.001
week 21 of the year	–10.35	3.88	–2.67	0.008
week 22 of the year	–10.49	3.93	–2.67	0.009
week 23 of the year	–17.02	3.85	–4.42	<0.001

Theft from vehicle in Boston, MA	estimate	SE	statistic	p value
week 24 of the year	–8.17	3.92	–2.08	0.039
week 25 of the year	–5.84	3.88	–1.51	0.134
week 26 of the year	–8.08	3.85	–2.10	0.038
week 27 of the year	–7.29	3.85	–1.89	0.060
week 28 of the year	–13.07	3.84	–3.41	<0.001
week 29 of the year	–5.17	3.84	–1.34	0.181
week 30 of the year	–6.97	3.85	–1.81	0.072
week 31 of the year	–3.62	4.71	–0.77	0.444
week 32 of the year	–4.09	3.85	–1.06	0.290
week 33 of the year	0.19	3.85	0.05	0.961
week 34 of the year	4.93	3.84	1.28	0.202
week 35 of the year	2.31	3.84	0.60	0.549
week 36 of the year	1.54	4.71	0.33	0.743
week 37 of the year	5.90	3.85	1.53	0.128
week 38 of the year	9.30	3.84	2.42	0.017
week 39 of the year	17.60	3.84	4.58	<0.001
week 40 of the year	4.86	3.85	1.26	0.209
week 41 of the year	4.96	3.84	1.29	0.199
week 42 of the year	11.90	3.85	3.09	0.002
week 43 of the year	8.45	3.85	2.20	0.030
week 44 of the year	7.83	3.85	2.04	0.044
week 45 of the year	1.39	4.71	0.30	0.768
week 46 of the year	1.50	3.85	0.39	0.697
week 47 of the year	4.46	3.85	1.16	0.248
week 48 of the year	–0.79	3.84	–0.20	0.838
week 49 of the year	5.87	4.18	1.40	0.163
week 50 of the year	4.08	4.16	0.98	0.329
week 51 of the year	–3.10	4.19	–0.74	0.461
week 52 of the year	–1.12	4.04	–0.28	0.781
holiday in week	–4.88	2.73	–1.79	0.076

Theft from vehicle in Los Angeles, CA	estimate	SE	statistic	p value
intercept	637.86	14.01	45.52	<0.001
AR(1)	0.39	0.07	5.66	<0.001
AR(2)	0.14	0.07	1.99	0.048
SAR(1)	–0.43	0.07	–5.76	<0.001
linear trend	–0.05	0.06	–0.78	0.438
week 2 of the year	0.99	12.50	0.08	0.937
week 3 of the year	–0.32	14.01	–0.02	0.982
week 4 of the year	24.21	16.56	1.46	0.146
week 5 of the year	35.89	16.02	2.24	0.027
week 6 of the year	29.70	16.30	1.82	0.070
week 7 of the year	49.76	16.53	3.01	0.003
week 8 of the year	66.57	16.63	4.00	<0.001
week 9 of the year	6.56	18.28	0.36	0.720
week 10 of the year	–11.70	17.32	–0.68	0.500
week 11 of the year	11.69	15.93	0.73	0.464
week 12 of the year	50.37	16.71	3.02	0.003
week 13 of the year	–6.84	16.84	–0.41	0.685
week 14 of the year	26.73	16.95	1.58	0.117
week 15 of the year	–25.67	17.07	–1.50	0.135

Theft from vehicle in Los Angeles, CA	estimate	SE	statistic	p value
week 16 of the year	23.45	17.15	1.37	0.174
week 17 of the year	7.08	18.29	0.39	0.699
week 18 of the year	–4.24	17.06	–0.25	0.804
week 19 of the year	–30.73	17.07	–1.80	0.074
week 20 of the year	–25.80	17.06	–1.51	0.133
week 21 of the year	–3.57	17.13	–0.21	0.835
week 22 of the year	–2.51	17.23	–0.15	0.884
week 23 of the year	–10.11	17.05	–0.59	0.554
week 24 of the year	–5.97	17.19	–0.35	0.729
week 25 of the year	13.67	17.12	0.80	0.426
week 26 of the year	20.78	17.04	1.22	0.225
week 27 of the year	–43.47	17.01	–2.56	0.012
week 28 of the year	–10.90	17.04	–0.64	0.523
week 29 of the year	3.64	17.01	0.21	0.831
week 30 of the year	20.27	16.99	1.19	0.235
week 31 of the year	0.37	18.82	0.02	0.984
week 32 of the year	16.05	16.99	0.94	0.346
week 33 of the year	–33.99	16.98	–2.00	0.047
week 34 of the year	26.63	16.97	1.57	0.119
week 35 of the year	–28.06	16.97	–1.65	0.100
week 36 of the year	–36.41	18.79	–1.94	0.055
week 37 of the year	0.61	16.98	0.04	0.971
week 38 of the year	4.45	16.97	0.26	0.793
week 39 of the year	–5.08	16.98	–0.30	0.765
week 40 of the year	7.91	16.95	0.47	0.642
week 41 of the year	–34.01	16.93	–2.01	0.046
week 42 of the year	–0.24	16.91	–0.01	0.989
week 43 of the year	0.72	16.89	0.04	0.966
week 44 of the year	–28.21	16.86	–1.67	0.096
week 45 of the year	–5.88	18.66	–0.32	0.753
week 46 of the year	3.53	16.71	0.21	0.833
week 47 of the year	–8.15	16.58	–0.49	0.624
week 48 of the year	–5.75	16.33	–0.35	0.725
week 49 of the year	12.03	15.92	0.76	0.451
week 50 of the year	–0.05	15.26	0.00	0.998
week 51 of the year	41.14	14.01	2.94	0.004
week 52 of the year	13.72	12.52	1.10	0.275
holiday in week	–7.69	8.09	–0.95	0.344

Theft from vehicle in Louisville, KY	estimate	SE	statistic	p value
intercept	110.97	6.14	18.07	<0.001
AR(1)	0.76	0.07	10.61	<0.001
AR(2)	–0.10	0.08	–1.21	0.228
AR(3)	0.12	0.08	1.48	0.140
MA(1)	–0.44	0.08	–5.79	<0.001
SAR(1)	–0.47	0.01	–42.91	<0.001
linear trend	–0.02	0.03	–0.82	0.415
week 2 of the year	–17.71	5.52	–3.21	0.002
week 3 of the year	–11.13	6.14	–1.81	0.072
week 4 of the year	3.10	6.70	0.46	0.644
week 5 of the year	–12.40	6.26	–1.98	0.049

Theft from vehicle in Louisville, KY	estimate	SE	statistic	p value
week 6 of the year	–20.35	6.42	–3.17	0.002
week 7 of the year	–17.63	6.53	–2.70	0.008
week 8 of the year	–14.79	6.62	–2.23	0.027
week 9 of the year	–22.99	7.55	–3.04	0.003
week 10 of the year	–13.95	7.16	–1.95	0.053
week 11 of the year	–18.85	6.49	–2.91	0.004
week 12 of the year	–13.14	6.91	–1.90	0.059
week 13 of the year	–20.77	6.95	–2.99	0.003
week 14 of the year	–40.55	6.98	–5.81	<0.001
week 15 of the year	–26.50	7.01	–3.78	<0.001
week 16 of the year	–14.84	7.07	–2.10	0.037
week 17 of the year	–21.06	7.62	–2.76	0.006
week 18 of the year	–28.20	7.08	–3.98	<0.001
week 19 of the year	–34.38	7.09	–4.85	<0.001
week 20 of the year	–22.23	7.10	–3.13	0.002
week 21 of the year	–22.45	7.14	–3.14	0.002
week 22 of the year	–10.83	7.19	–1.51	0.134
week 23 of the year	–14.53	7.11	–2.04	0.043
week 24 of the year	–19.25	7.19	–2.68	0.008
week 25 of the year	–18.55	7.15	–2.59	0.010
week 26 of the year	–15.43	7.11	–2.17	0.032
week 27 of the year	–19.20	7.11	–2.70	0.008
week 28 of the year	–24.76	7.10	–3.49	<0.001
week 29 of the year	–7.63	7.10	–1.07	0.284
week 30 of the year	–6.54	7.10	–0.92	0.358
week 31 of the year	–1.26	7.99	–0.16	0.875
week 32 of the year	4.47	7.08	0.63	0.529
week 33 of the year	–6.24	7.08	–0.88	0.379
week 34 of the year	3.66	7.07	0.52	0.605
week 35 of the year	–9.72	7.06	–1.38	0.170
week 36 of the year	–1.78	7.95	–0.22	0.823
week 37 of the year	4.36	7.03	0.62	0.537
week 38 of the year	6.81	7.01	0.97	0.333
week 39 of the year	10.53	6.99	1.51	0.134
week 40 of the year	–9.85	6.97	–1.41	0.160
week 41 of the year	–5.88	6.94	–0.85	0.398
week 42 of the year	–5.41	6.90	–0.78	0.434
week 43 of the year	–4.79	6.86	–0.70	0.485
week 44 of the year	–1.47	6.80	–0.22	0.830
week 45 of the year	7.44	7.67	0.97	0.334
week 46 of the year	–5.17	6.65	–0.78	0.438
week 47 of the year	8.86	6.55	1.35	0.178
week 48 of the year	–7.80	6.43	–1.21	0.227
week 49 of the year	1.14	6.22	0.18	0.855
week 50 of the year	1.94	6.05	0.32	0.749
week 51 of the year	2.14	6.14	0.35	0.727
week 52 of the year	–7.66	5.52	–1.39	0.167
holiday in week	–10.85	3.65	–2.97	0.003

Theft from vehicle in Memphis, TN	estimate	SE	statistic	p value
intercept	154.40	9.60	16.08	<0.001

Theft from vehicle in Memphis, TN	estimate	SE	statistic	p value
AR(1)	0.90	0.06	14.52	<0.001
MA(1)	–0.64	0.11	–5.64	<0.001
SAR(1)	–0.41	0.08	–5.18	<0.001
linear trend	0.06	0.05	1.15	0.252
week 2 of the year	–13.33	7.80	–1.71	0.089
week 3 of the year	–30.61	8.08	–3.79	<0.001
week 4 of the year	–13.05	9.22	–1.42	0.159
week 5 of the year	1.67	8.60	0.19	0.847
week 6 of the year	–17.19	8.71	–1.97	0.050
week 7 of the year	–22.24	8.87	–2.51	0.013
week 8 of the year	–12.90	9.01	–1.43	0.154
week 9 of the year	–10.84	10.41	–1.04	0.299
week 10 of the year	–17.66	9.90	–1.78	0.076
week 11 of the year	–31.92	8.97	–3.56	<0.001
week 12 of the year	–12.10	9.59	–1.26	0.209
week 13 of the year	–14.28	9.62	–1.48	0.140
week 14 of the year	–38.83	9.66	–4.02	<0.001
week 15 of the year	–17.64	9.73	–1.81	0.072
week 16 of the year	–30.06	9.85	–3.05	0.003
week 17 of the year	–38.98	10.60	–3.68	<0.001
week 18 of the year	–45.75	9.89	–4.63	<0.001
week 19 of the year	–45.36	9.92	–4.57	<0.001
week 20 of the year	–32.60	9.95	–3.28	0.001
week 21 of the year	–31.72	10.04	–3.16	0.002
week 22 of the year	–26.75	10.11	–2.65	0.009
week 23 of the year	–54.44	10.01	–5.44	<0.001
week 24 of the year	–26.09	10.13	–2.58	0.011
week 25 of the year	–5.33	10.08	–0.53	0.598
week 26 of the year	–15.41	10.02	–1.54	0.126
week 27 of the year	–36.42	10.02	–3.63	<0.001
week 28 of the year	–27.51	10.03	–2.74	0.007
week 29 of the year	–4.50	10.00	–0.45	0.653
week 30 of the year	–6.29	9.99	–0.63	0.530
week 31 of the year	–5.15	11.20	–0.46	0.646
week 32 of the year	–21.41	9.96	–2.15	0.033
week 33 of the year	–16.83	9.94	–1.69	0.092
week 34 of the year	–10.90	9.93	–1.10	0.274
week 35 of the year	–10.96	9.87	–1.11	0.268
week 36 of the year	16.98	11.07	1.53	0.127
week 37 of the year	5.43	9.79	0.55	0.580
week 38 of the year	13.85	9.74	1.42	0.157
week 39 of the year	34.51	9.68	3.56	<0.001
week 40 of the year	35.86	9.62	3.73	<0.001
week 41 of the year	11.53	9.54	1.21	0.229
week 42 of the year	2.31	9.49	0.24	0.808
week 43 of the year	4.75	9.39	0.51	0.614
week 44 of the year	19.42	9.29	2.09	0.038
week 45 of the year	45.26	10.47	4.32	<0.001
week 46 of the year	11.44	9.03	1.27	0.207
week 47 of the year	39.17	8.89	4.40	<0.001
week 48 of the year	19.03	8.72	2.18	0.031
week 49 of the year	28.05	8.56	3.28	0.001

Theft from vehicle in Memphis, TN	estimate	SE	statistic	p value
week 50 of the year	16.34	8.32	1.96	0.052
week 51 of the year	–8.18	8.07	–1.01	0.313
week 52 of the year	5.01	7.82	0.64	0.523
holiday in week	–17.61	5.02	–3.51	<0.001

Theft from vehicle in Minneapolis, MN	estimate	SE	statistic	p value
intercept	63.39	4.79	13.23	<0.001
AR(1)	0.23	0.07	3.33	0.001
AR(2)	0.09	0.07	1.32	0.189
AR(3)	0.13	0.07	1.96	0.052
SAR(1)	–0.34	0.08	–4.33	<0.001
linear trend	0.16	0.02	8.22	<0.001
week 2 of the year	1.84	5.03	0.37	0.714
week 3 of the year	–1.89	5.32	–0.35	0.723
week 4 of the year	–2.75	5.80	–0.48	0.635
week 5 of the year	–25.42	5.64	–4.51	<0.001
week 6 of the year	–13.77	5.72	–2.41	0.017
week 7 of the year	–18.65	5.74	–3.25	0.001
week 8 of the year	–18.63	5.81	–3.21	0.002
week 9 of the year	–27.74	6.56	–4.23	<0.001
week 10 of the year	–30.90	6.11	–5.06	<0.001
week 11 of the year	–29.20	5.56	–5.25	<0.001
week 12 of the year	–19.26	5.88	–3.28	0.001
week 13 of the year	–23.78	5.93	–4.01	<0.001
week 14 of the year	–30.25	5.91	–5.12	<0.001
week 15 of the year	–29.89	5.92	–5.05	<0.001
week 16 of the year	–24.60	5.98	–4.11	<0.001
week 17 of the year	–24.11	6.42	–3.76	<0.001
week 18 of the year	–25.93	5.94	–4.37	<0.001
week 19 of the year	–29.72	5.94	–5.00	<0.001
week 20 of the year	–28.82	5.95	–4.85	<0.001
week 21 of the year	–23.21	5.98	–3.88	<0.001
week 22 of the year	–11.18	6.00	–1.86	0.064
week 23 of the year	–27.20	5.94	–4.58	<0.001
week 24 of the year	–26.46	6.01	–4.40	<0.001
week 25 of the year	–19.80	5.98	–3.31	0.001
week 26 of the year	–14.89	5.94	–2.51	0.013
week 27 of the year	–18.98	5.94	–3.20	0.002
week 28 of the year	–9.34	5.93	–1.57	0.118
week 29 of the year	–14.23	5.94	–2.39	0.018
week 30 of the year	–3.14	5.97	–0.53	0.600
week 31 of the year	–12.94	6.68	–1.94	0.055
week 32 of the year	–9.29	5.95	–1.56	0.121
week 33 of the year	2.15	5.93	0.36	0.717
week 34 of the year	–5.77	5.92	–0.97	0.332
week 35 of the year	–0.59	5.93	–0.10	0.920
week 36 of the year	7.10	6.67	1.06	0.289
week 37 of the year	10.64	5.92	1.80	0.074
week 38 of the year	–3.89	5.92	–0.66	0.512
week 39 of the year	7.74	5.91	1.31	0.193
week 40 of the year	–3.58	5.92	–0.60	0.546

Theft from vehicle in Minneapolis, MN	estimate	SE	statistic	*p* value
week 41 of the year	9.63	5.91	1.63	0.105
week 42 of the year	3.21	5.90	0.54	0.587
week 43 of the year	–3.20	5.90	–0.54	0.588
week 44 of the year	11.79	5.88	2.01	0.047
week 45 of the year	–12.64	6.63	–1.91	0.058
week 46 of the year	2.90	5.83	0.50	0.620
week 47 of the year	5.38	5.78	0.93	0.353
week 48 of the year	1.17	5.72	0.20	0.838
week 49 of the year	14.25	5.60	2.55	0.012
week 50 of the year	3.50	5.28	0.66	0.509
week 51 of the year	9.79	5.31	1.84	0.067
week 52 of the year	10.09	5.00	2.02	0.046
holiday in week	0.56	3.07	0.18	0.857

Theft from vehicle in Montgomery County, MD	estimate	SE	statistic	*p* value
intercept	87.43	7.25	12.06	<0.001
AR(1)	0.89	0.07	12.35	<0.001
MA(1)	–0.68	0.12	–5.90	<0.001
SAR(1)	–0.55	0.08	–7.04	<0.001
linear trend	–0.06	0.04	–1.32	0.189
week 2 of the year	12.52	5.49	2.28	0.024
week 3 of the year	10.30	5.64	1.83	0.070
week 4 of the year	5.08	7.07	0.72	0.474
week 5 of the year	0.48	5.96	0.08	0.936
week 6 of the year	–0.94	5.98	–0.16	0.876
week 7 of the year	10.45	6.06	1.72	0.087
week 8 of the year	0.91	6.14	0.15	0.883
week 9 of the year	–3.10	8.04	–0.39	0.700
week 10 of the year	–3.97	8.09	–0.49	0.624
week 11 of the year	12.27	6.33	1.94	0.055
week 12 of the year	–7.11	6.88	–1.03	0.303
week 13 of the year	4.18	7.10	0.59	0.558
week 14 of the year	–10.03	7.06	–1.42	0.158
week 15 of the year	6.46	7.11	0.91	0.366
week 16 of the year	9.60	7.15	1.34	0.181
week 17 of the year	10.40	8.72	1.19	0.235
week 18 of the year	0.96	7.21	0.13	0.894
week 19 of the year	–4.59	7.23	–0.63	0.527
week 20 of the year	–12.42	7.25	–1.71	0.089
week 21 of the year	–12.48	7.26	–1.72	0.088
week 22 of the year	–4.12	7.59	–0.54	0.588
week 23 of the year	–9.17	7.30	–1.26	0.212
week 24 of the year	3.31	7.42	0.45	0.656
week 25 of the year	–0.58	7.43	–0.08	0.938
week 26 of the year	–5.21	7.30	–0.71	0.476
week 27 of the year	–5.94	7.30	–0.81	0.417
week 28 of the year	–7.25	7.29	–0.99	0.322
week 29 of the year	0.58	7.29	0.08	0.936
week 30 of the year	5.45	7.29	0.75	0.456
week 31 of the year	1.18	8.87	0.13	0.895
week 32 of the year	16.64	7.29	2.28	0.024

Theft from vehicle in Montgomery County, MD	estimate	SE	statistic	*p* value
week 33 of the year	–3.08	7.26	–0.42	0.672
week 34 of the year	12.17	7.26	1.68	0.096
week 35 of the year	1.15	6.64	0.17	0.863
week 36 of the year	–7.90	8.29	–0.95	0.343
week 37 of the year	6.13	6.56	0.94	0.351
week 38 of the year	3.25	6.54	0.50	0.620
week 39 of the year	13.84	6.50	2.13	0.035
week 40 of the year	16.18	6.46	2.50	0.013
week 41 of the year	4.45	6.42	0.69	0.490
week 42 of the year	20.14	6.38	3.16	0.002
week 43 of the year	25.50	6.33	4.03	<0.001
week 44 of the year	12.00	6.27	1.91	0.058
week 45 of the year	12.03	8.02	1.50	0.136
week 46 of the year	3.56	6.15	0.58	0.564
week 47 of the year	10.50	6.06	1.73	0.086
week 48 of the year	12.50	5.97	2.09	0.038
week 49 of the year	10.93	5.88	1.86	0.065
week 50 of the year	10.11	5.79	1.75	0.083
week 51 of the year	20.82	5.64	3.69	<0.001
week 52 of the year	8.98	5.49	1.64	0.104
holiday in week	–3.21	5.09	–0.63	0.529

Theft from vehicle in Nashville, TN	estimate	SE	statistic	*p* value
AR(1)	0.99	0.01	67.11	<0.001
MA(1)	–0.52	0.07	–7.25	<0.001
linear trend	1.88	0.83	2.27	0.025
week 2 of the year	–60.72	26.97	–2.25	0.026
week 3 of the year	–97.64	29.26	–3.34	0.001
week 4 of the year	–121.94	32.86	–3.71	<0.001
week 5 of the year	–72.98	33.37	–2.19	0.030
week 6 of the year	–97.99	34.91	–2.81	0.006
week 7 of the year	–92.71	36.49	–2.54	0.012
week 8 of the year	–99.20	37.94	–2.61	0.010
week 9 of the year	–66.27	41.47	–1.60	0.112
week 10 of the year	–133.62	42.01	–3.18	0.002
week 11 of the year	–152.95	41.11	–3.72	<0.001
week 12 of the year	–155.63	42.82	–3.63	<0.001
week 13 of the year	–151.80	43.81	–3.47	<0.001
week 14 of the year	–169.33	44.53	–3.80	<0.001
week 15 of the year	–169.83	45.33	–3.75	<0.001
week 16 of the year	–175.30	46.21	–3.79	<0.001
week 17 of the year	–182.75	47.83	–3.82	<0.001
week 18 of the year	–178.70	47.28	–3.78	<0.001
week 19 of the year	–184.78	47.79	–3.87	<0.001
week 20 of the year	–168.13	48.23	–3.49	<0.001
week 21 of the year	–148.71	48.76	–3.05	0.003
week 22 of the year	–157.10	49.07	–3.20	0.002
week 23 of the year	–149.06	49.17	–3.03	0.003
week 24 of the year	–145.44	49.51	–2.94	0.004
week 25 of the year	–166.14	49.63	–3.35	0.001
week 26 of the year	–110.15	49.56	–2.22	0.028

Theft from vehicle in Nashville, TN	estimate	SE	statistic	p value
week 27 of the year	–149.64	49.57	–3.02	0.003
week 28 of the year	–140.90	49.52	–2.85	0.005
week 29 of the year	–133.92	49.41	–2.71	0.007
week 30 of the year	–111.96	49.24	–2.27	0.024
week 31 of the year	–104.84	50.88	–2.06	0.041
week 32 of the year	–21.60	48.74	–0.44	0.658
week 33 of the year	–74.69	48.40	–1.54	0.125
week 34 of the year	–49.05	47.99	–1.02	0.308
week 35 of the year	–58.43	47.53	–1.23	0.221
week 36 of the year	–65.40	48.92	–1.34	0.183
week 37 of the year	–62.00	46.41	–1.34	0.184
week 38 of the year	–30.18	45.76	–0.66	0.511
week 39 of the year	–28.13	45.03	–0.62	0.533
week 40 of the year	–64.34	44.24	–1.45	0.148
week 41 of the year	–77.06	43.37	–1.78	0.078
week 42 of the year	–48.06	42.43	–1.13	0.259
week 43 of the year	–28.32	41.40	–0.68	0.495
week 44 of the year	–23.60	40.29	–0.59	0.559
week 45 of the year	–62.47	41.30	–1.51	0.132
week 46 of the year	–51.71	37.77	–1.37	0.173
week 47 of the year	–36.03	36.35	–0.99	0.323
week 48 of the year	–87.87	34.81	–2.52	0.013
week 49 of the year	–90.48	33.12	–2.73	0.007
week 50 of the year	–37.11	31.27	–1.19	0.237
week 51 of the year	16.50	29.23	0.56	0.573
week 52 of the year	–52.15	26.95	–1.93	0.055
holiday in week	3.82	13.03	0.29	0.770

Theft from vehicle in Philadelphia, PA	estimate	SE	statistic	p value
intercept	282.56	12.25	23.07	<0.001
AR(1)	0.68	0.09	7.49	<0.001
MA(1)	–0.31	0.11	–2.84	0.005
SAR(1)	–0.24	0.08	–3.07	0.003
linear trend	–0.03	0.05	–0.64	0.521
week 2 of the year	–32.44	11.27	–2.88	0.005
week 3 of the year	–36.85	12.55	–2.94	0.004
week 4 of the year	–47.28	14.29	–3.31	0.001
week 5 of the year	–24.84	13.94	–1.78	0.077
week 6 of the year	–40.99	14.23	–2.88	0.005
week 7 of the year	–48.93	14.43	–3.39	<0.001
week 8 of the year	–43.61	14.55	–3.00	0.003
week 9 of the year	–51.02	15.84	–3.22	0.002
week 10 of the year	–61.06	15.05	–4.06	<0.001
week 11 of the year	–55.25	14.04	–3.94	<0.001
week 12 of the year	–47.82	14.63	–3.27	0.001
week 13 of the year	–37.53	14.87	–2.52	0.013
week 14 of the year	–45.59	14.95	–3.05	0.003
week 15 of the year	–64.40	14.99	–4.30	<0.001
week 16 of the year	–37.90	15.10	–2.51	0.013
week 17 of the year	–43.58	15.89	–2.74	0.007
week 18 of the year	–68.26	15.03	–4.54	<0.001

Theft from vehicle in Philadelphia, PA	estimate	SE	statistic	p value
week 19 of the year	–72.60	15.04	–4.83	<0.001
week 20 of the year	–69.01	15.05	–4.59	<0.001
week 21 of the year	–41.99	15.10	–2.78	0.006
week 22 of the year	–38.04	15.13	–2.51	0.013
week 23 of the year	–30.13	15.02	–2.01	0.047
week 24 of the year	–53.17	15.13	–3.51	<0.001
week 25 of the year	2.10	15.08	0.14	0.890
week 26 of the year	–21.78	15.02	–1.45	0.149
week 27 of the year	–42.13	15.00	–2.81	0.006
week 28 of the year	–15.68	15.02	–1.04	0.298
week 29 of the year	9.15	14.99	0.61	0.542
week 30 of the year	–12.09	14.99	–0.81	0.421
week 31 of the year	2.10	16.37	0.13	0.898
week 32 of the year	–6.80	15.01	–0.45	0.651
week 33 of the year	2.20	14.99	0.15	0.884
week 34 of the year	18.54	14.96	1.24	0.217
week 35 of the year	–7.34	14.97	–0.49	0.625
week 36 of the year	–7.61	16.35	–0.47	0.642
week 37 of the year	7.60	14.95	0.51	0.612
week 38 of the year	28.67	14.94	1.92	0.057
week 39 of the year	24.79	14.93	1.66	0.099
week 40 of the year	11.43	14.92	0.77	0.445
week 41 of the year	5.98	14.91	0.40	0.689
week 42 of the year	25.95	14.88	1.74	0.083
week 43 of the year	21.43	14.85	1.44	0.151
week 44 of the year	1.13	14.80	0.08	0.939
week 45 of the year	13.50	16.13	0.84	0.404
week 46 of the year	13.41	14.62	0.92	0.360
week 47 of the year	16.82	14.45	1.16	0.246
week 48 of the year	8.41	14.22	0.59	0.555
week 49 of the year	–23.12	13.88	–1.67	0.098
week 50 of the year	–7.36	13.35	–0.55	0.582
week 51 of the year	7.77	12.55	0.62	0.536
week 52 of the year	–6.85	11.27	–0.61	0.544
holiday in week	–5.52	6.62	–0.83	0.406

Theft from vehicle in Sacramento, CA	estimate	SE	statistic	p value
intercept	47.26	6.13	7.70	<0.001
AR(1)	0.43	0.07	6.35	<0.001
AR(2)	–0.02	0.08	–0.25	0.803
AR(3)	0.25	0.07	3.54	<0.001
SAR(1)	–0.33	0.09	–3.65	<0.001
linear trend	0.15	0.03	4.73	<0.001
week 2 of the year	–10.11	4.91	–2.06	0.041
week 3 of the year	–1.52	5.72	–0.27	0.791
week 4 of the year	–2.30	5.90	–0.39	0.697
week 5 of the year	–0.45	5.91	–0.08	0.940
week 6 of the year	11.63	6.22	1.87	0.064
week 7 of the year	9.68	6.28	1.54	0.125
week 8 of the year	3.45	6.41	0.54	0.591
week 9 of the year	–0.52	7.05	–0.07	0.941

Theft from vehicle in Sacramento, CA	estimate	SE	statistic	p value
week 10 of the year	–3.10	6.74	–0.46	0.647
week 11 of the year	6.95	6.37	1.09	0.277
week 12 of the year	13.92	6.64	2.10	0.038
week 13 of the year	12.14	6.78	1.79	0.075
week 14 of the year	7.15	6.89	1.04	0.301
week 15 of the year	4.64	6.95	0.67	0.506
week 16 of the year	1.04	6.96	0.15	0.882
week 17 of the year	9.08	7.30	1.24	0.215
week 18 of the year	0.57	6.99	0.08	0.935
week 19 of the year	1.17	6.99	0.17	0.867
week 20 of the year	–7.47	6.99	–1.07	0.287
week 21 of the year	1.18	7.02	0.17	0.866
week 22 of the year	–2.37	7.03	–0.34	0.736
week 23 of the year	–2.58	6.99	–0.37	0.713
week 24 of the year	–5.08	7.04	–0.72	0.472
week 25 of the year	4.66	7.00	0.66	0.507
week 26 of the year	1.70	6.98	0.24	0.807
week 27 of the year	–4.33	6.97	–0.62	0.535
week 28 of the year	–6.63	6.97	–0.95	0.343
week 29 of the year	–4.58	6.97	–0.66	0.512
week 30 of the year	–3.20	6.96	–0.46	0.647
week 31 of the year	–8.18	7.51	–1.09	0.278
week 32 of the year	–17.98	6.94	–2.59	0.011
week 33 of the year	–18.76	6.93	–2.71	0.008
week 34 of the year	–4.06	6.93	–0.59	0.559
week 35 of the year	–8.49	6.92	–1.23	0.222
week 36 of the year	–9.75	7.48	–1.30	0.195
week 37 of the year	–3.60	6.89	–0.52	0.602
week 38 of the year	–10.39	6.87	–1.51	0.132
week 39 of the year	1.17	6.85	0.17	0.865
week 40 of the year	–13.14	6.83	–1.92	0.056
week 41 of the year	–6.80	6.80	–1.00	0.319
week 42 of the year	–13.02	6.75	–1.93	0.056
week 43 of the year	–10.96	6.69	–1.64	0.104
week 44 of the year	–9.08	6.64	–1.37	0.174
week 45 of the year	–1.03	7.17	–0.14	0.886
week 46 of the year	–2.53	6.43	–0.39	0.695
week 47 of the year	–2.38	6.31	–0.38	0.706
week 48 of the year	3.40	6.23	0.55	0.586
week 49 of the year	2.78	5.88	0.47	0.638
week 50 of the year	–4.34	5.47	–0.79	0.429
week 51 of the year	0.58	5.72	0.10	0.920
week 52 of the year	–3.74	4.91	–0.76	0.448
holiday in week	–0.27	2.85	–0.10	0.924

Theft from vehicle in San Francisco, CA	estimate	SE	statistic	p value
intercept	518.03	37.44	13.84	<0.001
AR(1)	0.91	0.03	26.40	<0.001
MA(1)	–0.45	0.08	–5.93	<0.001
SAR(1)	–0.35	0.08	–4.51	<0.001
linear trend	0.18	0.24	0.73	0.469

Theft from vehicle in San Francisco, CA	estimate	SE	statistic	p value
week 2 of the year	25.08	19.29	1.30	0.195
week 3 of the year	43.89	21.22	2.07	0.040
week 4 of the year	–30.62	24.73	–1.24	0.218
week 5 of the year	–26.38	24.40	–1.08	0.281
week 6 of the year	37.74	25.49	1.48	0.141
week 7 of the year	–6.68	26.51	–0.25	0.801
week 8 of the year	43.24	27.40	1.58	0.117
week 9 of the year	28.15	30.41	0.93	0.356
week 10 of the year	14.96	30.04	0.50	0.619
week 11 of the year	4.94	28.69	0.17	0.864
week 12 of the year	20.79	30.16	0.69	0.492
week 13 of the year	50.68	30.73	1.65	0.101
week 14 of the year	–12.66	31.20	–0.41	0.685
week 15 of the year	–19.22	31.68	–0.61	0.545
week 16 of the year	2.32	32.19	0.07	0.943
week 17 of the year	–23.61	33.74	–0.70	0.485
week 18 of the year	–60.91	32.78	–1.86	0.065
week 19 of the year	–90.56	33.04	–2.74	0.007
week 20 of the year	–45.42	33.30	–1.36	0.175
week 21 of the year	–20.18	33.53	–0.60	0.548
week 22 of the year	–41.83	33.74	–1.24	0.217
week 23 of the year	–69.06	33.68	–2.05	0.042
week 24 of the year	–23.13	33.94	–0.68	0.497
week 25 of the year	–52.31	33.92	–1.54	0.125
week 26 of the year	–43.16	33.85	–1.27	0.204
week 27 of the year	–75.25	33.82	–2.23	0.028
week 28 of the year	–57.17	33.80	–1.69	0.093
week 29 of the year	45.57	33.76	1.35	0.179
week 30 of the year	60.17	33.67	1.79	0.076
week 31 of the year	–60.66	35.75	–1.70	0.092
week 32 of the year	–33.07	33.43	–0.99	0.324
week 33 of the year	12.75	33.29	0.38	0.702
week 34 of the year	75.15	33.12	2.27	0.025
week 35 of the year	45.18	32.88	1.37	0.171
week 36 of the year	38.44	34.85	1.10	0.272
week 37 of the year	38.45	32.37	1.19	0.237
week 38 of the year	59.11	32.07	1.84	0.067
week 39 of the year	85.00	31.68	2.68	0.008
week 40 of the year	8.50	31.28	0.27	0.786
week 41 of the year	29.58	30.82	0.96	0.339
week 42 of the year	21.39	30.32	0.71	0.482
week 43 of the year	4.80	29.74	0.16	0.872
week 44 of the year	18.72	29.10	0.64	0.521
week 45 of the year	–21.62	30.92	–0.70	0.486
week 46 of the year	0.39	27.56	0.01	0.989
week 47 of the year	–17.29	26.62	–0.65	0.517
week 48 of the year	36.26	25.55	1.42	0.158
week 49 of the year	71.74	24.32	2.95	0.004
week 50 of the year	29.46	22.92	1.29	0.200
week 51 of the year	84.70	21.24	3.99	<0.001
week 52 of the year	56.53	19.23	2.94	0.004
holiday in week	18.42	12.16	1.52	0.132

Theft from vehicle in Tucson, AZ	estimate	SE	statistic	p value
intercept	69.28	5.57	12.44	<0.001
AR(1)	0.93	NA	NA	NA
AR(2)	–0.02	0.09	–0.17	0.863
MA(1)	–0.57	NA	NA	NA
SAR(1)	–0.43	NA	NA	NA
linear trend	0.09	0.03	2.68	0.008
week 2 of the year	–7.02	4.13	–1.70	0.091
week 3 of the year	–9.92	4.40	–2.26	0.026
week 4 of the year	0.18	5.04	0.04	0.972
week 5 of the year	10.71	4.81	2.23	0.027
week 6 of the year	–8.36	4.96	–1.68	0.094
week 7 of the year	–3.65	5.10	–0.71	0.476
week 8 of the year	–3.17	5.21	–0.61	0.543
week 9 of the year	–2.93	5.85	–0.50	0.617
week 10 of the year	2.74	5.54	0.49	0.622
week 11 of the year	–3.41	5.21	–0.65	0.515
week 12 of the year	–3.51	5.52	–0.64	0.525
week 13 of the year	–4.11	5.67	–0.72	0.470
week 14 of the year	3.29	5.72	0.58	0.566
week 15 of the year	–7.13	5.72	–1.25	0.214
week 16 of the year	–0.84	5.85	–0.14	0.886
week 17 of the year	–0.16	6.10	–0.03	0.979
week 18 of the year	3.60	5.86	0.61	0.540
week 19 of the year	–3.17	5.87	–0.54	0.590
week 20 of the year	–10.09	5.98	–1.69	0.094
week 21 of the year	–2.85	6.01	–0.47	0.636
week 22 of the year	–1.62	5.91	–0.27	0.785
week 23 of the year	–0.73	5.94	–0.12	0.903
week 24 of the year	–8.57	6.10	–1.41	0.162
week 25 of the year	–10.12	6.04	–1.68	0.096
week 26 of the year	–1.90	6.05	–0.31	0.753
week 27 of the year	–18.37	6.06	–3.03	0.003
week 28 of the year	–14.58	6.09	–2.39	0.018
week 29 of the year	5.47	6.02	0.91	0.365
week 30 of the year	–10.60	6.03	–1.76	0.081
week 31 of the year	–4.79	6.45	–0.74	0.459
week 32 of the year	–12.29	5.96	–2.06	0.041
week 33 of the year	–7.68	5.92	–1.30	0.196
week 34 of the year	–15.15	5.97	–2.54	0.012
week 35 of the year	–7.53	5.96	–1.26	0.208
week 36 of the year	–3.00	6.33	–0.47	0.636
week 37 of the year	–4.50	5.88	–0.76	0.446
week 38 of the year	–0.56	5.75	–0.10	0.923
week 39 of the year	–2.56	5.81	–0.44	0.660
week 40 of the year	–10.71	5.75	–1.86	0.065
week 41 of the year	–7.69	5.70	–1.35	0.179
week 42 of the year	–12.79	5.63	–2.27	0.025
week 43 of the year	–5.56	5.55	–1.00	0.318
week 44 of the year	–12.94	5.45	–2.37	0.019
week 45 of the year	–12.58	5.94	–2.12	0.036
week 46 of the year	–3.10	5.25	–0.59	0.555
week 47 of the year	–5.33	5.09	–1.05	0.297

Theft from vehicle in Tucson, AZ	estimate	SE	statistic	p value
week 48 of the year	–0.81	4.96	–0.16	0.871
week 49 of the year	–1.94	4.78	–0.41	0.686
week 50 of the year	–0.86	4.62	–0.19	0.853
week 51 of the year	–6.06	4.38	–1.38	0.169
week 52 of the year	11.11	4.12	2.70	0.008
holiday in week	–4.77	2.62	–1.82	0.070

Theft from vehicle in Washington, DC	estimate	SE	statistic	p value
intercept	221.84	14.68	15.11	<0.001
AR(1)	0.42	0.07	6.43	<0.001
AR(2)	0.30	0.07	4.58	<0.001
SAR(1)	–0.42	0.08	–5.48	<0.001
linear trend	–0.04	0.08	–0.55	0.581
week 2 of the year	2.30	10.05	0.23	0.820
week 3 of the year	24.79	10.64	2.33	0.021
week 4 of the year	41.87	13.15	3.18	0.002
week 5 of the year	13.15	12.96	1.01	0.312
week 6 of the year	21.47	13.58	1.58	0.116
week 7 of the year	22.14	14.04	1.58	0.117
week 8 of the year	17.87	14.34	1.25	0.215
week 9 of the year	2.74	15.74	0.17	0.862
week 10 of the year	–13.26	15.34	–0.86	0.389
week 11 of the year	3.59	14.49	0.25	0.805
week 12 of the year	1.20	15.17	0.08	0.937
week 13 of the year	12.56	15.34	0.82	0.414
week 14 of the year	–50.22	15.47	–3.25	0.001
week 15 of the year	–30.50	15.67	–1.95	0.053
week 16 of the year	–14.21	15.82	–0.90	0.371
week 17 of the year	–29.36	16.61	–1.77	0.079
week 18 of the year	–35.96	15.93	–2.26	0.025
week 19 of the year	–47.43	15.97	–2.97	0.003
week 20 of the year	–54.69	16.00	–3.42	<0.001
week 21 of the year	–30.22	16.07	–1.88	0.062
week 22 of the year	–20.10	16.14	–1.25	0.215
week 23 of the year	–27.67	16.04	–1.73	0.087
week 24 of the year	–31.10	16.15	–1.93	0.056
week 25 of the year	–24.57	16.09	–1.53	0.129
week 26 of the year	–29.24	16.04	–1.82	0.070
week 27 of the year	–27.95	16.04	–1.74	0.083
week 28 of the year	2.33	16.03	0.15	0.885
week 29 of the year	6.17	16.01	0.39	0.700
week 30 of the year	–3.16	16.00	–0.20	0.844
week 31 of the year	3.69	17.23	0.21	0.831
week 32 of the year	–11.37	15.96	–0.71	0.477
week 33 of the year	6.80	15.94	0.43	0.671
week 34 of the year	–9.39	15.93	–0.59	0.556
week 35 of the year	–6.23	15.91	–0.39	0.696
week 36 of the year	–24.97	17.12	–1.46	0.147
week 37 of the year	1.00	15.81	0.06	0.950
week 38 of the year	–9.63	15.77	–0.61	0.542
week 39 of the year	25.92	15.71	1.65	0.101

Theft from vehicle in Washington, DC	estimate	SE	statistic	p value
week 40 of the year	0.46	15.63	0.03	0.976
week 41 of the year	–7.65	15.53	–0.49	0.623
week 42 of the year	28.17	15.42	1.83	0.070
week 43 of the year	20.31	15.27	1.33	0.186
week 44 of the year	–19.52	15.08	–1.29	0.197
week 45 of the year	–8.46	16.16	–0.52	0.601
week 46 of the year	24.61	14.55	1.69	0.093
week 47 of the year	8.47	14.15	0.60	0.550
week 48 of the year	13.42	13.65	0.98	0.327
week 49 of the year	–3.37	12.94	–0.26	0.795
week 50 of the year	36.21	12.20	2.97	0.003
week 51 of the year	29.21	10.64	2.75	0.007
week 52 of the year	45.32	10.05	4.51	<0.001
holiday in week	–6.79	6.37	–1.07	0.288

References

Hyndman, Rob J, and Yeasmin Khandakar. 2008. "Automatic Time Series Forecasting: The forecast Package for R." *Journal of Statistical Software* 27 (3): 1–22. https://doi.org/10.18637/jss.v027.i03.

Hyndman, Rob J., and Anne B. Koehler. 2006. "Another Look at Measures of Forecast Accuracy." *International Journal of Forecasting* 22 (4): 679–88. https://doi.org/10.1016/j.ijforecast.2006.03.001.
